# The Middle Pleistocene vertebrate fauna from Khok Sung (Nakhon Ratchasima, Thailand): biochronological and paleobiogeographical implications

**DOI:** 10.3897/zookeys.613.8309

**Published:** 2016-08-30

**Authors:** Kantapon Suraprasit, Jean-Jacques Jaeger, Yaowalak Chaimanee, Olivier Chavasseau, Chotima Yamee, Pannipa Tian, Somsak Panha

**Affiliations:** 1 Institut International de Paléoprimatologie et de Paléontologie Humaine: Evolution et Paléoenvironnements, UMR CNRS 7262, Université de Poitiers, 6 rue Michel Brunet, 86022 Poitiers, France; 2 Biological Sciences Program, Faculty of Science, Chulalongkorn University, Bangkok 10330, Thailand; 3 Animal Systematics Research Unit, Department of Biology, Faculty of Science, Chulalongkorn University, Bangkok 10330, Thailand; 4 Department of Mineral Resources, Rama VI Road, Bangkok, 10400, Thailand

**Keywords:** Large mammals, taxonomy, Ailuropoda–Stegodon assemblage, paleobiogeography, late Middle Pleistocene, Quaternary, northeastern Thailand, mainland Southeast Asia

## Abstract

The fluviatile terrace deposits of Khok Sung, Nakhon Ratchasima province, have yielded more than one thousand fossils, making this the richest Pleistocene vertebrate fauna of Thailand. The excellent preservation of the specimens allows precise characterization of the faunal composition. The mammalian fauna consists of fifteen species in thirteen genera, including a primate, a canid, a hyaenid, proboscideans, rhinoceroses, a suid, cervids, and bovids. Most species correspond to living taxa but globally (Stegodon cf. orientalis) and locally (*Crocuta crocuta ultima*, *Rhinoceros unicornis*, *Sus barbatus*, and *Axis axis*) extinct taxa were also present. The identification of *Axis axis* in Khok Sung, a chital currently restricted to the Indian Subcontinent, represents the first record of the species in Southeast Asia. Three reptilian taxa: Crocodylus cf. siamensis, *Python* sp., and *Varanus* sp., are also identified. Faunal correlations with other Southeast Asian sites suggest a late Middle to early Late Pleistocene age for the Khok Sung assemblage. However, the Khok Sung mammalian fauna is most similar to that of Thum Wiman Nakin, dated to older than 169 ka. The Khok Sung large mammal assemblage mostly comprises mainland Southeast Asian taxa that migrated to Java during the latest Middle Pleistocene, supporting the hypothesis that Thailand was a biogeographic pathway for the Sino-Malayan migration event from South China to Java.

## Introduction

**Figure 1.**
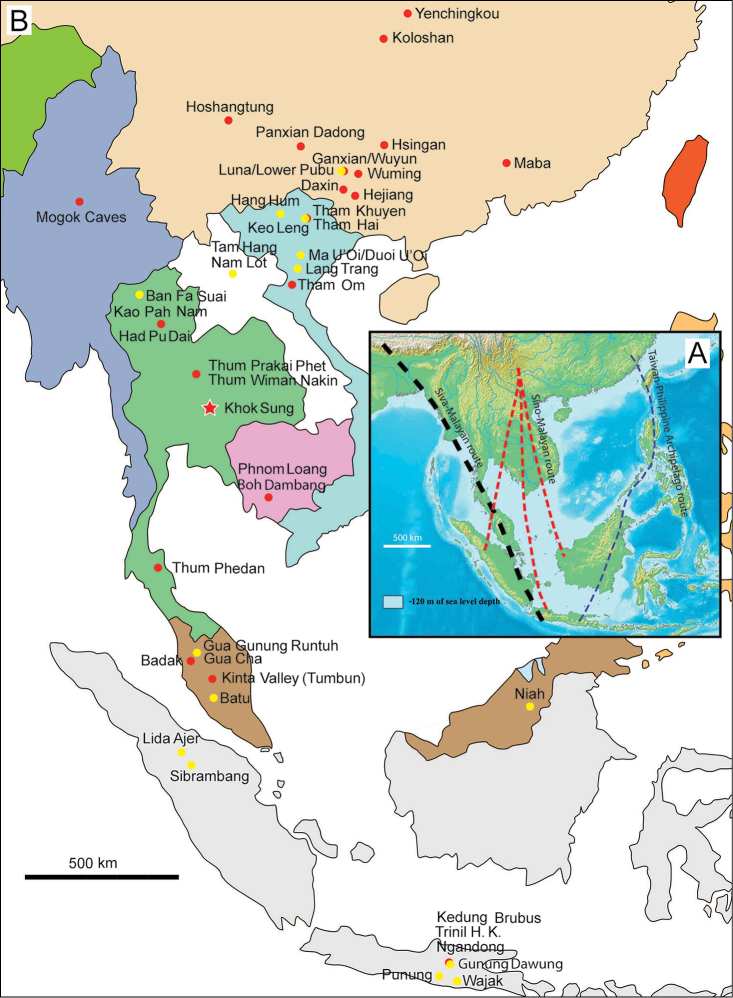
Map of Southeast Asia showing **A** the Sundaland boundaries and the migration route hypothesis: Siva-Malayan route (black), Sino-Malayan route (red), and Taiwan-Philippine Archipelago route (blue) and **B** the location of the Khok Sung sand pit (star) and other Middle (red circle) and Late (yellow circle) Pleistocene sites. The Sunda shelf boundaries at the sea level about 120 m lower than the present day are compiled from Voris (2000). Some Middle Pleistocene sites in South China and central Eastern China are shown in the map. Only Gua Cha (Peninsular Malaysia) is Holocene in age (Groves 1985, Bulbeck 2003).

In the Pleistocene, mammalian faunas in mainland Southeast Asia as well as in South China are characterized by the occurrence of *Ailuropoda* (giant panda) and/or *Stegodon* (extinct proboscidean), also called “*Ailuropoda*–*Stegodon* faunal complex”. This faunal association is a characteristic of the long period ranging from the Early to Late Pleistocene (Kahlke 1961, Rink et al. 2008). The *Ailuropoda*–*Stegodon* complex is different in composition from the Pinjor assemblage in the Indian Subcontinent (Marwick 2009) and likely originated in mainland China. In Java, mammalian faunas are characterized by the *Stegodon*-*Homo erectus* complex. This faunal association is supposed to have migrated via the Siva-Malayan route (Fig. 1A), from Indo-Pakistan to Java, during the Early Pleistocene (von Koenigswald 1935, de Vos 1995, 2007, de Vos and Long 2001, Delfino and de Vos 2010). Unfortunately, fossil records in mainland Southeast Asia do not allow the assessment of these early dispersal events because of the scarcity of Early Pleistocene sites. The only described Early Pleistocene mammal fossil assemblages are from terraces along the Irrawaddy River in Myanmar (Colbert 1943) and probably from the cave of Pha Bong in northern Thailand (Bocherens et al. in press). During the Middle Pleistocene, it has long been known that there were significant faunal exchanges that occurred between mainland Southeast Asia and Indonesian islands. Two migration routes, known as “Sino-Malayan”, are hypothesized (Fig. 1A): an insular pathway via the Philippines proposed by von Koeningswald (1938) and a continental pathway via Thailand, Myanmar, and Cambodia proposed by de Terra (1943). Recent studies on the paleogeographical affinities of Middle Pleistocene large mammals suggest that the latter route is most consistent (Tougard 2001, van den Bergh et al. 2001). The sea floor between Taiwan and the Philippine Archipelago was too deep for the emergence of a land bridge, and thus did not allow a dispersal route for large mammals during the Middle Pleistocene. This interpretation is also supported by a high number of endemic species that occur in Philippine Archipelago (Heaney 1985, Corbet and Hill 1992).

Dating from the Middle to Late Pleistocene, there are numerous paleontological and archaeological sites with mammalian fossil faunas discovered in Southeast Asian mainland (Indochinese) and islands (Sundaic) (Fig. 1B). The fossiliferous localities in Southeast Asia include the Mogok caves in Myanmar (Colbert 1943), Tham Khuyen, Tham Om and Tham Hai in Vietnam (Olsen and Ciochon 1990), Phnom Loang and Boh Dambang (Beden and Guérin 1973, Demeter et al. 2013) in Cambodia, Tambun in Malaysia (Hooijer 1962, Medway 1972), and Trinil H. K. (Hauptknochenschicht) and Kedung Brubus in Java (van den Bergh et al. 2001), all regarded as being Middle Pleistocene in age. Thailand is a critical position because it is located at the intermediate zone between different mammal communitites from South China and from Java (Lekagul and McNeely 1988, Corbet and Hill 1992, Tougard 1998, 2001). Studies on Thai Middle Pleistocene faunas are therefore crucial to understand the distribution patterns of large mammals across Southeast Asia. However, the information regarding the species composition, chronology, and paleogeographical affinitites of Thai Pleistocene faunas is poorly known due to the inappropriate taxonomic identification, to the lack of radiometric dating, and to the scarcity of substantial fossil sites, compared to that of other Southeast Asian countries.

An ongoing survey of Pleistocene deposits in Thailand has led to the discovery of numerous mammalian fossils by the Thai-French paleontological team in limestone caves from the northern to southern part of the country. Several fissure-filling and cave deposits: Thum Wiman Nakin (Ginsburg et al. 1982, Chaimanee and Jaeger 1993, Chaimanee 1998, Tougard 1998), Thum Phra Khai Phet (Tougard 1998, 2001, Filoux et al. 2015), Had Pu Dai (Pramankij and Subhavan 2001), Kao Pah Nam (Pope et al. 1981), and Thum Phedan (Yamee and Chaimanee 2005) (Fig. 1), have been dated to the Middle Pleistocene. However, only the Thum Wiman Nakin cave yielded a tooth of *Homo* sp. (Tougard et al. 1998). This fossil site was dated as older than 169 ka using U-series geochronology on the stalagmitic floor above the fossiliferous layers (Esposito et al. 1998, 2002). The ages of the other caves are solely established based on faunal assemblages. Unlike cave deposits, the Pleistocene terrace sequence in mainland Southeast Asia is rare.

**Figure 2.**
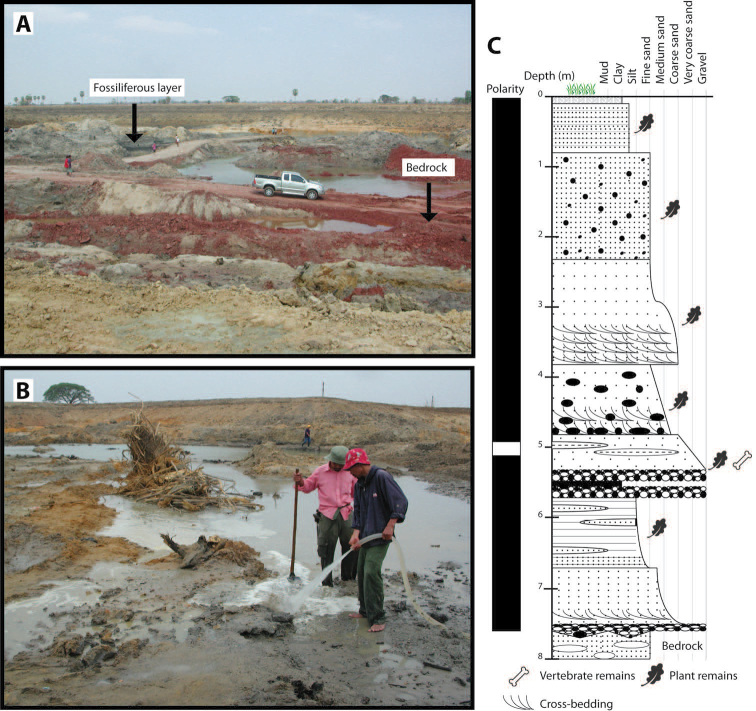
Locality of Khok Sung: **A** the sand pit during the paleontological excavation **B** the location of vertebrate fossils **C** the lithostratigraphic and paleomagnetic sections (modified from Suraprasit et al. 2015).

In 2005, the Khok Sung sand pit (Nakhon Ratchasima province, northeastern Thailand) was excavated (Fig. 2). This locality, an ancient fluviatile terrace, constitutes the richest Pleistocene vertebrate fauna of Thailand with thousand vertebrate remains. All vertebrate fossils were collected from layers of sand and gravels rich in organic matter, around 6-8 m below the surface (Fig. 2C) (for the detailed sedimentology see Duangkrayom et al. (2014) and Suraprasit et al. (2015)). The Khok Sung fauna is tentatively attributed to the late Middle Pleistocene, either 188 ka or 213 ka, based on paleomagnetic data and the occurrence of the spotted hyaena *Crocuta crocuta ultima* (Suraprasit et al. 2015). The Khok Sung locality yielded a unique and diverse fossil assemblage of plant remains, fish, reptiles, and mammals. Plant remains (fruits, seeds, leaves, wood, tubers, ambers, and pollens) suggest the presence of tropical mixed deciduous and dry green forests (Grote 2007). However, most of these fossil plants were possibly transported by the river and might have corresponded to the surrounding upland vegetation (Suraprasit et al. 2015). Some reptilian fossils were also described including turtles: Batagur cf. trivittata, *Heosemys annandalii*, Heosemys cf. grandis, and *Malayemys* sp., soft-shelled turtles: *Chitra* sp. and cf. *Amyda* sp. (Claude et al. 2011), and a gavial, Gavialis cf. bengawanicus (Martin et al. 2012). The mammalian assemblage consists of rhinoceroses, pigs, bovids, cervids, and an extinct elephant *Stegodon*, whose taxonomic attribution in generic and specific levels is poorly known (Chaimanee et al. 2005). In this paper, we provide taxonomic descriptions of vertebrate fossils from Khok Sung, focusing mainly on mammals. The fauna is compared with other contemporaneous Southeast Asian sites, and the results are used to propose a biochronological and paleobiogeographic framework for the fauna.

## Material and methods

### Fossil collecting and material

The sand pit was open for the pond construction (approximately 50 m long × 50 m wide × 8 m deep) (Fig. 2A). Fossils were collected, while the water was pumped out of the sand pit. All fossils observed in the field are macrofossils. Numerous vertebrate fossils were additionally searched, using the water spraying technique to remove covered sediments on the surface of the fossiliferous layer (Fig. 2B). Unfortunately, sieving methods for microvertebrate recovery were not used during the fossil excavation and this locality is no longer accessible due to the flooding.

All fossil specimens are housed at the Khok Sung local museum (Nakhon Ratchasima) and at the Department of Mineral Resources
(DMR) (Bangkok). Individual fossils are catalogued with the collection (DMR), locality (KS), and unique specimen number, respectively. The comparative material is from the recent and fossil vertebrate collections housed at the following natural history museums and institutes:

iPHEP Institut International de Paléoprimatologie et de Paléontologie Humaine: Evolution et Paléoenvironnements, Université de Poitiers (Poitiers, France)

IVPP
Institute of Vertebrate Paleontology and Paleoanthropology (Beijing, China)

NHMP
Natural History Museum Prague (Prague, Czech Republic)

NMW Naturhistorisches Museum Wien (Vienna, Austria)

MNHN-ZMO Zoological collection of mammals and birds, Muséum National d’Histoire Naturelle (Paris, France)

RMNH DUB Dubois collection, Rijksmuseum van Natuurlijke Histoire (Leiden, Netherlands)

THNHM-M Mammal collection, Thailand Natural History Museum (Pathum Thani, Thailand)

ZIN
Zoological Institute, Russian Academy of Sciences (St. Petersburg, Russia)

ZSM
Zoologische Staatssammlung München (Munich, Germany)

### Dental terminology and taxonomic nomenclature

**Figure 3.**
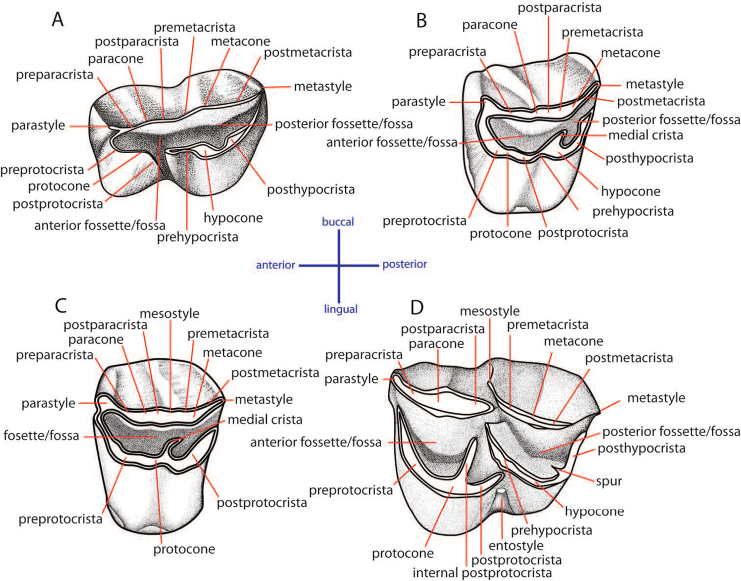
Dental nomenclatures of upper cheek teeth of ruminants: **A** upper second deciduous premolar **B** upper third premolar **C** upper fourth premolar **D** upper third molar. The dental terminology is modified from Heintz (1970), Gentry et al. (1999) and Bärmann and Rössner (2011).

**Figure 4.**
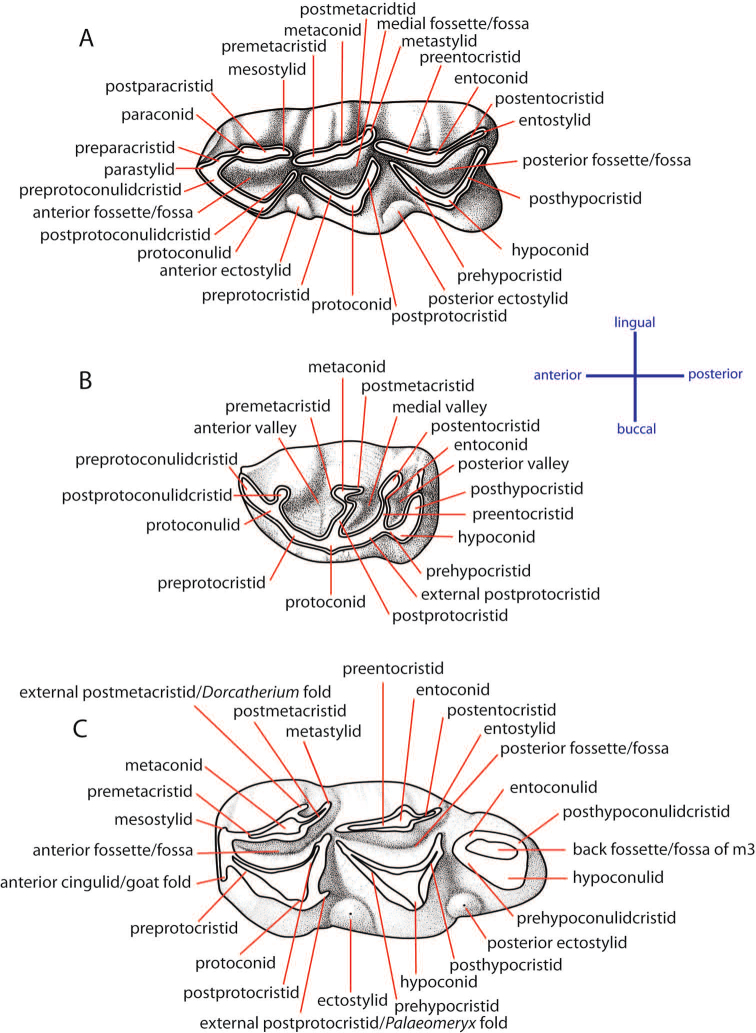
Dental nomenclatures of lower cheek teeth of ruminants: **A** lower fourth deciduous premolar **B** lower fourth premolar **C** lower third molar. The dental terminology is compiled from Heintz (1970), Gentry et al. (1999), and Bärmann and Rössner (2011).

The dental nomenclature follows van den Bergh (1999) for the proboscideans, Yan et al. (2014) for the rhinoceroses, and van der Made (1996) for the suids. The dental nomenclature for the ruminants is modified from Heintz (1970), Gentry et al. (1999), and Bärmann and Rössner (2011) (Figs 3 and 4). The taxonomic nomenclature of extant mammals follows Groves and Grubb (2011) for the ungulates and the systems of the IUCN Red List of Threatened Species (IUCN 2015) for primates, carnivores, elephants, and other vertebrates. The family-level identification of postcranial remains of mammals is based on the atlases of France (2009) and Brown and Gustafson (2000).

### Measurements

**Figure 5.**
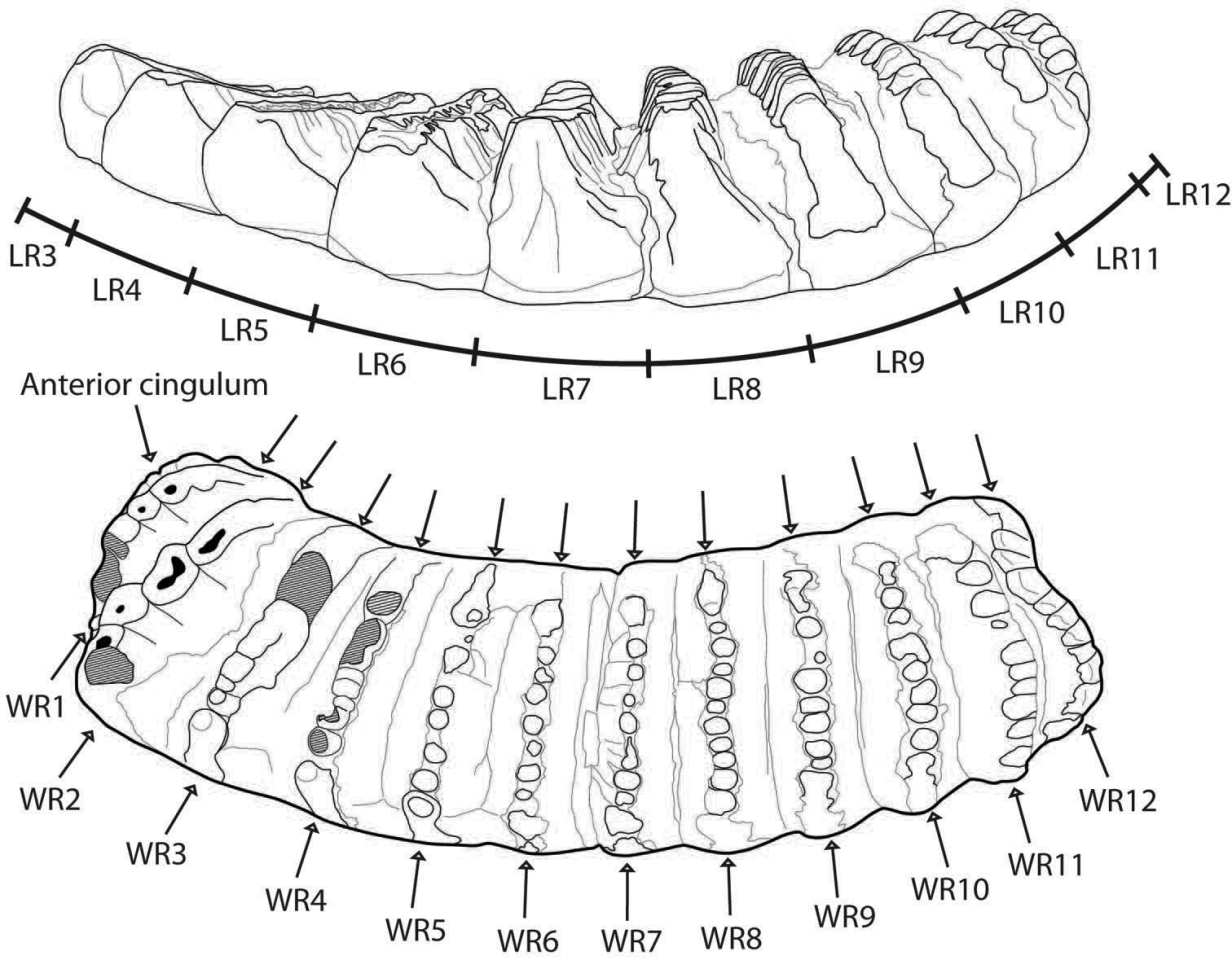
Parameters and measurement methods used for the lower third molar of *Stegodon*. Lengths and widths of the molar ridges are abbreviated as “LR” and “WR”, respectively. An illustration of two right m3 (lateral and occlusal views) of *Stegodon orientalis* is duplicated from the specimen IVPP V5216-15 (above) and IVPP V5216-13 (below).

All specimens were measured using digital callipers to the nearest 0.01 mm. The tooth dimensions for all mammals were measured at the base of the crown along the anterior-posterior margins for the maximum length (L) and from the labial (incisors and canines)/buccal (premolars/molars) to lingual margins for the maximum width (W). In the case of measurements of stegodontid cheek teeth, the methods and parameters used for molar and ridge dimensions were given in Fig. 5. The H/W index and the laminar frequency (LF) were calculated, using the formula proposed by van den Bergh (1999: p. 29–30). The ridge formula of stegodontids follows the original notation of Osborn (1942). Halfridges, whose width and height were 25% less than the succeeding or preceding ridge, at the anterior or posterior extremities of a stegodontid molar are not counted and abbreviated as “x”. The measurements of the cranial, mandibular, postcranial elements of mammals were taken, using the methods of von den Driesch (1976) (for metrical abbreviations, see Tab. 1).

**Table 1.** Abbreviations for postcranial bones from von den Driesch (1976).

**Scapula**	
**HS**	Height along the spine
**DHA**	Diagonal height from the most distal point of the scapula to the thoracic angle
**Ld**	Greatest dorsal length
**SLC**	Smallest length of the Collum scapulae (neck of the scapula)
**GLP**	Greatest length of the Processus articularis (glenoid process)
**LG**	Length of the glenoid cavity
**BG**	Breadth of the glenoid cavity
**Long bones**	
**GL**	Greatest length
**GLl**	Greatest length of the lateral part
**GLC**	Greatest length from the caput (head)
**PL**	Physiological length (for radius only)
**Ll**	Length of the lateral part
**Bp**	Greatest breadth of the proximal end
**BFp**	Greatest breadth of the Facies articularis proximalis (for radius only)
**BPC**	Greatest breadth across the coronoid process (=greatest breadth of the proximal articular surface) (for ulna only)
**SD**	Smallest breadth of diaphysis
**Dp**	Depth of the proximal end
**Bd**	Greatest breadth of the distal end
**BFd**	Greatest breadth of the Facies articularis distalis (for radius only)
**Dd**	Greatest breadth of the distal end
**DC**	Greatest depth of the Caput femoris
**DD**	Smallest depth of the diaphysis (for metapodials only)
**BT**	Greatest breadth of the trochlea (for humerus only)
**LO**	Length of the olecranon (for ulna only)
**DPA**	Depth across the Processus anconaeus (for ulna only)
**SDO**	Smallest depth of the olecranon (for ulna only)
**Pelvis**	
**GL**	Greatest length of one half
**LA**	Length of the acetabulum including the lip
**LS**	Length of the symphysis
**SH**	Smallest height of the shaft of ilium
**SB**	Smallest breadth of the shaft of ilium
**SC**	Smallest circumference of the shaft of ilium
**LFo**	Inner length of the foramen obturatum
**GBTc**	Greatest breadth across the Tubera coxarum–greatest breadth across the lateral angle
**GBA**	Greatest breadth across the acetabula
**GBTi**	Greatest breadth across the Tubera ischiadica
**SBI**	Smallest breadth across the bodies of the Ischia

### Body mass estimation

The body mass of ruminants was estimated using the equations of Janis (1990) based on the M2/m2 surface area ratio. The surface area of M2/m2 used here is the best body mass predictor according to the high correlations with the body mass for bovids (r^2^> 0.93) and cervids (r^2^> 0.95) (Janis 1990: table 16.8).

### Faunal similarity measures and cluster analysis

We compared differences in species compostion of Southeast Asian large mammal fauna during the Middle Pleistocene, using an analysis of the faunal similarity. According to unequal sampling conditions for our data, we applied two criteria for undertaking this analysis: localities are disqualified when they have fewer than 10 taxa identified at the species level and taxa are excluded when their appearances are still doubtful (i.e. poor taxonomic description or identification). We therefore selected Simpson’s Faunal Resemblance Index (FRI) because it has the smallest influence of sample sizes and emphasizes faunal resemblances (Simpson 1943, 1960). When fauna lists in several localities differ evidently in size, the Simpson’s FRI is the most useful tool for eliminating the effect of size differences between two faunas, compared to other indices (Simpson 1960). The Simpson’s FRI is also applied for analysing the faunal resemblances of vertebrate fossil records (e.g., Tsubamoto et al. 2004, Travouillon et al. 2006, Grossman et al. 2014). The formula of Simpson’s FRI is expressed as FRI (%) = (Nc / N1) × 100, where Nc is the number of identified taxa shared by two faunas and N1 is the number of identified taxa in the smaller of the two faunas (Simpson 1960). A higher score indicates a greater similarity between the faunas. We performed a dataset, transformed into a similarity matrix, to generate the dendrogram using the “PAST”statistical software version 1.61 (Hammer et al. 2001). We selected an Unweighted Pair-Group Method with Arithmetic Mean (UPGMA) as cluster algorithms for our analysis because the dendrogram represents higher values of cophenetic correlation coefficient than the others.

## Systematic paleontology

### Class MAMMALIA Linnaeus, 1758

#### Order PRIMATES Linnaeus, 1758

##### Suborder HAPLORRHINI Pocock, 1918

###### Family CERCOPITHECIDAE Gray, 1821

####### Genus *Macaca* Lacépède, 1799

######## Macaca sp.

Taxon classificationAnimaliaPrimatesCercopithecidae

######### Referred material.

A right tibia, DMR-KS-05-04-04-1.

######### Material description.

**Figure 6.**
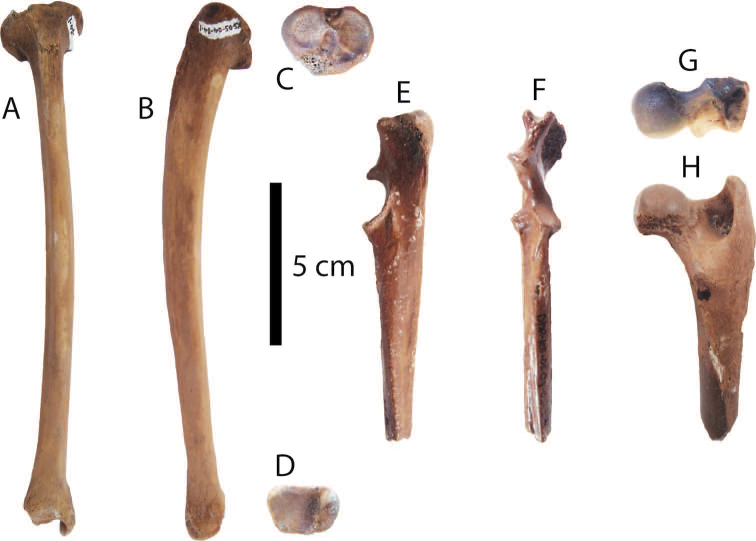
Postcranial remains of *Macaca* sp. **A–D** and *Cuon* sp. **E–H** from Khok Sung: **A–D** DMR-KS-05-04-04-1, a right tibia in anterior (**A**), medial (**B**), proximal (**C**), and distal (**D**) views **E–F** DMR-KS-05-04-11-34, a right ulna in medial (**E**) and anterior (**F**) views **G–H** DMR-KS-05-04-28-13, a right femur in proximal (**G**) and posterior (**H**) views.

The right tibia is complete (Fig. 6A–D) and elongated (for measurements, see Appendix 1). On the proximal articular surface, the medial condyle is as large as the lateral one. The lateral condyle is convex anteroposteriorly (Fig. 6C). The posteromedial margin of the lateral condyle lacks a notch that indicates a single meniscus attachment. At the proximal end, the tibial tuberosity is developed. The shaft is elongated, anteriorly and laterally bowed, and not anteroposteriorly compressed (Fig. 6A, B). Distally, the trochlear surface is trapezoid in outline (Fig. 6D). The medial malleolus is well-developed and projects more anteriorly than posteriorly. The medial and lateral parts of the trochlear surface are equally separated by a weak median keel.

######### Taxonomic remarks and comparisons.

Tibial morphology is relatively conservative within and among primates. Particularly, the morphological differences of tibiae among cercopithecoids are minimal (Turley et al. 2011). The distal part of tibiae of arboreal primates (including *Hylobates* and all arboreal cercopithecoids) is characterized by more rounded borders of the trochlear surface and a convex proximal border of the medial malleolus joining the trochlear surface (Tallman et al. 2013). The specimen DMR-KS-05-04-04-1 shows typical characters of the recent cercopithecoids whose tibial shaft is less mediolaterally compressed than those of great apes. However, the tibia from Khok Sung represents compatible dimensions with the tibiae of *Hylobates* (gibbon), *Presbytis* (surili), and *Macaca* (macaque). We suggest here to make a distinction between these genera based on the ratios of the greatest length of the tibia to the length or width of the proximal tibia (GL/Bp or GL/Dp). Based on these indices, the Khok Sung tibia falls within the range of recent *Macaca* (Tab. 2). According to the ratios, the shaft of both the surilis and gibbons is more elongated, compared to that of macaques. The distal tibia of DMR-KS-05-04-04-1 also shares some additional characters with that of macaques such as the poorly developed ball-shaped convexity and -articular facet (Sondaar et al. 2006) and the shape of the trochlear surface (Tallman et al. 2013: Fig. 5). We therefore attribute this material to *Macaca* sp.

**Table 2.** Ratios of the greatest lengths of tibiae (GL) to the lengths and widths of proximal and distal tibiae (Bp, Dp, Bd, and Dd) of Khok Sung macaques compared to recent Southeast Asian primates.

	DMR-KS-05-04-04-1	*Presbytis* (N = 30)			*Hylobates* (N = 24)			*Macaca* (N = 71)		
		Max	Min	Mean	Max	Min	Mean	Max	Min	Mean
**GL/Bp**	6.09	7.70	6.76	7.29	7.52	6.06	7.01	6.56	4.55	5.61
**GL/Dp**	7.81	9.89	8.15	9.07	9.95	7.96	9.43	9.62	6.36	7.67
**GL/Bd**	9.25	12.38	10.26	11.37	14.49	9.01	11.31	10.84	7.20	8.79
**GL/Dd**	12.94	16.21	12.75	14.13	16.79	10.94	14.50	12.77	7.69	10.78

#### Order CARNIVORA Bowdich, 1821

##### Family CANIDAE Fischer de Waldheim, 1817

###### Genus *Cuon* Hodgson, 1838

####### Cuon sp.

Taxon classificationAnimaliaCarnivoraCanidae

######## Referred material.

A right ulna, DMR-KS-05-04-11-34; a right femur, DMR-KS-05-04-28-13.

######## Material description.

DMR-KS-05-04-11-34 is a half proximal ulna preserving complete parts from the olecranon to the midshaft (Fig. 6E, F). The olecranon tuber is well-developed. The upper margin of the olecranon is concave and possesses a slightly higher posterior part that extends laterally. The anconeal process is distinct. The medial and lateral coronoid processes diverge laterally (Fig. 6F). The trochlear notch is deep, forming nearly a semicircular surface for articulation (Fig. 6E).

The right femur preserves a complete proximal part and broken shaft (Fig. 6G, H). The greater trochanter is as high as the upper surface of the rounded femoral head. The intertrochanteric crest is straight and nearly oriented vertically (Fig. 6H). The upper border of the neck is flat. The lesser trochanter projects anteriorly and is situated at about 1.5 cm below the femoral head.

######## Taxonomic remarks and comparisons.

The proximal ulna of canids is characterized by a bilobed and laterally compressed olecranon process, well-developed anconeal and lateral coronoid processes, and a laterally compressed shaft. The proximal crest of the olecranon is grooved anteriorly, but enlarged and rounded posteriorly (Tong et al. 2012). Pionnier-Capitan et al. (2011) suggested that in medial view the posteroproximal tuberosity of the olecranon of *Canis* is more proximally developed than in *Cuon*. The posteroproximal tuberosity of the Khok Sung ulna is as developed as that of *Cuon*. Furthermore, based on our comparisons with extant specimens, the Khok Sung canid ulna resembles that of *Cuon alpinus* because the olecranon bends more medially and the posterior border of the olecranon is straighter than those observed in *Canis lupus*. The Khok Sung specimen is slightly smaller than the recent *Cuon alpinus* (Tab. 3). However, it is much smaller than recent and fossil *Canis lupus*, as well as the paleosubspecies *Cuon alpinus caucasicus* (Tab. 3).

**Table 3.** Measurements (in millimetres) of ulnae and femurs of Khok Sung and other extant and fossil canids. * indicates a subadult individual. Metrical data of fossil canids are from Baryshnikov (2012, 2015).

**Ulna**							
**Specimen no.**	**Taxa**	**Age**	**Locality**	**LO**	**DPA**	**SDO**	**BPC**
DMR-KS-05-04-11-34	*Cuon* sp.	late Middle Pleistocene	Khok Sung, northeastern Thailand	15.16	18.51	15.21	11.65
NMW 1531*	*Canis lupus*	Recent	Eastern India	29.91	24.11	18.38	15.65
				29.29	24.43	18.43	15.33
ZIN 37274-27	*Canis lupus*	Late Pleistocene	Geographical Society Cave, Russia	–	32.30	27.80	–
NHMP R5387	*Canis lupus*	Late Pleistocene	Srbsko Chlum-Komín Cave, Czech Republic	–	34.80	27.60	–
NMW B5319	*Cuon alpinus*	Recent	Java, Indonesia	19.23	19.37	16.36	14.43
				19.74	19.29	16.33	14.07
ZIN 36733-1	*Cuon alpinus caucasicus*	Late Pleistocene	Kudaro 1 Cave, Southern Ossetia, Caucasus	–	–	–	18.30
ZIN 36739	*Cuon alpinus caucasicus*	Late Pleistocene	Kudaro 1 Cave, Southern Ossetia, Caucasus	–	32.20	–	17.20
ZIN 36698-1	*Cuon alpinus caucasicus*	Late Pleistocene	Kudaro 3 Cave, Southern Ossetia, Caucasus	–	28.70	24.50	18.90
ZIN 36697-2	*Cuon alpinus caucasicus*	Late Pleistocene	Kudaro 3 Cave, Southern Ossetia, Caucasus	–	34.00	29.50	21.50
ZIN 36677-2	*Cuon alpinus caucasicus*	Late Pleistocene	Kudaro 3 Cave, Southern Ossetia, Caucasus	–	33.60	28.60	21.70
ZIN 31241-3	*Cuon alpinus caucasicus*	Late Pleistocene	Kudaro 3 Cave, Southern Ossetia, Caucasus	–	30.30	26.50	17.00
ZIN 36670	*Cuon alpinus caucasicus*	Late Pleistocene	Kudaro 3 Cave, Southern Ossetia, Caucasus	–	28.80	–	18.50
ZIN 36705-7	*Cuon alpinus caucasicus*	Late Pleistocene	Kudaro 3 Cave, Southern Ossetia, Caucasus	–	–	–	15.00
**Femur**							
**Specimen no.**	**Taxa**	**Age**	**Locality**	**Bp**	**Dp**	**DC**	**SD**
DMR-KS-05-04-28-13	*Cuon* sp.	late Middle Pleistocene	Khok Sung, northeastern Thailand	35.69	17.90	16.58	11.34
NMW 1531*	*Canis lupus*	Recent	Eastern India	35.05	16.70	16.75	10.43
				35.57	16.82	16.73	10.52
NMW B5319	*Cuon alpinus*	Recent	Java, Indonesia	31.03	15.95	16.62	11.66
				31.58	16.08	16.38	11.79
ZIN 36692-2	*Cuon alpinus caucasicus*	Late Pleistocene	Kudaro 3 Cave, Southern Ossetia, Caucasus	48.70	–	22.70	–
ZIN 36700-2	*Cuon alpinus caucasicus*	Late Pleistocene	Kudaro 3 Cave, Southern Ossetia, Caucasus	–	–	21.70	15.20

Living canids generally show a typical morphology of the proximal femur, characterized by their relatively vertical intertrochanteric crests, prominent lesser trochanter with the sharp crest extending downward along the shaft, moderately-sized greater trochanter, and slender shaft (France 2009, Tong et al. 2012). In *Canis lupus*, the lateral side of the caput femoris is obliquely prolonged towards the trochanteric fossa. The upper border of the neck is concave and shorter than those in *Cuon alpinus* (Ripoll et al. 2010). The femur DMR-KS-05-04-28-13 is canid-sized (Tab. 3) and is comparable in morphology to *Cuon alpinus*. For instance, the intertrochanteric crest is more oblique and straighter (nearly vertical and curved in *Canis lupus*), the caput femoris is round, and the upper border of the neck is long and flat (Ripoll et al. 2010).

Because the Khok Sung ulna and femur morphologically match better *Cuon alpinus* than *Canis lupus*, we identify these two postcranial specimens as belonging to *Cuon* sp.

#### Order PROBOSCIDEA Illiger, 1811

##### Family STEGODONTIDAE Osborn, 1918

###### Genus *Stegodon* Falconer and Cautley, 1857

####### Stegodon cf. orientalis

Taxon classificationAnimaliaProboscideaStegodontidae

Owen, 1870

######## Referred material.

A right DP4 (posterior part), DMR-KS-05-03-28-14; a left DP4 (anterior part), DMR-KS-05-03-19-7; a left M2, DMR-KS-05-03-29-1 (posterior part); a right M3, DMR-KS-05-03-22-19 (posterior part); a fragmentary tusk, DMR-KS-05-03-15-2; a left dp3 (anterior part), DMR-KS-05-04-01-8; two mandibles with m3—DMR-KS-05-03-08-1 (right) and DMR-KS-05-03-08-2 (left); a right humerus fragment (proximal part), DMR-KS-05-03-10-5; a left humerus, DMR-KS-05-03-10-6; two ulna fragments (proximal parts)—DMR-KS-05-03-09-7 and DMR-KS-05-03-10-2; a femoral head fragment, DMR-KS-05-03-10-3; a right femur, DMR-KS-05-03-10-4; a right tibia fragment (distal part), DMR-KS-05-03-10-3; a right fibula, DMR-KS-05-03-00-124; two pelvis fragments—DMR-KS-05-03-10-11 (right) and DMR-KS-05-03-10-12 (left); five vertebrae—DMR-KS-05-03-17-11, DMR-KS-05-03-10-7, DMR-KS-05-03-09-18, DMR-KS-05-03-10-1, and DMR-KS-05-03-28-20; a sacrum fragment, DMR-KS-05-03-10-8; two ribs—DMR-KS-05-03-10-13 and DMR-KS-05-03-10-14; three rib fragments—DMR-KS-05-03-09-6 (body), DMR-KS-05-03-09-45 (body), and DMR-KS-05-03-09-4 (head and neck).

######## Material description.

**Figure 7.**
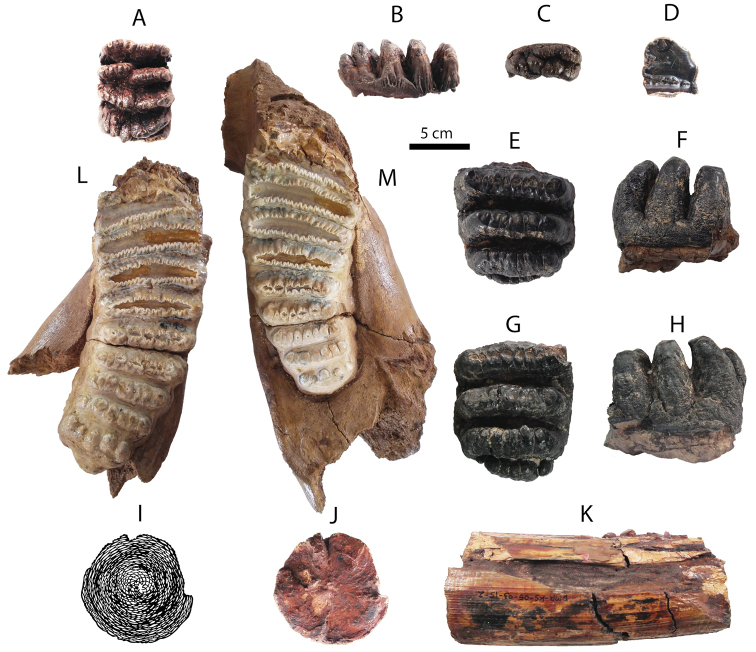
Dental remains of Stegodon cf. orientalis from Khok Sung: **A–B** DMR-KS-05-03-28-14, a right DP4 in occlusal (**A**) and buccal (**B**) views **C** DMR-KS-05-03-19-7, an anterior lobe of DP4 in occlusal view **D** DMR-KS-05-04-01-8, a left dp3 in occlusal view **E–F** DMR-KS-05-03-29-1, a left posterior fragment of M2 in occlusal (**E**) and buccal (**F**) views **G–H** DMR-KS-05-03-22-19, a right posterior fragment of M3 in occlusal (**G**) and buccal (**H**) views **I–K** DMR-KS-05-03-15-2, a fragmentary upper tusk in proximal (**I–J**) and dorsal (**K**) views **L** DMR-KS-05-03-08-1, a left mandible with m3 in occlusal view **M** DMR-KS-05-03-08-2, a right mandible with m3 in occlusal view.

**Upper dentition**: both fragments of DP4 (DMR-KS-05-03-28-14: Fig. 7A, B) and DMR-KS-05-03-19-7: Fig. 7C) are slightly worn and unworn respectively (for measurements, see Tab. 4). The first specimen lacks two or three anterior ridges, whereas the second specimen preserves only the anterior cingulum and the first ridge. DMR-KS-05-03-28-14 has a rectangular outline in occlusal view, a convex crown base in lateral view, and a posterior cingulum. These characters indicate that this specimen belongs to a posterior lobe of DP4. The buccal and lingual surfaces of ridges display subvertically developed grooves. A median cleft is well-developed and runs from anteriorly to posteriorly in the middle part of the tooth, starting from the halfway height of the crown. The second anterior ridge of DMR-KS-05-03-28-14 shows displacement between the pretrite and posttrite halves, a character sometimes present in deciduous molars of derived *Stegodon*. Each ridge bears ten to twelve mammillae.

**Table 4.** Measurements (in millimeters) of cheek teeth of Khok Sung proboscideans, including a number of preserved ridges (NR), lengths (L), widths (W), heights (H), enamel thickness (ET), H/W indices (100 × H/W), and laminar frequencies (LF). The laminar frequencies are expressed as the following formula: LF=n*100/d_l_ + n*100/d_b_ / 2, where “d_l_” and “d_b_” are referred to distances at the lingual and buccal side of the tooth, respectively, and “n” is equivalent to the number of ridges between two measuring points (van den Bergh 1999). * indicates measurements of the maximum length of the preservation according to incomplete specimens. The H/W index is calculated for each ridge. The laminar frequency is measured based on the maximum number of preserved ridges.

Specimen no.		NR	L	W	H	ET	H/W index	LF
**Stegodon cf. orientalis**								
DMR-KS-05-03-28-14	DP4	4	60.08	50.04	26.71	0.69–1.21	53.38–58.23	7.99
DMR-KS-05-03-19-7	DP4	1	18.65	49.89*	26.71	2.06	53.53	–
DMR-KS-05-03-29-1	M2	3	70.14*	78.83	55.18	1.62–3.06	70.00–73.34	4.61
DMR-KS-05-03-22-19	M3	3	90.43*	84.66	46.14	3.77–4.35	57.33–62.71	3.86
DMR-KS-05-04-01-8	dp3	3	26.68*	26.09	12.08	1.82	46.30–47.76	10.41
DMR-KS-05-03-08-1	m3	8	245.86*	95.66	41.50	3.41–6.87	43.38–51.77	3.91
DMR-KS-05-03-08-2	m3	8	247.78*	95.57	42.56	3.39–6.54	44.53–52.21	3.94
***Elephas* sp.**								
DMR-KS-05-03-17-12	Lower molar	2	41.04*	66.77*	108.94	2.48–3.30	163.16–165.18	10.61

DMR-KS-05-03-29-1 (M2) preserves three posterior ridges with a small cingulum (Fig. 7E, F and Tab. 4). Two anterior ridges bear slightly worn mammillae with stronger abrasion on the buccal side. The posterior-most ridge is unworn and reduced in width. The outline of the buccal side is concave in occlusal view and the base of the crown is nearly straight in lateral view. The median cleft is weakly developed. The number of the mammillae on each ridge ranges from eight to eleven.

DMR-KS-05-03-22-19 (M3) preserves only three posterior ridges with a cingulum (Fig. 7G, H and Tab. 4). The ridges are slightly worn with more abraded buccal surfaces. The general outline of this tooth is similar to that of M2, but is comparatively wider and displays a more developed posterior cingulum. The median cleft is poorly developed. Each ridge consists of eight to ten mammillae.

A fragmentary tusk (DMR-KS-05-03-15-2) contains dentine (outer and inner layers), cementum, and a pulp cavity (Fig. 7I–K). It is slightly curved upward and sub-rounded in cross-section for both the proximal and the distal section. A median longitudinal groove is present on the dorsal surface. The Schreger pattern commonly developed in elephantoid tusks is visible on the inner dentine layer. The maximum length of DMR-KS-05-03-15-2 is 159.2 mm and the mediolateral and dorsoventral diameters of the proximal cross-section are 73.88 and 70.56 mm, respectively. The outline of the tusk (DMR-KS-05-03-15-2) resembles *Stegodon trigonocephalus* in its more medial-laterally than the dorso-ventrally compressed cross-section. The macroscopic distinctive features in cross-section are similar to *Stegodon sompoensis* (van den Bergh 1999) but show the incremental lines more obviously.

**Lower dentition**: DMR-KS-05-04-01-8 (dp3) is heavily worn and comprises three preserved ridges and an anterior cingulum (Fig. 7D and Tab. 4). The buccal part of the third ridge is broken but it is presumably wider than the second ridge. The dp3 is subrectangular in outline or tapers towards the anterior part. The lateral sides between the first and second ridges are distinctly constricted.

Two hemi-mandibles of the same individual (DMR-KS-05-03-08-1 and DMR-KS-05-03-08-2) are moderately well-preserved (Tab. 4). The completely erupted m3 has eight ridges with small posterior cingulids (Fig. 7L, M). The symphysis and most of the ramus are broken away. The mandibular corpus is robust. We estimate the total number of ridges to be eleven based on the position on the corpus of the anterior root that supports two first lophs in *Stegodon* (Saegusa et al. 2005). The anteriormost preserved ridge is thus the third ridge, broken at its anterior and lateral parts in both specimens. The third to sixth ridges are strongly worn, whereas more posterior ridges are successively less damaged by abrasion. Valleys between the ridges are moderately filled with abundant cement. There is no median cleft. The m3 is much more elongated and contains five mammillae on the posteriormost ridge. The mammillae increase in size successively from the anterior to posterior ridge.

**Figure 8.**
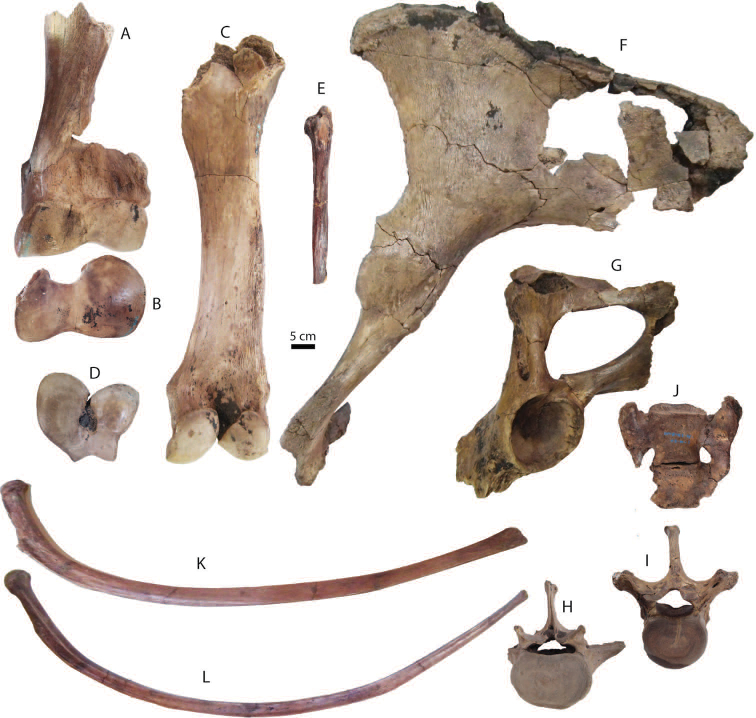
Postcranial remains of Stegodon cf. orientalis from Khok Sung: **A–B** DMR-KS-05-03-10-6, a left distal humerus in anterior (**A**) and distal (**B**) views **C–D** DMR-KS-05-03-10-4, a right femur posterior (**C**) and distal (**D**) views **E** DMR-KS-05-03-00-124, a right fibula in posterior view **F** DMR-KS-05-03-10-11, a right pelvis in dorsal view **G** DMR-KS-05-03-10-12, a left pelvis in lateral view **H** DMR-KS-05-03-09-18 and **I** DMR-KS-05-03-10-7, vertebrae in anterior view **J** DMR-KS-05-03-10-8, a sacrum in ventral view **K** DMR-KS-05-03-10-14 and **L** DMR-KS-05-03-10-13, ribs in anterior view.

**Postcranial remains**: postcranial elements include two humeri (Fig. 8A, B), two ulnae, two femora (Fig. 8C, D), a tibia, a fibula (Fig. 8E), two pelvis girdles (Fig. 8F, G), five vertebrae, a sacrum (Fig. 8J), and five ribs (Fig. 8K, L) (for measurements, see Appendix 1). All postcranial bones excluding some vertebrae belong to a single individual because they were found together in association with two mandibles with the m3 (DMR-KS-05-03-08-1 and DMR-KS-05-03-08-2) and show fully fused epiphyses. This individual is a senior adult due to the heavy wear on the anterior lophs on the m3. Only two vertebrae (DMR-KS-05-03-09-18: Fig. 8H and DMR-KS-05-03-10-7: Fig. 8I) were found in association with that individual. The specimen DMR-KS-05-03-26-38 is a juvenile because the vertebral body is not fused.

######## Taxonomic remarks and comparisons.

The proboscidean cheek teeth from Khok Sung are assigned to *Stegodon* because there are more than five ridges or loph(id)s on molars, V-shaped valleys between ridges on molars , and step-like worn surface reliefs on the enamel layer (Saegusa 1996, Saegusa et al. 2005). The Khok Sung material shows well-developed cheek tooth features of derived *Stegodon* (e.g., a greater number of ridges and mammillae, high filled cements between the ridges, and a high angled cliff on the enamel surfaces (step-like structure “type 3”, in Saegusa (1996)).

The morphologies and ridge sizes of upper molars from Khok Sung are congruent with Chinese *Stegodon orientalis* (Tabs 5–7). However, we suggest that some comparative upper third molars of *Stegodon orientalis* (e.g., IVPP V5216-9) represent a total ridge number of ten (excluding anterior and posterior halfridges), different from the ridge formula (×11× for this species) given by van den Bergh et al. (2008: table. 3). The ridge formula of the M3 of *Stegodon orientalis* therefore ranges from ten to eleven. The m3 of *Stegodon orientalis* commonly has a total number of twelve ridges (excluding anterior and posterior halfridges). According to the fact that only a few comparative specimens of the m3 of *Stegodon orientalis* are complete with the total ridge number of twelve, some of them (e.g., IVPP V1777 and IVPP V5216-16, based on our observations) display a total of 11 ridges (excluding anterior and posterior halfridges). In *Stegodon orientalis*, the number of ridges on the m3 thus ranges from eleven to twelve. *Stegodon insignis* has a total number of ridges ranging from eleven to thirteen (van den Bergh et al. 2008). The ridge formula of *Stegodon trigonocephalus trigonocephalus* is almost thirteen (excluding anterior and posterior halfridges) (van den Bergh 1999). Another subspecies, *Stegodon trigonocephalus praecursor*, has a lower number of ridges (×11×, van den Bergh et al. 2008: table. 3). The m3 of the Khok Sung stegodontid share a similar ridge formula (×11×) with *Stegodon orientalis* from South China and *Stegodon insignis* from Punjab (Siwaliks). But it differs from *Stegodon insignis* in having more delicately folded enamel, more pronounced curvature of the crown, and V-shaped valleys (between the two ridges) slightly less filled by cements. The ridge sizes of Khok Sung lower third molar are almost comparable to those of *Stegodon orientalis* and *Stegodon insignis*, but are distinctly larger than other derived *Stegodon* species from Indonesia (Tab. 8). We thus identify hereby all cheek teeth as belonging to Stegodon cf. orientalis.

**Table 5.** Ridge dimensions (lengths and widths in millimeters) of upper fourth deciduous premolars between Khok Sung *Stegodon* and *Stegodon orientalis*.

DP4	Ridge (from anterior to posterior)					
	1^st^	2^nd^	3^rd^	4^th^	5^th^	6^th^
**Stegodon cf. orientalis (Khok Sung)**						
Length	15.7	–	> 10.7	12.4	13.5	13.5
Width	49.9	–	49.9	50.0	49.7	48.4
Specimen measurements: DMR-KS-05-03-28-14 and DMR-KS-05-03-19-7						
***Stegodon orientalis* (×6×)**						
N	3	3	3	3	3	3
Length	12.3–16.2	15.3–19.7	14.3–20.4	13.3–18.4	12.6–16.2	11.1–16.5
Mean	14.1	17.0	17.4	16.1	15.0	13.6
N	3	3	2	3	3	3
Width	43.7–54.1	49.2–63.1	51.8–63.3	51.2–60.2	50.0–57.2	45.8–52.2
Mean	49.0	54.6	57.5	54.4	53.0	48.9
Specimen measurements: IVPP V1869, IVPP V1870, IVPP V5215-38, and IVPP RV39068						

**Table 6.** Ridge dimensions (lengths and widths in millimeters) of lower third deciduous premolars between Khok Sung *Stegodon* and *Stegodon orientalis*.

**dp3**	**Ridge (from anterior to posterior)**					
	**1^st^**	**2^nd^**	**3^rd^**	**4^th^**	**5^th^**	**6^th^**
**Stegodon cf. orientalis (Khok Sung)**						
Length	9.3	–	–	–	–	–
Width	25.5	> 26.1	–	–	–	–
***Stegodon orientalis* (×5×)**						
N	7	7	7	7	7	–
Length	7.2–10.8	6.5–10.3	9.6–12.9	10.9–12.0	10.0–14.0	–
Mean	8.5	9.0	11.0	11.5	12.6	–
N	7	7	7	7	7	–
Width	19.5–32.3	24.8–27.9	27.3–31.9	32.6–37.8	36.2–42.3	–
Mean	25.0	26.6	29.9	34.9	39.2	–
Specimen measurements: IVPP V1798, IVPP V1800, IVPP V1804, IVPP V1807, IVPP V1808, IVPP V1812, and IVPP V1815						
***Stegodon orientalis* (×6×)**						
N	5	5	5	5	5	5
Length	8.6–13.1	7.0–11.8	10.1–12.8	10.6–13.0	10.6–13.4	8.5–12.5
Mean	10.5	8.6	11.5	11.7	11.7	10.0
N	4	5	5	5	5	5
Width	23.7–31.1	26.8–32.1	29.1–34.7	33.1–41.1	36.7–47.3	36.0–52.4
Mean	27.3	28.9	31.5	36.8	41.3	40.4
Specimen measurements: IVPP1799, IVPP V1801, IVPP V1802, IVPP V1803, and IVPP V1816						

**Table 7.** Ridge dimensions (lengths and widths in millimeters) of upper second and third molars between Khok Sung *Stegodon* and *Stegodon orientalis*. The total ridge number of upper molars of Khok Sung stegodontids used for our comparisons follows that of *Stegodon orientalis*.

M2 and M3	Ridge (from anterior to posterior)										
	1^st^	2^nd^	3^rd^	4^th^	5^th^	6^th^	7^th^	8^th^	9^th^	10^th^	Posterior halfridge
**Stegodon cf. orientalis (Khok Sung)**											
DMR-KS-05-03-29-1 (M2)											
Length	–	–	–	–	–	28.2	23.9	19.7	–	–	–
Width	–	–	–	–	–	78.8	76.9	63.3	–	–	–
DMR-KS-05-03-22-19 (M3)											
Length	–	–	–	–	–	–	–	29.3	24.2	21.8	12.9
Width	–	–	–	–	–	–	–	80.5	77.6	70.1	49.7
***Stegodon orientalis* (M2) (×8×)**											
N	1	1	1	1	1	2	2	2	–	–	–
Length	23.4	25.0	30.7	25.4	22.1	20.3–22.5	20.5–22.0	15.4–17.7	–	–	–
Mean	–	–	–	–	–	21.4	21.2	16.6	–	–	–
N	1	1	1	1	1	2	2	2	–	–	–
Width	77.8	80.4	83.1	83.0	81.6	76.4–78.4	73.0–73.8	63.2–69.1	–	–	–
Mean	–	–	–	–	–	77.4	73.4	66.1	–	–	–
Specimen measurements: IVPP V1821 and IVPP V5216-5											
***Stegodon orientalis* (M3) (×10×)**											
N	3	3	3	2	1	1	1	2	2	2	2
Length	22.4–25.3	22.2–27.4	22.3–27.1	24.6–24.8	25.5	22.5	21.1	19.9–26.3	17.8–24.1	16.4–22.4	7.2–15.8
Mean	23.7	25.6	24.5	24.7	–	–	–	23.1	20.9	19.4	11.5
N	2	2	2	1	–	2	2	2	2	2	2
Width	85.4–91.5	88.3–96.7	84.9–101.3	100.4	–	81.4–87.1	83.3–85.7	75.0–87.9	65.4–89.5	57.4–81.8	38.6–54.6
Mean	88.4	92.5	93.1	–	–	84.3	84.5	81.4	77.5	69.6	46.6
Specimen measurements: IVPP V1772, IVPP V1775, IVPP V1763, and IVPP V5216-5											

**Table 8.** Ridge dimensions (lengths and widths in millimeters) of lower third molars of derived *Stegodon* in Southeast Asia. The ridge formula of each taxon follows van den Bergh et al. (2008: table. 3). The ridge number of *Stegodon insignis* is considered as representing a total of twelve.

**Lower third molar**	**Ridge (from anterior to posterior)**												
	**1^st^**	**2^nd^**	**3^rd^**	**4^th^**	**5^th^**	**6^th^**	**7^th^**	**8^th^**	**9^th^**	**10^th^**	**11^th^**		
**Stegodon cf. orientalis (Khok Sung) ((×?2)9×)**													
N	–	–	–	2	2	2	2	2	2	2	2		
Length	–	–	–	29.8–32.4	28.8–30.6	31.8–32.8	28.2–34.1	28.9–32.3	24.5–30.7	23.3–26.9	16.6–22.4		
Mean	–	–	–	31.1	29.7	32.3	31.2	30.6	27.6	25.1	19.5		
N	–	–	–	2	2	2	2	2	2	2	2		
Width	–	–	–	95.7–97.6	94.4–95.8	92.7–94.0	83.1–83.3	72.3–76.3	67.7–68.9	61.6–65.9	55.4–58.6		
Mean	–	–	–	96.7	95.1	93.4	83.2	74.3	68.3	63.8	57.0		
**Stegodon cf. orientalis (×11×)**													
N	–	2	2	2	2	2	2	2	2	2	2		
Length	–	26.0–31.7	26.4–33.8	23.3–34.4	25.5–33.1	28.6–31.7	24.8–39.4	26.8–36.4	25.6–31.0	21.1–24.3	15.4–15.9		
Mean	–	28.9	30.1	28.9	29.3	30.2	32.1	31.6	31.3	22.7	15.7		
N	–	1	2	1	1	2	2	2	2	2	2		
Width	–	82.43	68.0–86.1	88.34	90.11	72.4–93.1	72.0–91.9	71.2–88.3	64.6–80.1	54.8–63.6	41.8–43.1		
Mean	–	–	77.1	–	–	82.8	82.0	79.8	72.4	59.2	42.5		
Specimen measurements: IVPP V1777 and IVPP5216-16													
	**1^st^**	**2^nd^**	**3^rd^**	**4^th^**	**5^th^**	**6^th^**	**7^th^**	**8^th^**	**9^th^**	**10^th^**	**11^th^**	**12^th^**	
***Stegodon insignis* (×12×)**													
N	–	1	2	3	2	2	2	3	3	2	2	2	
Length	–	27.7	26.4–29.2	20.7–23.7	24.2	21.6–23.8	20.9–22.8	23.0–25.6	23.4–30.9	22.5–25.3	21.5–23.5	19.5–23.7	
Mean	–	–	27.8	22.1	24.2	22.7	21.9	24.6	26	23.9	22.5	21.6	
N	–	1	3	3	2	2	2	3	2	2	2	2	
Width	–	79.3	83.9–92.6	81.7–91.2	92.7–94.8	90.7–98.5	89.5–89.9	84.6–88.0	73.7–77.7	66.6–68.8	61.6–64.0	47.1–52.5	
Mean	–	–	87.4	88.0	93.8	94.6	89.7	85.9	75.7	67.7	62.8	49.8	
Specimen measurements: RMNH DUB 3049, RMNH DUB 3074, RMNH DUB 3072+3097, and RMNH DUB 3112													
***Stegodon orientalis* (×12×)**													
N	4	1	4	4	4	4	4	4	5	8	8	8	
Length	17.4–25.1	20.4	25.5–31.7	25.9–33.8	23.3–34.42	25.5–35.0	28.6–35.3	24.8–39.4	26.8–36.4	25.6–37.0	16.2–31.5	13.4–22.6	
Mean	21.1	–	28.0	28.8	30.3	30.3	31.1	32.3	31.1	29.4	23.0	16.8	
N	4	1	2	3	3	3	4	4	4	6	8	8	
Width	71.6–81.0	75.7	81.2–82.4	68.0–86.1	85.6–88.3	84.9–90.1	72.4–93.1	72.0–91.9	71.2–88.3	64.6–82.7	54.8–78.3	28.7–59.1	
Mean	74.7	–	81.8	79.5	87.1	87.6	84.7	84.2	82.2	75.2	66.0	46.0	
Specimen measurements: IVPP V0577, IVPP V1770, IVPP V1776, IVPP V1817, IVPP V1820, IVPP V1826, IVPP V1827, IVPP V5216-13, and IVPP V5216-15													
	**1^st^**	**2^nd^**	**3^rd^**	**4^th^**	**5^th^**	**6^th^**	**7^th^**	**8^th^**	**9^th^**	**10^th^**	**11^th^**	**12^th^**	**13^th^**
***Stegodon trigonocephalus trigonocephalus* (×13×)**													
N	–	–	–	1	3	3	3	3	3	3	3	3	–
Length	–	–	–	16.8	20.4–25.0	22.1–26.0	24.4–26.0	23.7–24.9	21.4–24.5	21.1–24.0	16.6–24.2	18.4–19.4	–
Mean	–	–	–	–	23.4	24.0	25.3	24.1	22.9	22.5	21.1	19.0	–
N	–	–	–	1	3	3	3	3	3	3	3	3	–
Width	–	–	–	71.8	71.4–87.3	71.4–86.8	75.0–87.1	76.8–83.3	72.6–81.1	70.5–76.4	66.6–69.6	53.9–63.1	–
Mean	–	–	–	–	80.2	80.4	80.8	80.4	77.0	72.6	68.0	58.4	–
Specimen measurements: RMNH DUB 2895, RMNH DUB 3500, and RMNH DUB 4225													
	**1^st^**	**2^nd^**	**3^rd^**	**4^th^**	**5^th^**	**6^th^**	**7^th^**	**8^th^**	**9^th^**	**10^th^**	**11^th^**	**12^th^**	**13^th^**
***Stegodon florensis* (×13×)**													
N	2	2	2	2	2	2	2	1	1	1	1	–	–
Length	23.1–25.8	20.2–20.9	19.2–21.6	18.4–23.8	17.6–22.7	18.9–20.9	18.3–20.3	21.16	21.83	26.48	26.97	–	–
Mean	24.5	20.6	20.4	21.1	20.2	19.9	19.3	–	–	–	–	–	–
N	1	2	2	2	2	2	2	1	1	1	1	–	–
Width	63.1	66.0	67.2–68.4	69.3–69.9	69.0–69.9	67.5–69.9	68.3–68.6	66.95	60.47	65.75	58.71	–	–
Mean	–	66.0	67.8	69.6	69.5	68.7	68.5	–	–	–		–	–
Specimen measurements: RGM.631600													

##### Family ELEPHANTIDAE Gray, 1821

###### Genus *Elephas* Linnaeus, 1758

####### Elephas sp.

Taxon classificationAnimaliaProboscideaElephantidae

######## Referred material.

A fragmentary tusk, DMR-KS-05-03-22-1; a posterior fragment of a right lower molar, DMR-KS-05-03-17-12.

######## Material description.

**Figure 9.**
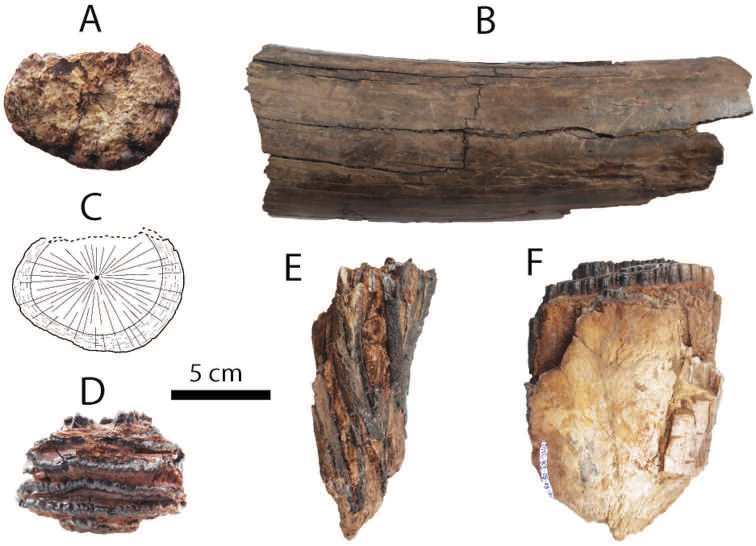
Dental remains of *Elephas* sp. from Khok Sung: **A–C** DMR-KS-05-03-22-1, a fragmentary upper tusk in proximal (**A**, **C**) and ventral (**B**) views **D–F** DMR-KS-05-03-17-12, a posterior fragment of a right lower molar in occlusal (**D**), lingual (**E**), and anterior (**F**) views.

**Upper tusk**: DMR-KS-05-03-22-1 is a short fragmentary tusk. The dorsal side is partially broken away (Fig. 9A, B). This tusk curves slightly upward and is dorsoventrally compressed and probably obovoid or oval in cross-section (Fig. 9B, C). The Schreger pattern in the dentine is poorly developed or absent. The fractures of the cross-section are developed, perpendicular to the outer surface (“radiate cracking or fracture pattern”) (van den Bergh 1999) (Fig. 9C). The maximum length of the preserved tusk is 196.1 mm and the mediolateral and dorsoventral diameters measured on the proximal cross-section are 71.3 and 49.1 mm, respectively.

**Lower molar**: DMR-KS-05-03-17-12 preserves only two adjoining worn plates of a high-crowned molar, distinctly more hypsodont than that of *Stegodon* (Tab. 4). The plates are thin, anteroposteriorly compressed, and closely spaced (Fig. 9D, E). The occlusal enamel loops or folds are small and thin, compared to *Stegodon orientalis* molars, single-layered, and almost irregular. The grinding surface of the anterior plate is buccally inclined (Fig. 9F), indicating this is a right molar.

######## Taxonomic remarks and comparisons.

The fragmentary tusk (DMR-KS-05-03-22-1) is distinguished from DMR-KS-05-03-15-2 (*Stegodon orientalis*) by a more rounded cross-section, a larger diameter, and a radiate fracture pattern with the development of concentric incremental lines (Fig. 9C). The outline of DMR-KS-05-03-22-1 resembles *Elephas* (e.g., *Elephas maximus* (Palombo and Villa 2001) and *Elephas celebensis* (van den Bergh 1999)). The lower molar is also congruent morphologically with *Elephas* (Maglio 1973, Zhou and Zhang 1974), but differs from *Palaeoloxodon namadicus* in its thinner and smoother enamel (Lydekker 1880, Zhou and Zhang 1974, Tshen 2013). We therefore assign these two specimens (fragmentary tusk and molar) to *Elephas*.

#### Order PERISSODACTYLA Owen, 1848

##### Family RHINOCEROTIDAE Owen, 1840

###### Subfamily RHINOCEROTINAE Owen, 1845

####### Genus *Rhinoceros* Linnaeus, 1758

######## Rhinoceros sondaicus

Taxon classificationAnimaliaPerissodactylaRhinocerotidae

Desmarest, 1822

######### Referred material.

A left P2, DMR-KS-05-03-00-128; a left P3, DMR-KS-05-03-22-17; a left M1, DMR-KS-05-03-00-129; a left M3, DMR-KS-05-03-00-127; a mandible with right (i2 and p2–m3) and left (p3–m3) tooth rows, DMR-KS-05-03-00-126; a partial mandible, DMR-KS-05-03-31-28; a fragmentary nasal bone, DMR-KS-05-03-00-56; a left scapula, DMR-KS-05-03-00-58; a left humerus, DMR-KS-05-03-31-3; a right metacarpus II, DMR-KS-05-03-28-29; a metacarpus III, DMR-KS-05-03-22-49; a right metacarpus IV, DMR-KS-05-04-05-15; a left tibia, DMR-KS-05-03-00-52; a right calcaneus, DMR-KS-05-04-27-19; a left astragalus, DMR-KS-05-03-26-23.

######### Material description.

**Figure 10.**
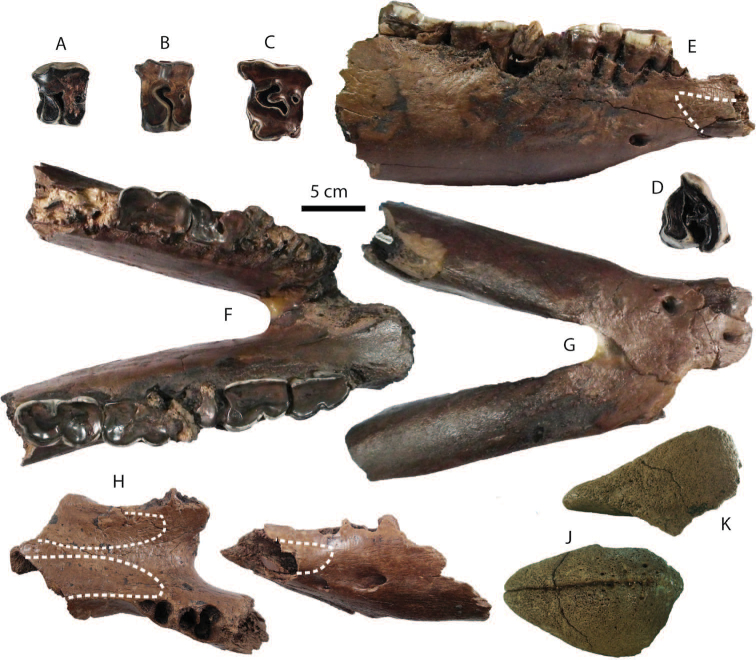
Cranial, mandibular, dental remains of *Rhinoceros sondaicus* from Khok Sung: **A** DMR-05-03-00-128, a left P2 in occlusal view **B** DMR-KS-05-03-22-17, a left P3 in occlusal view **C** DMR-KS-05-03-00-129, a left M1 in occlusal view **D** DMR-KS-05-03-00-127, a left M3 in occlusal view **E–G** DMR-KS-05-03-00-126, a mandible in lateral (**E**), occlusal (**F**) and ventral (**G**) views **H–I** DMR-KS-05-03-31-28, a fragmentary mandible in occlusal (**H**) and lateral (**I**) views **J–K** DMR-KS-05-03-00-56, a nasal in dorsal (**J**) and lateral (**K**) views.

**Upper dentition**: P2 (DMR-KS-05-03-00-128: Fig. 10A), M1 (DMR-KS-05-03-00-129: Fig. 10C), and M3 (DMR-KS-05-03-00-127: Fig. 10D) are presumably from the same individual because they were found together at the same spot. The upper cheek teeth are lophodont (for measurements, see Tab. 9). Premolars are completely molarized (Fig. 10A, B) and molars exhibit well-preserved crochets. The M3 is triangular in occlusal outline and displays a well-developed parastyle, ectometaloph, medifossette, and hypocone, but a less developed parastyle fold (Fig. 10D).

**Table 9.** Measurements (in millimeters) of cheek teeth of Khok Sung rhinoceroses, *Rhinoceros sondaicus* and *Rhinoceros unicornis*, compared to recent specimens (data from Guérin (1980)). “(i)” refers to an isolated tooth and “(m)” indicates a tooth attached to the mandible.

		*Rhinoceros sondaicus*			*Rhinoceros unicornis*		
		Khok Sung		Recent	Khok Sung		Recent
		Anterior	Posterior	Range	Anterior	Posterior	Range
**Upper cheek teeth**							
P2	L	35.57 (i)		30–38.5	–		37–45.5
	W	42.34 (i)	41.24 (i)	34.5–44	–	–	43–48
P3	L	42.00 (i)		36.5–50	–		43–50
	W	55.36 (i)	53.70 (i)	42–55	–	–	55.5–60.5
P4	L	–		41–47.5	–		42–51
	W	–	–	52–59	–	–	59–69.5
M1	L	51.38 (i)		46–51	47.95 (i)		48–58
	W	63.53 (i)	58.67 (i)	52.5–60	70.48 (i)	58.80 (i)	62–72.5
M2	L	–		44.5–55	–		53–62
	W	–	–	53–62.5	–	–	64.5–76
M3	L	55.65 (i)		44.5–61.5	–		59–65
	W	55.92 (i)		43.5–57	–	–	56–68.5
**Lower cheek teeth**							
p2	L	–		25–29.5	> 30.80 (i)		31–32
	W	–	–	15.5–21	18.15 (i)	22.39 (i)	21.5–24.5
p3	L	42.83 (m)		33–39	40.24 (m)		38–42
	W	26.58 (m)	29.92 (m)	22–27.5	–	–	27–32
p4	L	43.03 (m)		36.5–42.5	48.13 (m)		41–46
	W	27.71 (m)	33.42 (m)	24–29	–	–	29–34
m1	L	41.45 (m)		41–46.5	42.57 (m)		46–48
	W	28.8–29.67 (m)	30.88 (m)	26–32	–	–	28–32.5
m2	L	44.83–48.87 (m)		40.5–51	50.74 (m)		52–56.5
	W	29.65 (m)	30.78–31.79 (m)	27–32.5	–	–	31–36
m3	L	54.90 (m)		41–53	55.48 (m)		49.5–60
	W	32.54 (m)	25.11* (m)	24.5–29.5	–	–	29–35
**Lower tooth rows**							
		**DMR–KS–05–03–00–126**		**Recent**	**DMR-KS–05–03–17–13**		**Recent**
Molar row length		133 (right)		126.5–147	158		147.5–161
Tooth row length		> 238		211.5–257	–		242–276

**Table 10.** Measurements (lengths and widths in millimeters) of cheek teeth of Khok Sung *Sus barbatus* compared to the recent and fossil species. The number of specimens is given within the parentheses. The measured specimens of recent *Sus scrofa* include three subspecies: *Sus scrofa scrofa*, *Sus scrofa vittatus*, and *Sus scrofa attila*.

		Khok Sung	Recent				Java (Pleistocene)	
		*Sus barbatus*	*Sus scrofa*	*Sus barbatus*	*Sus verrucosus*	*Sus celebensis*	*Sus brachygnathus*	*Sus macrognathus*
P3	L	16.75	12.33–14.41 (16)	13.17–14.98 (12)	11.87–13.77 (8)	9.37	11.09–12.29 (7)	12.47–13.86 (2)
	W	14.42	10.12–12.19 (16)	10.06–13.22 (12)	9.71–12.64 (8)	7.35	9.60–11.54 (7)	10.75–13.43 (2)
P4	L	15.06	11.41–14.61 (16)	12.56–14.81 (12)	11.85–13.97 (8)	8.96–9.44 (3)	10.35–11.61 (7)	11.89–12.39 (3)
	W	18.59	12.77–15.23 (16)	13.51–16.00 (12)	13.23–14.82 (8)	10.68–11.01 (3)	11.05–13.80 (7)	13.72–15.68 (3)
M1	L	20.68	14.01–17.88 (16)	16.71–19.24 (12)	14.36–16.13 (8)	13.44–13.76 (3)	13.89–14.94 (6)	13.62–17.17 (3)
	W	17.17	13.59–17.57 (16)	13.59–15.85 (12)	13.32–15.78 (8)	10.59–11.49 (3)	12.68–14.36 (6)	12.57–15.54 (3)
M2	L	29.35–29.49 (2)	20.08–24.78 (16)	22.60–24.60 (12)	20.53–22.39 (8)	16.89–17.98 (4)	19.81–24.26 (7)	17.17–24.38 (4)
	W	21.37–23.40 (3)	16.43–20.82 (16)	17.45–19.89 (12)	16.82–19.74 (8)	13.33–14.96 (4)	16.09–17.97 (7)	15.54–21.06 (4)
M3	L	37.36	29.09–39.01 (16)	30.31–36.50 (12)	31.75–37.13 (8)	21.59–24.81 (3)	27.27–33.26 (8)	31.44–40.89 (60
	W	21.46–24.97 (2)	19.68–23.76 (16)	17.44–24.94 (12)	18.73–20.59 (8)	14.88–16.18 (3)	18.08–20.37 (8)	19.95–24.30 (6)
p1	L	7.32–7.71 (2)	7.03–9.13 (8)	7.25–9.51 (8)	5.42–7.81 (3)	?	6.32–9.98 (6)	?
	W	4.16–4.35 (2)	3.56–4.17 (8)	3.33–4.09 (8)	3.22–3.88 (3)	?	3.50–5.15 (6)	?
p2	L	11.71–13.17 (4)	10.42–13.21 (16)	12.10–14.80 (12)	10.77–11.89 (8)	?	9.96–12.02 (6)	?
	W	5.55–6.66 (4)	4.48–6.49 (16)	4.78–6.61 (12)	5.84–6.43 (8)	?	4.87–5.46 (6)	?
p3	L	13.17–14.31 (4)	13.09–15.75 (16)	14.01–16.07 (12)	12.91–14.85 (8)	10.31	11.94–14.59 (7)	12.14–13.84 (2)
	W	7.84–8.60 (4)	6.32–9.10 (16)	6.51–8.53 (12)	6.49–7.80 (8)	6.57	6.56–7.38 (7)	7.44–7.46 (2)
p4	L	13.87–15.01 (3)	13.40–16.05 (16)	14.57–17.29 (12)	14.44–16.10 (8)	10.11–10.22 (2)	12.75–14.30 (8)	15.41–15.75 (2)
	W	10.13–11.68 (3)	8.78–11.44 (16)	9.18–10.60 (12)	8.79–11.28 (8)	7.46–8.34 (2)	8.84–10.60 (8)	9.56–10.48 (2)
m1	L	14.32–18.47 (2)	14.64–18.75 (16)	15.94–19.60 (12)	12.90–14.95 (8)	12.34–12.61 (3)	13.77–14.83 (8)	15.81–17.94 (2)
	W	13.11–13.8 (2)	11.55–13.94 (16)	10.84–13.22 (12)	11.04–13.56 (8)	8.55–9.92 (3)	10.80–12.07 (8)	11.79–12.11 (2)
m2	L	19.96–23.38 (2)	19.66–24.24 (16)	21.84–23.97 (12)	19.88–21.22 (8)	15.35–16.01 (4)	17.19–20.84 (8)	21.31–25.00 (3)
	W	17.65–18.06 (2)	14.61–17.39 (16)	14.61–16.56 (12)	14.14–15.95 (8)	10.77–13.25 (4)	12.96–14.45 (8)	14.15–16.30 (3)
m3	L	40.92	32.92–41.27 (16)	35.60–43.02 (12)	37.45–40.27 (8)	21.68–24.44 (3)	30.56–39.84 (7)	40.72–46.37 (4)
	W	19.89	16.71–19.32 (16)	16.24–19.74 (12)	15.92–17.84 (8)	12.16–13.38 (3)	16.06–21.44 (7)	15.84–18.15 (4)

**Mandibles and lower dentition**: a mandible (DMR-KS-05-03-00-126) preserves both sides of cheek tooth rows (right p2–m3 and left p3–m3), but most of its symphysis and entire ramus are broken off (Fig. 10E–G) (for measurements, see Appendix 2). The posterior edge of the mandibular symphysis ends nearly at the middle part of p3. The ventral margin of the mandible is convex in lateral view (Fig. 10E). The mental foramen is situated below the p3. In ventral view, the small foramen is present at the central portion of the mandibular symphysis and the lingual mandibular outline is U-shaped (Fig. 10F, G). Only the basal part of a right tusk-like incisor is preserved in its socket. Another specimen DMR-KS-05-03-31-28 preserves a nearly complete mandibular symphysis and left p2 and p3 sockets (Fig. 10H, I). The left mandibular body behind the p3 is broken away. All lower cheek teeth are heavily worn and rectangular in occlusal outline (Fig. 10F) (for measurements, see Tab. 9).

**Nasal**: a nasal bone (DMR-KS-05-03-00-56) is short and robust, bending downward and narrowing anteriorly towards the tip (Fig. 10J). The anterior surface is nearly straight in lateral view (Fig. 10K), whereas its ventral surface is flattened at the central suture. This nasal bone is most similar to *Rhinoceros sondaicus* (e.g., specimen MNHN-ZMO-1985-159), because its anterior part is pointed rather than rounded (Colbert 1942). In comparison, *Rhinoceros unicornis* displays a convex anterior surface in lateral view and a well-developed horn protuberance of the nasal region. The maximum length and width of the nasal are 131.1 mm and 88.8 mm, respectively.

**Figure 11.**
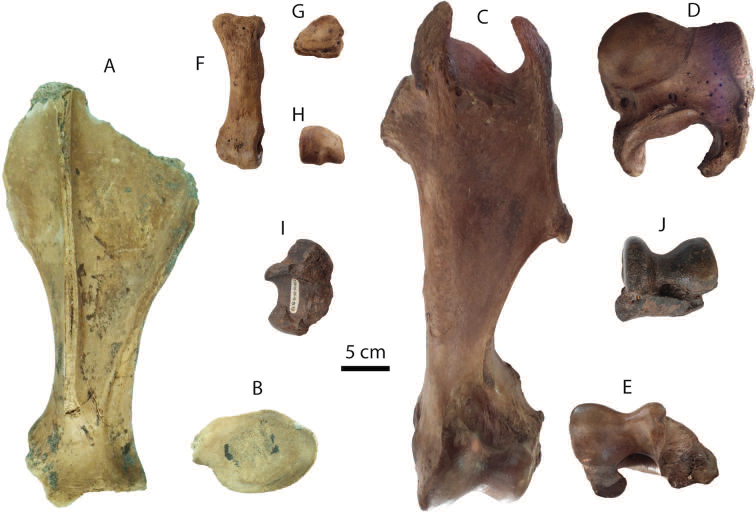
Postcranial remains of *Rhinoceros sondaicus* from Khok Sung: **A–B** DMR-KS-05-03-00-58, a left scapula in lateral (**A**) and distal (**B**) views **C–E** DMR-KS-05-03-31-3, a left humerus in anterior (**C**), proximal (**D**), and distal (**E**) views **F–H** DMR-KS-05-04-05-15, a right metacarpus IV in posterior (**F**), proximal (**G**), and distal (**H**) views **I** DMR-KS-05-04-27-19, a right calcaneus in lateral view **J** DMR-KS-05-03-26-23, a left astragalus in dorsal view.

**Postcranial remains**: postcranial elements include a scapula (Fig. 11A, B), a humerus (Fig. 11C–E), three metacarpal bones (metacarpus II, III, and IV: Fig. 11F–H), a tibia, a calcaneus (Fig. 11I), and an astragalus (Fig. 11J). All postcranial remains are comparable in size to the recent material (Guérin 1980) (for measurements, see Appendix 1).

######### Taxonomic remarks and comparisons.

Four isolated cheek teeth (P2, P3, M1, and M3) assigned to *Rhinoceros sondaicus* are characterized by the following morphological features: a presence of the moderately developed crochet, sinuosity of the ectoloph, distinct parastyle fold, and deeper median valley compared to the posterior valley, and the absences of an antecrochet, protocone fold, and metacone bulge on M3. All of these characters coincide with the upper molars of *Rhinoceros sondaicus* (Pocock 1945, Hooijer 1946, Zin-Maung-Maung-Thein et al. 2006, Groves and Leslie 2011).

Large tusk-like incisors (i2) are notably typical of Asian rhinoceroses. The two small alveoli corresponding to the lost central incisors are autapomorphic of *Rhinoceros* (Groves and Leslie 2011). Our observations on the recent mandible iPHEP M05.5.001.B and MNHN-ZMO-1985-159 demonstrate that an alveolus extension of the lower incisors that reach posteriorly to the lingual side of the p2 is a characteristic of both living Javan (*Rhinoceros sondaicus*) and Indian (*Rhinoceros unicornis*) rhinoceroses (Tong and Guérin 2009). This feature efficiently distinguishes *Rhinoceros* from the Sumatran rhinoceros, *Dicerorhinus sumatrensis*, where the alveoli of the lower incisors do not extend as far (Tong and Guérin 2009). In the mandibles DMR-KS-05-03-00-126 and DMR-KS-05-03-31-28, the lower incisor alveoli extend posteriorly into the mandibular symphysis, ventral to the lingual side of the p2 (Fig. 11H, I). The latter specimen also shares similar mandibular dimensions (Appendix 2) and morphology with the former specimen.

Isolated lower molars of rhinoceroses from Khok Sung are difficult to assign to either *Rhinoceros unicornis* or *Rhinoceros sondaicus* due to heavy wear. In addition, there is a significant size overlap between these two species (Guérin 1980). The lengths of lower cheek teeth and molar rows provide a better distinction (little overlap in size) than those of isolated teeth. The lengths and widths of the cheek teeth on the mandible DMR-KS-05-03-00-126 fall almost within the range of *Rhinoceros sondaicus*, with the exception of some specimens (p3, p4, and m3) that fit well with the larger-sized *Rhinoceros unicornis* (Tab. 9). However, the lengths of the mandibular cheek tooth and molar rows of this specimen fall within the ranges of *Rhinoceros sondaicus* (211.5–257 mm and 126.5–147 mm, respectively) and outside of the ranges for *Rhinoceros unicornis* (Guérin 1980: table. 6). The two mandibles, DMR-KS-05-03-00-126 and DMR-KS-05-03-31-28, are thus assigned to *Rhinoceros sondaicus*.

######## Rhinoceros unicornis

Taxon classificationAnimaliaPerissodactylaRhinocerotidae

Linnaeus, 1758

######### Referred material.

A left mandible with p3–m3, DMR-KS-05-03-17-13; a left p2, DMR-KS-05-03-19-4; a right M1, KS-05-03-18-X; a left femur, DMR-KS-05-03-00-63; a left astragalus, DMR-KS-05-03-00-67.

######### Material description.

**Figure 12.**
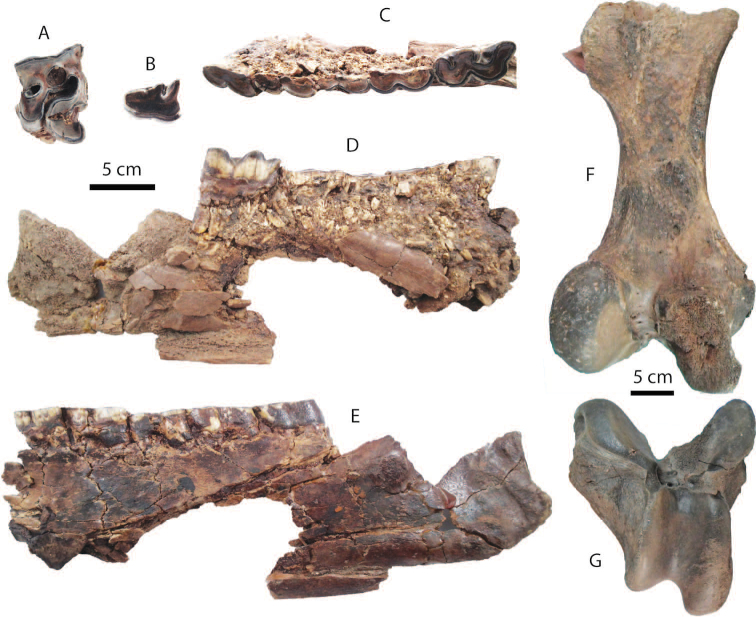
Remains of *Rhinoceros unicornis* from Khok Sung: **A** DMR-KS-05-03-18-X, a right M1 in occlusal view **B** DMR-KS-05-03-19-4, a left p2 in occlusal view **C–E** DMR-KS-05-03-17-13, a left mandible in occlusal (**C**), medial (**D**), and lateral (**E**) views **F–G** DMR-KS-05-03-00-63, a left femur in posterior (**F**) and distal (**G**) views.

**Upper dentition**: a relatively worn M1 (DMR-KS-05-03-18-X) is nearly square in outline and displays a flattened ectoloph and a well developed crochet, medifossette, and posterior fossette (Fig. 12A) (for measurements, see Tab. 9).

**Mandible and lower dentition**: a hemi-mandible (DMR-KS-05-03-17-13) is strongly compressed laterally and preserves a partial mandibular ramus and body with worn cheek teeth, except for the m3 which is unbroken (Fig. 12C–E) (for measurements, see Appendix 2). The lingual portion along the mandible is entirely broken. The mandibular depth below the m3 is higher than that of *Rhinoceros sondaicus*. An isolated p2 is relatively worn and broken at its posterior part (Fig. 12B). At the lingual side of the p2, the anterior valley is slightly developed, whereas the posterior valley is prominent.

**Postcranial remains**: an isolated femur (Fig. 12F, G) and astragalus are comparable in size to *Rhinoceros unicornis*, but are larger than *Rhinoceros sondaicus* (Guérin 1980) (for measurements, see Appendix 1).

######### Taxonomic remarks and comparisons.

We assign the M1 (DMR-KS-05-03-18-X) to *Rhinoceros unicornis* according to the presence of the flattened ectoloph and enclosed medifossette (on a worn specimen), as well as its larger size than that of *Rhinoceros sondaicus*. These upper molar features are characteristic of *Rhinoceros unicornis* (Colbert 1942). For the lower dentition, the size of the isolated p2 (DMR-KS-05-03-19-4) and the molar row length of the mandible DMR-KS-05-03-17-13 (Tab. 9) are comparable to those of recent *Rhinoceros unicornis* (31–32 mm and 147.5–161 mm, respectively) (Guérin 1980: table. 6). Therefore, another species of rhinoceroses, *Rhinoceros unicornis*, is identified at Khok Sung.

#### Order ARTIODACTYLA Owen, 1848

##### Family SUIDAE Gray, 1821

###### Genus *Sus* Linnaeus, 1758

####### Sus barbatus

Taxon classificationAnimaliaArtiodactylaSuidae

Müller, 1838

######## Referred material.

A left maxillary fragment with P3–M2, DMR-KS-05-04-19-2; two left M2—DMR-KS-05-04-19-5 and DMR-KS-05-03-18-23 (posterior portion); two right M3—DMR-KS-05-04-03-4 and DMR-KS-05-04-19-4 (anterior portion); two mandible with two tooth rows—DMR-KS-05-03-15-1 (right: i1, i2, c1, p2, and p3 and left: i1, i2, c1, and p2–m2) and DMR-KS-05-04-19-1 (right: i1, i2, c1, and p1–m3 and left: i1, i2, c1, and p1–p4); a left posterior fragment of m3, DMR-KS-05-04-19-3; a right humerus, DMR-KS-05-03-26-8.

######## Material description.

**Figure 13.**
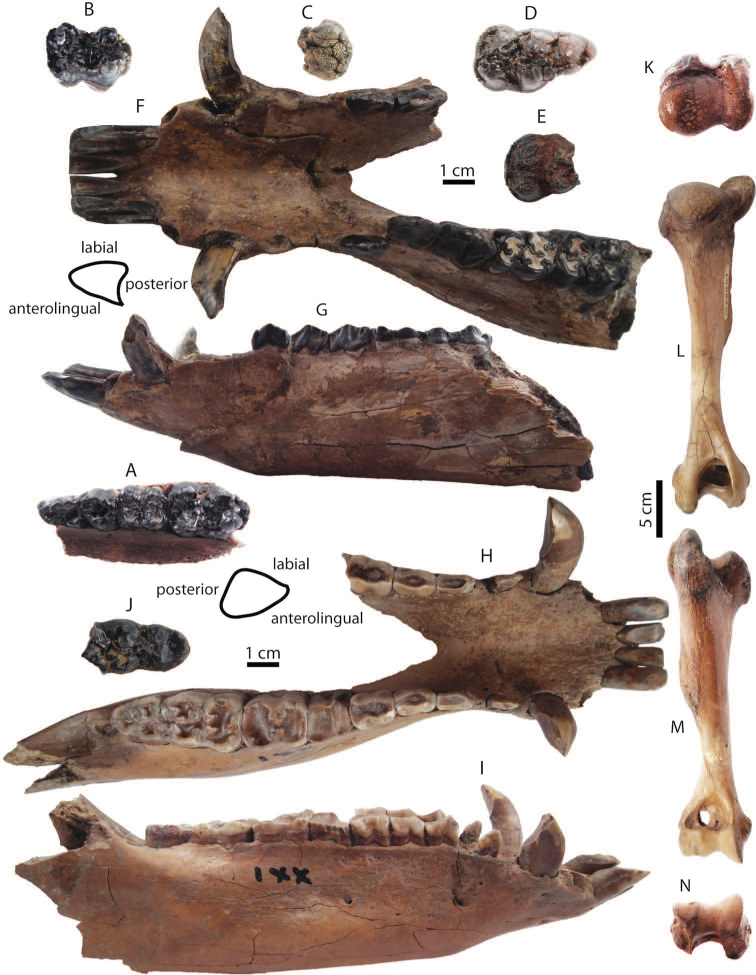
Remains of *Sus barbatus* from Khok Sung: **A** DMR-KS-05-04-19-2, a left upper cheek tooth row in occlusal view **B** DMR-KS-05-04-19-5, a left M2 **C** DMR-KS-05-03-18-23, a left fragmentary M2 **D** DMR-KS-05-04-03-4, a right M3; (**E**) DMR-KS-05-04-19-4, a right M3 **F–G** DMR-KS-05-03-15-1, a mandible in occlusal (**F**) and lateral (**G**) views **H–I** DMR-KS-05-04-19-1, a mandible in occlusal (**H**) and lateral (**I**) views **J** DMR-KS-05-04-19-3, a left fragmentary m3 **K–N** DMR-KS-05-03-26-8, a right humerus in proximal (**K**), posterior (**L**), anterior (**M**), and distal (**N**) views. Cross-sections of canines are given. All isolated teeth are shown in occlusal view.

**Upper dentition**: DMR-KS-05-04-19-2 is a maxillary tooth row preserving a slightly worn P3 to M2 (Fig. 13A). The P3 and P4 show *Sus*-like patterns with distinctly pre- and poststyles on the buccal side. On the P3, the paracone is well-developed and the postcrista projects posterobuccally. On the P4, three main cusps (protocone, paracone, and metacone) are distinct and the protofossa is present. Upper molars are unworn to slightly worn and exhibit distinct main (protocone, paracone, metacone, tetracone, and pentacone) and accessory (tetrapreconule, pentapreconule, and ectoconule) cusps. The posterior cingulum on the M2 is more developed than on the M1 (Fig. 13A–C). The M3 (DMR-KS-05-04-03-4: Fig. 13D) is unworn and subtriangular in outline and has a distinct anterior cingulum, pentacone, and pentapreconule and bulky accessory cusps. Another M3 (DMR-KS-05-04-19-4) does not preserve a posterior part but has well-developed main cusps, anterior cingulum, median valley, tetrapreconule, and ectoconule (Fig. 13E). The cheek teeth of DMR-KS-05-04-19-4 are larger than those of DMR-KS-05-04-03-4.

**Mandible and lower dentition**: DMR-KS-05-03-15-1 is incomplete, lacking the body and ascending ramus, broken posterior to the right p3 and to the left m2 (Fig. 13F, G) (for measurements, see Appendix 3). The mandible is inflated. The small mental foramen is present below the diastema between p1 and p2. Only the i3 and p1 are missing. The left p2 is not aligned along the cheek tooth row due to the deformation. The specimen DMR-KS-05-04-19-1 preserves a complete symphysis and a right body with the tooth row. The ramus is broken away (Fig. 13H, I). The mandibular body is successively inflated. The mental foramina are situated below the diastema between p1 and p2. For the specimen DMR-KS-05-04-19-1, the teeth are complete and moderately to heavily worn but the third incisors are missing.

Lower incisors show a chisel-like appearance with long roots. The i2 is larger than the i1. Lower canines are slender and pointed, and curve backward. The lower canines of the mandible DMR-KS-05-03-15-1 belong to a male individual because of a more sharply triangular section (Hillson 2005) (Fig. 13F). The mandible DMR-KS-05-04-19-1 possesses a female canine characterized by more rounded cross-sections and well-developed roots (Hillson 2005) (Fig. 13H). The lower canines of the male specimen are more laterally inclined (about 30° from the cheek teeth) than those of the female individual (about 15°). The cross-section outlines of male canines (DMR-KS-05-03-15-1) are of the “verrucosic” type in which the posterior side is narrower than the labial one (Fig. 13F). All lower cheek teeth exhibit bunodont patterns with accessory tubercles, like in *Sus*. The lower cheek teeth increase in size from anteriorly to posteriorly (Tab. 10). Lower premolars are slightly to moderately worn. The p1 is unicuspid. Other premolars are tricuspid. All cuspids are sharp. The highest cuspid on the premolars is the metaconid. Lower molars are moderately to heavily worn and rectangular in outline (Fig. 13F–J). The lower molars show complex occlusal patterns with well-developed main cuspids (protoconid, metaconid, hypoconid, entoconid, and pentaconid) and a bulky median column (hypopreconulid). The m2 is much larger and has a more developed posterior cingulid than the m1 (Fig. 13F, H). The m3 (DMR-KS-05-04-19-1) is elongated posteriorly (Fig. 13H). It has a well-developed talonid with bulky main and accessory cuspids (pentaconid, pentapreconulid, hexaconid, heptaconid). Another isolated posterior fragment (talonid) of the m3 (DMR-KS-05-04-19-3) is also elongated, as long as that of DMR-KS-05-04-19-1. This specimen exhibits smooth occlusal surfaces with wear and well developed main and accessory cuspids (Fig. 13J). The m3 is longer than the combination of m1 and m2 (Tab. 10).

**Postcranial bone**: DMR-KS-05-03-26-8 is a complete humerus (Fig. 13K–N), characterized by its prominent tubercle slightly overhanging the large bicipital groove (Fig. 13K), proximal part becoming wider than long (Fig. 13K), mesially flat and laterally compressed shaft, distinct deltoid ridge starting at the mid-shaft (Fig. 13L, M), large supinator ridge and supratrochlear foramen (Fig. 13M), shallow musculo-spiral groove (Fig. 13N), and small deltoid tuberosity (Fig. 13N). The size and morphology of the humerus DMR-KS-05-03-26-8 resemble those of recent *Sus barbatus* (for measurements, see Appendix 1).

######## Taxonomic remarks and comparisons.

We compare our material to some Pleistocene Southeast Asian suid species, although only two distinct suid species, *Sus scrofa* and *Sus barbatus*, are known from many Pleistocene localities of mainland Southeast Asia. The sizes of the Khok Sung material are obviously larger than those of Pleistocene and extant Indonesian suids (*Sus brachygnathus*, *Sus macrognathus*, *Sus verrucosus*, and *Sus celebensis*) (Tab. 10). The Khok Sung suid material is comparable in size to *Sus scrofa* and *Sus barbatus*. The two suid mandibles from Khok Sung also show some distinctive taxonomic characters of *Sus scrofa* and *Sus barbatus*. For example, the mandible is not laterally enlarged or swollen and the diastema from p1 to p2 is longer than from c1 to p1, which are only characteristics of some species of *Sus*: *Sus scrofa*, *Sus celebensis*, and *Sus barbatus* (Groves 1997). The lower premolar rows on the mandibles are aligned along the mandible, unlike *Sus verrucosus* and *Sus celebensis* in which the premolar rows diverge anteriorly (Groves 1997).

However, it is difficult to distinguish *Sus scrofa* from *Sus barbatus* only based on the cheek teeth because both species overlap in size (Tab. 10) and show almost similar dental patterns. The main differential characters between *Sus scrofa* and *Sus barbatus* are defined on the basis of the shape of lower canines in male individuals, whether the outline of the cross-section is of the “scrofic” (i.e. the posterior side is wider than the labial one (*Sus scrofa*)) or “verrucosic” (*Sus barbatus*) type (Badoux 1959, Hardjasasmita 1987). Similarly, this distinctive feature is demonstrated by the lower male canine index (the width of labial surface as a percentage of the width of posterior surface) (Groves 1981, 1997). The canine index ranges from 61.5 to 109.1 for recent *Sus scrofa* and from 105.6 to 144.4 for extant *Sus barbatus* (Groves 1981: table. 1). The lower canines of the male mandible DMR-KS-05-03-15-1 show the verrucosic type with the canine index of *Sus barbatus* (for the detailed calculation see Tab. 11). We also provide the canine index of the female specimen DMR-KS-05-04-19-1 in Tab. 11. A minor distinctive character between *Sus scrofa* and *Sus barbatus* is differences of the posterior accessory median cuspid (pentapreconulid) on the talonid. The pentapreconulid on the m3 is small or absent in *Sus barbatus* (Badoux 1959). For other molar characters, *Sus barbatus* shows more complex patterns with accessory tubercles and more rugose enamel than in *Sus scrofa* (Tougard 1998, Bacon et al. 2011). However, the latter character is useless to make a distinction between both suid species according to our observations on the recent material of *Sus barbatus*. The enamel surfaces of the molars in *Sus barbatus* are often smooth or even sometimes smoother than in *Sus scrofa*.

**Table 11.** Measurements (in millimeters) of lower canines of Khok Sung *Sus barbatus*. The canine index is expressed by the following formula: labial surface*100/posterior surface (Groves 1981).

Specimen no.		Widths			Canine index
		anterolingual surface	posterior surface	labial surface	
DMR-KS-05-03-15-1 (male)	right c1	13.61	8.54	11.46	134.2
	left c1	13.88	9.05	11.57	127.8
DMR-KS-05-04-19-1 (female)	right c1	13.18	11.92	10.32	86.6
	left c1	13.30	12.21	10.54	86.3

The female mandible (DMR-KS-05-04-19-1) and other isolated teeth are assigned to *Sus barbatus* according to those described features. We also suggest that Pleistocene *Sus barbatus* probably shows evidence of sexual size dimorphism because the female specimen DMR-KS-05-04-19-1 is markedly smaller than the male specimen DMR-KS-05-03-15-1, as seen in the recent population.

##### Family CERVIDAE Gray, 1821

###### Genus *Axis* Hamilton-Smith, 1827

####### Axis axis

Taxon classificationAnimaliaArtiodactylaCervidae

(Erxleben, 1777)

######## Referred material.

Four crania—DMR-KS-05-04-18-50 (with two antlers), DMR-KS-05-03-00-30 (with left partial and right broken antlers), DMR-KS-05-03-18-X9 (with pedicles), and DMR-KS-05-03-27-1 (with pedicles); two right complete antlers—DMR-KS-05-03-31-30 and DMR-KS-05-03-22-4; a nearly complete left antler, DMR-KS-05-04-4-1; five right fragmentary antlers—DMR-KS-05-03-18-21, DMR-KS-05-03-19-82, DMR-KS-05-03-28-22, DMR-KS-05-06-22-2, and DMR-KS-05-03-28-1; eight left fragmentary antlers—DMR-KS-05-03-00-12, DMR-KS-05-03-19-81, DMR-KS-05-03-22-2, DMR-KS-05-03-24-1, DMR-KS-05-04-09-1, DMR-KS-05-03-19-13, DMR-KS-05-03-26-21, and DMR-KS-05-03-08-17; two left fragmentary maxilla—DMR-KS-05-03-28-6 (with M1–M3) and DMR-KS-05-03-08-31 (with P3, P4, and M1 root); a right P4, DMR-KS-05-04-01-3; a left M1, DMR-KS-05-04-28-5; a left M2, DMR-KS-05-03-14-5; thirteen right mandibles—DMR-KS-05-03-14-2 (with m3), DMR-KS-05-03-20-1 (with p4–m3), DMR-KS-05-03-20-2 (with m2 and m3), DMR-KS-05-03-22-7 (with m2 and m3), DMR-KS-05-04-03-1 (with p2–m3), and DMR-KS-05-03-27-3 (with m2 and m3), DMR-KS-05-03-19-1 (with p2–m3), DMR-KS-05-03-22-8 (with m2 and m3), DMR-KS-05-04-01-1 (with p2–m3), DMR-KS-05-03-24-4 (with m2), DMR-KS-05-03-26-12 (with m2 and m3), DMR-KS-05-04-7-10 (with p3, m1, and m2), and DMR-KS-05-03-26-10 (with p2–m1); eight left mandibles—DMR-KS-05-03-18-22 (with p2), DMR-KS-05-03-22-6 (with m1–m3), DMR-KS-05-03-27-22 (with p3-m2 sockets and broken m3), DMR-KS-05-04-09-2 (with p3, p4, m1 and m2 sockets, and m3), DMR-KS-05-03-00-102 (with p4 and m1), DMR-KS-05-03-19-2 (with m1–m3), DMR-KS-05-03-23-1 (with p2 and p3 roots and p4–m3), and DMR-KS-05-03-29-1 (with p2-m3); a left m1, DMR-KS-05-04-28-6; three m2—DMR-KS-05-03-25-4 (right), DMR-KS-05-03-00-104 (left), and DMR-KS-05-03-22-11 (left); four left m3—DMR-KS-05-04-9-4, DMR-KS-05-03-22-9, DMR-KS-05-04-01-2, and DMR-KS-05-03-08-33; three right fragmentary humeri (distal part)—DMR-KS-05-03-13-4, DMR-KS-05-04-11-32, and DMR-KS-05-03-17-17; six metacarpi—DMR-KS-05-03-18-2 (right), DMR-KS-05-03-19-3 (right), DMR-KS-05-03-22-28 (right), DMR-KS-05-03-08-2 (right), DMR-KS-05-04-30-20 (right proximal fragment), and DMR-KS-05-03-19-37 (left); a right fragmentary femur, DMR-KS-05-03-27-4 (distal part); three metatarsi—DMR-KS-05-03-26-3 (right), DMR-KS-05-03-29-30 (left), and DMR-KS-05-03-15-14 (left).

######## Material description.

**Figure 14.**
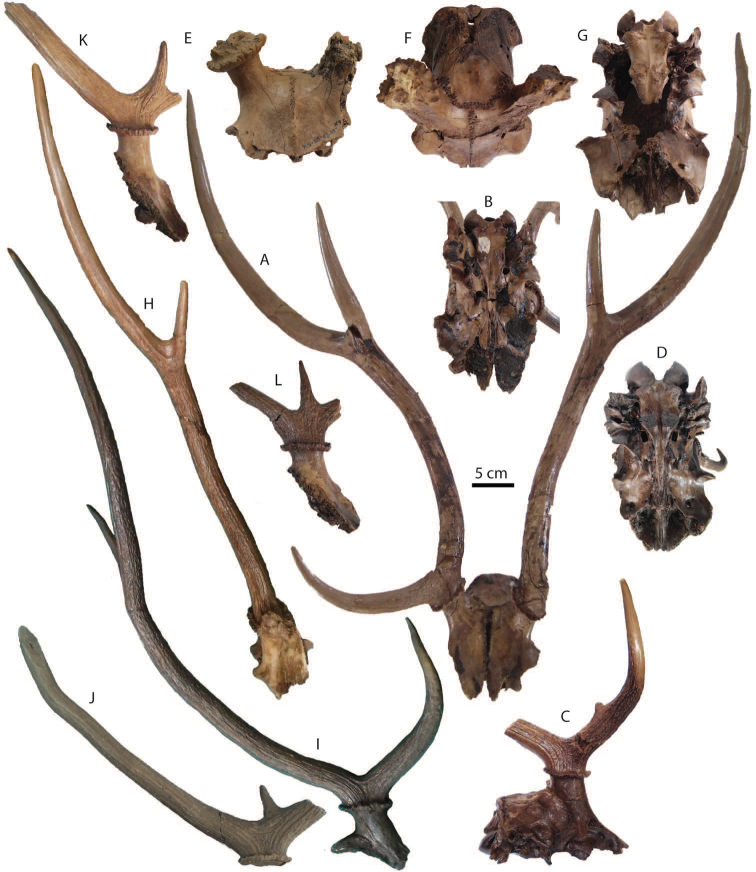
Cranial remains of *Axis axis* from Khok Sung: **A–B** DMR-KS-05-04-18-50, a cranium with nearly complete antlers in dorsal (**A**) and ventral (**B**) views **C–D** DMR-KS-05-03-00-30, a cranium in lateral (**C**) amd ventral (**D**) views **E** DMR-KS-05-03-18-X9, a cranium in anterior view **F–G** DMR-KS-05-03-27-1 a cranium in dorsal (**F**) and ventral (**G**) views **H** DMR-KS-05-03-31-30, a right antler in anterior view; (**I**) DMR-KS-05-03-22-4, a right antler in lateral view **J** DMR-KS-05-03-18-21, a left antler fragment in lateral view **K** DMR-05-03-22-2, a left antler fragment in lateral view **L** DMR-KS-05-03-19-81, a left antler fragment in medial view.

**Crania and upper dentition**: four crania are almost complete, lacking only the anterior portions (e.g., nasal, jugal, palatine, and maxilla) (Fig. 14A–D). The specimen DMR-KS-05-04-18-50 shows nearly complete antlers, lacking only the left brow tine (Fig. 14A, B). The cranium DMR-KS-05-03-00-30 possesses a right antler portion preserving the complete brow tine but the broken main beam (Fig. 14C, D). The specimens DMR-KS-05-03-18-X9 (Fig. 14E) and DMR-KS-05-03-27-1 (Fig. 14F, G) preserve most of the rear part of the skull but lacks zygomatic arcs and antler portions. The specimen DMR-KS-05-03-27-1 preserves a deformed frontal area and broken pedicles (Fig. 14F). The basioccipital and basisphenoid are subtriangular in ventral view and show well-deveoped anterior and posterior tuberosities with a longitudinal groove running along the central part (Fig. 14B, D, G). The lateral edges of the basioccipital and basisphenoid are concave like in *Axis*. The foramina ovale are large and open ventrolaterally. The shed antlers are characterized by three main tines, smooth surfaces, a short pedicle and brow tine, a long and slender main beam, a high angle (about 100-120°) between the main beam and the brow tine, and a well-developed burr (Fig. 14A, C, H–L). A small ornamented tine (or knob) is sometimes present along the dorsal surface of the brow tine or at the main beam-brow tine junction (Fig. 14C, J–L). The main beam is oriented upward, laterally, and posteriorly, and consists of forked tines apically. At the antlered crown, the inner tine is much shorter than the outer one (Fig. 14A, H, I). The skull and antler exhibit a typical arrangement of recent *Axis axis* (e.g., the orientation of the main beam and brow tine, the bifurcation at the apical crown tine, and the shape of the basioccipital and basisphenoid) (for measurements, see Appendix 4).

**Figure 15.**
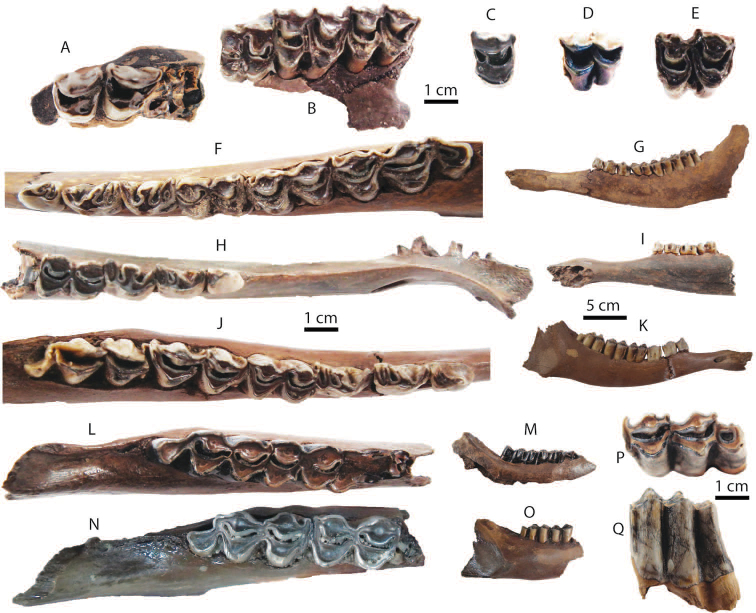
Dental remains of *Axis axis* from Khok Sung: **A** DMR-KS-05-03-08-31, an upper left P3 and P4 in occlusal view **B** DMR-KS-05-03-28-6, a left upper molar row in occlusal view **C** DMR-KS-05-04-01-3, a right P4 in occlusal view **D** DMR-KS-05-04-28-5, a left M1 in occlusal view **E** DMR-KS-05-03-14-5, a left M2 in occlusal view **F–G** DMR-KS-05-03-29-1, a left mandible in occlusal (**F**) and lateral (**G**) views **H–I** DMR-KS-05-03-26-10, a right mandibular fragment in occlusal (**H**) and medial (**I**) views **J–K** DMR-KS-05-04-03-1, a right mandible in occlusal (**J**) and lateral (**K**) views **L–M** DMR-KS-05-03-20-1, a right mandible in occlusal (**L**) and lateral (**M**) views **N–O** DMR-KS-05-03-22-7, a right mandible in occlusal (**N**) and lateral (**O**) views **P–Q** DMR-KS-05-03-08-33, a left m3 in occlusal (**P**) and buccal (**Q**) views.

P3 and P4 are similar to recent *Axis*, characterized by well-developed styles, medial cristae (more distinct on the P4), and posterolingual fossettes (Fig. 15A) (for measurements, see Tab. 12). On the P4, the medial cristae join the postmetacrista and divide the fossa into two islands (Fig. 15A, C). Upper molars display distinct styles (particularly the mesostyle), entostyles, and anterior cingula (Fig. 15B, D, E). The metaconule fold is slightly developed. The M2 is slightly wider than the M3 (Tab. 12). The posterior lobe of the M3 is reduced in width (Fig. 15B).

**Table 12.** Measurements (lengths and widths in millimeters) of cervid teeth from Khok Sung. N=number of specimens.

	Length			Width		
	N	Range	Mean	N	Range	Mean
***Axis axis***						
P3	1	12.40	–	1	13.60	–
P4	2	10.04–11.29	10.67	2	12.19–14.28	13.24
M1	2	13.32–15.19	14.26	2	15.60–15.93	15.77
M2	2	18.07–18.08	18.08	2	17.41–17.84	17.63
M3	1	17.53	–	1	16.42	–
p2	6	7.93–9.54	8.72	6	5.44–6.89	5.93
p3	7	9.17–12.11	10.67	7	6.53–7.14	6.88
p4	8	10.64–13.62	11.65	10	6.77–8.13	7.39
m1	9	11.81–18.20	14.2	13	8.27–10.29	9.59
m2	18	15.94–21.42	17.91	19	8.56–11.67	10.56
m3	18	21.69–25.78	24.1	20	8.87–11.89	10.74
***Panolia eldii***						
P2	1	11.09	–	1	13.97	–
M1	2	12.07–14.95	13.51	2	16.52–17.77	17.15
M2	5	16.67–20.48	19.35	6	17.85–19.35	18.56
M3	5	18.80–21.39	19.96	5	16.99–19.50	18.30
i1	1	12.86	–	1	6.31	–
p2	2	9.97–11.33	10.65	2	7.03–7.44	7.24
p3	2	13.04–13.67	13.36	2	8.33–8.56	8.45
p4	2	13.65–14.05	13.85	2	8.94–9.33	9.14
m1	2	14.67–15.67	15.17	2	11.23–12.25	11.74
m2	2	17.73–19.36	18.55	2	12.63–13.26	12.95
m3	1	23.61		1	12.84	
***Rusa unicolor***						
M1	1	17.15	–	1	20.10	–
M2	2	20.67–22.88	21.78	2	23.06–27.07	25.07
M3	1	25.37	–	1	24.97	–
p3	1	17.29	–	1	9.26	–
p4	1	17.71	–	2	10.34–13.35	11.85
m1	2	18.64–20.84	19.74	2	14.39–14.59	14.49
m2	3	22.77–23.82	23.33	3	15.37–15.61	15.46
m3	3	30.78–34.57	32.67	3	15.49–17.85	16.79

**Mandibles and lower dentition**: twenty one mandibles range from fragmentary (preserving only the broken corpus) to nearly complete (lacking only the ascending ramus and coronoid process) individuals (Fig. 15F–O) (for measurements, see Appendix 5). The mandibular symphyses are almost complete, but all incisors are missing. The protoconulid of the p2 is poorly-developed or absent (Fig. 15F, H, J).

Lower third and fourth premolars exhibit a well developed metaconid which projects obliquely in occlusal view, posterior to the entoconid (Fig. 15F, H, J) (for measurements, see Tab. 12). The latter conid joins the posthypocristid, forming a back valley on moderately worn teeth. The metaconid is bifurcated (two separated flanges: pre- and postmetacristids) on the p4. All lower molars are morphologically characterized by their brachyodont crowns and well-developed stylids (parastylid, metastylid, and entostylid), ectostylids (basal pillars), and anterior cingulids (also called “goat fold”) (Fig. 15F–Q). On the m3, the posterior ectostylid is absent (Fig. 15F, G, J–Q). The third lobe is ring-shaped as it is present on the recent specimens (e.g., MNHN-ZMO-1901-547, MNHN-ZMO-1988-153, ZSM-1951-70, and ZSM-1961-3) (Fig. 15F, P). But the third lobe is sometimes small and poorly-developed, as observed from the recent specimen ZSM-1963-27 (Fig. 15J, L, N). The back fossa is present on unworn to slightly worn teeth (Fig. 15F, P), but absent on moderately to heavily worn ones (Fig. 15L, N). The posthypoconulidcristid is well-developed, a small crest protruding slightly more posterolingually (Fig. 15F).

**Figure 16.**
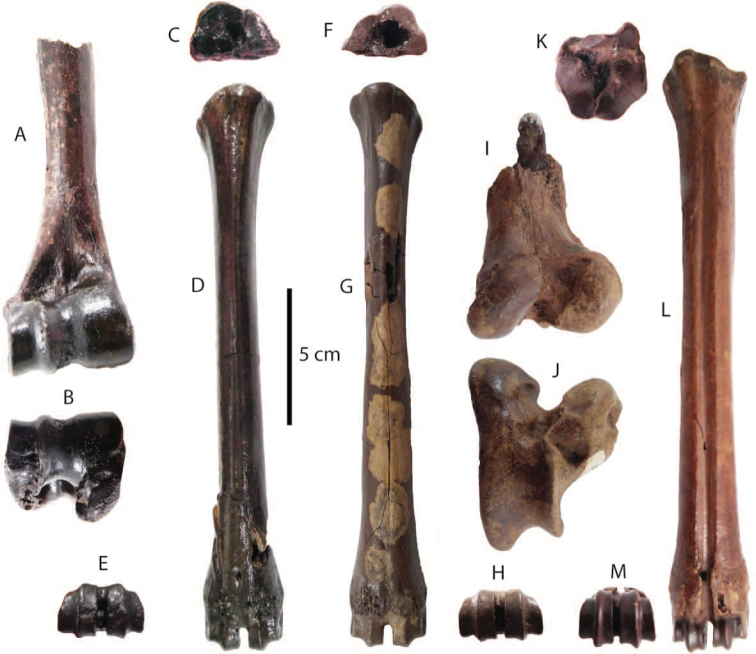
Postcranial remains of *Axis axis* from Khok Sung: **A–B** DMR-KS-05-04-11-32, a right distal humerus in anterior (**A**) and distal (**B**) views **C–E** DMR-KS-05-03-18-2, a right metacarpus in proximal (**C**), anterior (**D**), and distal (**E**) views **F–H** DMR-KS-05-03-19-37, a left metacarpus in proximal (**F**), anterior (**G**), and distal (**H**) views **I–J** DMR-KS-05-03-27-4, a right distal femur in posterior (**I**) and distal (**J**) views **K–M** DMR-KS-05-03-26-3, a right metatarsus in proximal (**K**), anterior (**L**), and distal (**M**) views.

**Postcranial remains**: postcranial bones include isolated humeri (Fig. 16A–B), metacarpi (Fig. 16C–H), a femur (Fig. 16I, J), and metatarsi (Fig. 16K–M). The humerus and femur are fragmentary. We identify here these fossil postcranial bones based on the size and proportion compared with the extant specimens (Tab. 13 and Appendices 1, 7, 9–10, and 12).

**Table 13.** Proportional indices of postcranial remains of identified ruminant taxa from Khok Sung.

**Scapula**												
**Specimen**	**Taxa**	**HS/Ld**	**DHA/Ld**	**Ld/SLC**	**LG/BG**	**GLP/LG**	**SLC/BG**					
DMR-KS-05-03-26-2	*Bubalus arnee*	1.50	1.28	3.89	1.20	1.30	1.12					
DMR-KS-05-02-20-4	*Bubalus arnee*	1.39	1.42	4.09	1.23	1.26	0.96					
DMR-KS-05-06-24-4	*Panolia eldii*	1.95	1.90	4.62	1.10	1.27	0.74					
**Humerus**												
**Specimen**	**Taxa**	**GL/Bp**	**GL/Dp**	**GL/Bd**	**GL/Dd**	**Bp/Bd**	**Dp/Dd**	**Bp/Dp**	**Bd/Dd**	**Bd/BT**		
DMR-KS-05-03-20-2(1)	*Bos sauveli*	–	–	–	–	–	–	–	0.99	1.04		
DMR-KS-05-03-00-62	*Bos gaurus*	–	–	3.41	3.66	–	–	–	1.07	1.06		
DMR-KS-05-05-1-1	*Bos gaurus*	2.91	2.74	3.44	3.67	1.18	1.34	0.94	1.07	1.05		
DMR-KS-05-03-31-1	*Bubalus arnee*	3.57	3.25	4.30	4.77	1.21	1.47	0.91	1.11	1.05		
DMR-KS-05-03-31-8	*Bubalus arnee*	3.54	3.29	4.25	4.74	1.20	1.44	0.93	1.11	1.03		
DMR-KS-05-03-13-4	*Axis axis*	–	–	–	–	–	–	–	1.02	1.09		
DMR-KS-05-04-11-32	*Axis axis*	–	–	–	–	–	–	–	1.06	1.07		
DMR-KS-05-03-17-17	*Axis axis*	–	–	–	–	–	–	–	1.12	1.04		
DMR-KS-05-04-11-35	*Panolia eldii*	–	–	–	–	–	–	–	1.12	1.13		
DMR-KS-05-03-18-1	*Panolia eldii*	–	–	–	–	–	–	0.82	–	–		
DMR-KS-05-03-15-43	*Rusa unicolor*	–	–	–	–	–	–	–	1.14	1.12		
**Ulna and radius**												
**Specimen**	**Taxa**	**PL/Bp**	**PL/Dp**	**PL/Bd**	**PL/Dd**	**Bp/Bd**	**Dd/Dp**	**Bp/Dp**	**Bd/Dd**	**Bp/BFp**	**Bd/BFd**	**GL/LO**
DMR-KS-05-03-00-61	*Bubalus arnee*	2.87	5.76	3.04	4.63	1.06	1.24	2.00	1.52	1.15	1.11	3.86
DMR-KS-05-03-31-2	*Bubalus arnee*	3.15	5.85	3.25	4.61	1.03	1.27	1.86	1.42	1.09	1.12	3.48
DMR-KS-05-03-31-9	*Bubalus arnee*	3.09	5.88	3.24	4.55	1.05	1.29	1.90	1.40	1.10	1.12	3.45
DMR-KS-05-03-31-10	*Panolia eldii*	5.06	9.51	5.35	9.32	1.06	1.02	1.88	1.74	1.07	1.14	–
DMR-KS-05-04-11-3	*Panolia eldii*	4.83	9.09	5.54	8.70	1.15	1.04	1.88	1.57	1.11	1.06	–
DMR-KS-05-03-19-16	*Panolia eldii*	4.93	8.93	4.87	6.62	0.99	1.35	1.81	1.36	1.22	1.04	–
DMR-KS-05-03-25-9	*Rusa unicolor*	–	–	–	–	–	–	1.90	–	1.03	–	–
DMR-KS-05-03-19-14	*Rusa unicolor*	–	–	–	–	–	–	1.70	–	1.04	–	–
DMR-KS-05-03-26-19	*Rusa unicolor*	–	–	–	–	–	–	–	1.34	–	1.05	–
**Femur**												
**Specimen**	**Taxa**	**GL/Bp**	**GL/Dp**	**GL/Bd**	**GL/Dd**	**Bp/Bd**	**Dd/Dp**	**Bp/Dp**	**Dd/Bd**			
DMR-KS-05-03-9-2	*Bos gaurus*	3.37	6.29	3.92	3.03	1.17	2.07	1.87	1.29			
DMR-KS-05-04-1-1	*Bubalus arnee*	2.79	5.54	3.48	2.85	1.25	1.95	1.99	1.22			
DMR-KS-05-04-1-2	*Bubalus arnee*	2.67	5.26	3.38	2.82	1.27	1.86	1.97	1.20			
DMR-KS-05-03-20-8	*Bubalus arnee*	–	–	–	–	–	–	–	1.46			
DMR-KS-05-03-27-4	*Axis axis*	–	–	–	–	–	–	–	1.37			
DMR-KS-05-03-27-11	*Panolia eldii*	–	–	–	–	1.26	2.23	2.11	1.33			
DMR-KS-05-03-17-36	*Panolia eldii*	–	–	–	–	1.21	2.06	1.93	1.29			
DMR-KS-05-03-28-20	*Panolia eldii*	–	–	–	–	–	–	–	1.34			
DMR-KS-05-04-05-38	*Panolia eldii*	–	–	–	–	–	–	1.92	–			
DMR-KS-05-03-00-119	*Panolia eldii*	–	–	–	–	–	–	–	1.38			
DMR-KS-05-03-19-2	*Panolia eldii*	–	–	–	–	–	–	–	1.41			
DMR-KS-05-08-16-1	*Panolia eldii*	–	–	–	–	–	–	1.84	–			
DMR-KS-05-04-11-2	*Rusa unicolor*	–	–	–	–	–	–	–	1.27			
DMR-KS-05-03-19-7	*Rusa unicolor*	–	–	–	–	–	–	1.51	–			
DMR-KS-05-03-12-2*	*Rusa unicolor*	–	–	–	–	–	–	1.52	–			
DMR-KS-05-04-30-9	*Rusa unicolor*	–	–	–	–	–	–	–	1.27			
DMR-KS-05-04-19-10	*Rusa unicolor*	–	–	–	–	–	–	–	1.11			
**Tibia**												
**Specimen**	**Taxa**	**GL/Bp**	**GL/Dp**	**GL/Bd**	**GL/Dd**	**Bp/Bd**	**Dp/Dd**	**Bp/Dp**	**Bd/Dd**			
DMR-KS-05-04-1-11	*Bubalus arnee*	3.24	3.43	4.82	6.03	1.49	1.76	1.06	1.25			
DMR-KS-05-04-1-3	*Bubalus arnee*	3.31	3.50	5.01	6.29	1.51	1.80	1.06	1.25			
DMR-KS-05-03-20-9	*Bubalus arnee*	3.21	3.83	4.60	6.29	1.43	1.64	1.19	1.37			
DMR-KS-05-03-28-16	*Rusa unicolor*	4.00	4.38	6.68	8.48	1.67	1.94	1.10	1.27			
**Metacarpus**												
**Specimen**	**Taxa**	**GL/Bp**	**GL/Dp**	**GL/Bd**	**GL/Dd**	**Bp/Bd**	**Dp/Dd**	**Bp/Dp**	**Bd/Dd**			
DMR-KS-05-03-26-27	*Bos gaurus*	3.66	5.57	3.96	7.66	1.08	1.37	1.52	1.93			
DMR-KS-05-03-26-3(1)	*Bubalus arnee*	2.68	4.17	2.64	4.87	0.98	1.17	1.55	1.85			
DMR-KS-05-03-18-2	*Axis axis*	6.50	9.99	6.69	10.55	1.03	1.06	1.54	1.58			
DMR-KS-05-03-22-28	*Axis axis*	–	9.59	6.81	10.36	–	1.08	–	1.52			
DMR-KS-05-03-08-2	*Axis axis*	6.36	8.79	6.18	10.18	0.97	1.16	1.38	1.65			
DMR-KS-05-03-19-3	*Axis axis*	6.58	9.06	6.30	10.42	0.96	1.15	1.38	1.65			
DMR-KS-05-03-19-37	*Axis axis*	7.14	11.05	6.84	10.75	0.96	0.97	1.55	1.57			
DMR-KS-05-04-30-20	*Axis axis*	6.87	10.36	–	–	–	–	1.51	–			
DMR-KS-05-03-24-2	*Panolia eldii*	6.39	8.99	6.57	10.41	1.03	1.16	1.41	1.58			
DMR-KS-05-03-17-26	*Rusa unicolor*	5.97	7.57	6.06	9.10	1.02	1.20	1.27	1.50			
**Metatarsus**												
**Specimen**	**Taxa**	**GL/Bp**	**GL/Dp**	**GL/Bd**	**GL/Dd**	**Bp/Bd**	**Dp/Dd**	**Bp/Dp**	**Bd/Dd**			
DMR-KS-05-04-1-8	*Bubalus arnee*	3.80	4.59	3.17	5.54	0.83	1.21	1.21	1.75			
DMR-KS-05-04-1-6	*Bubalus arnee*	3.88	4.39	3.11	5.67	0.80	1.29	1.13	1.82			
DMR-KS-05-03-28-30	*Bubalus arnee*	4.25	4.28	3.40	6.38	0.80	1.49	1.01	1.88			
DMR-KS-05-03-26-3	*Axis axis*	7.21	6.91	6.99	9.16	0.97	1.33	0.96	1.31			
DMR-KS-05-03-15-14	*Axis axis*	6.84	7.37	6.15	9.22	0.90	1.25	1.08	1.50			
DMR-KS-05-03-29-30	*Axis axis*	6.91	6.82	6.52	8.58	0.94	1.26	0.99	1.32			
DMR-KS-05-03-28-17	*Panolia eldii*	8.05	7.71	7.73	11.69	0.96	1.52	0.96	1.51			
DMR-KS-05-03-25-8	*Panolia eldii*	7.81	7.47	7.22	11.57	0.92	1.55	0.96	1.60			
DMR-KS-05-03-15-15	*Panolia eldii*	8.08	7.44	7.37	11.29	0.91	1.52	0.92	1.53			
DMR-KS-05-03-19-11	*Rusa unicolor*	6.64	6.86	6.49	9.20	0.98	1.34	1.03	1.42			

######## Taxonomic remarks and comparisons.

The antlers are useful to distinguish among the cervids, whereas the morphologies of lower cheek teeth are identical among *Axis*. The skulls, antlers, and teeth from Khok Sung are morphologically similar to those observed from recent *Axis axis*. This suggests a morphological stasis in the evolution of antlers and teeth for this species.

**Figure 17.**
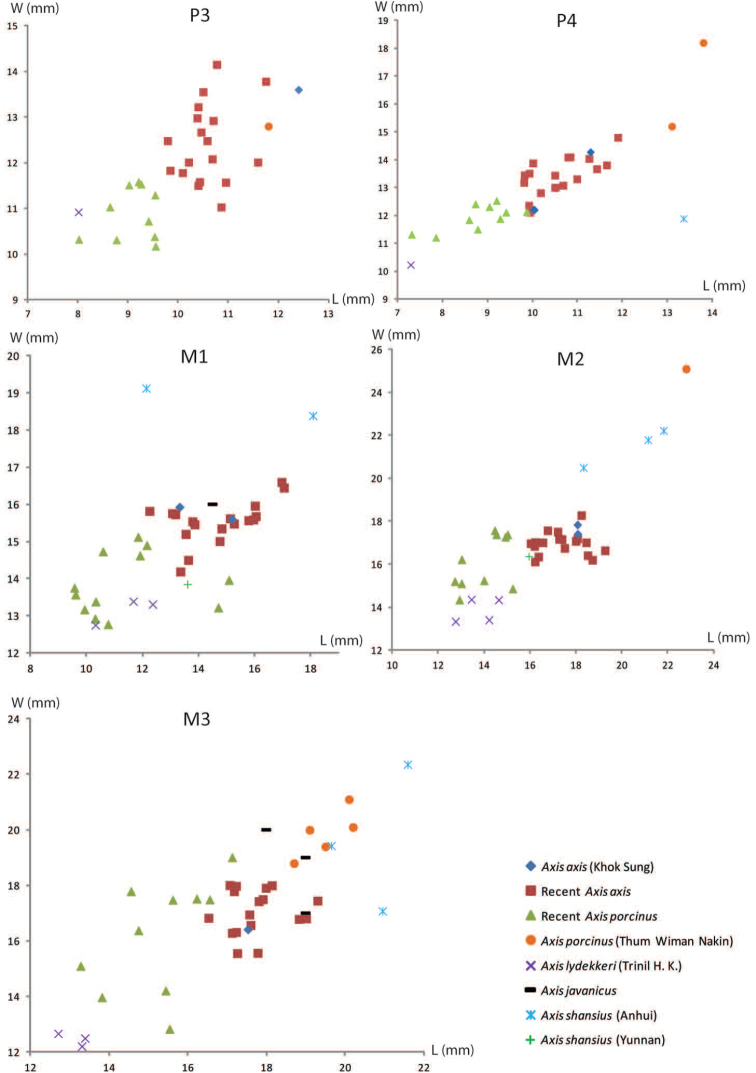
Scatter diagrams of upper cheek tooth (P3–M3) lengths and widths of recent and fossil *Axis*. Data of *Axis javanicus* (Trinil H. K.) and *Axis porcinus* (Thum Wiman Nakin) are from von Koenigswald (1933) and Tougard (1998), respectively.

**Figure 18.**
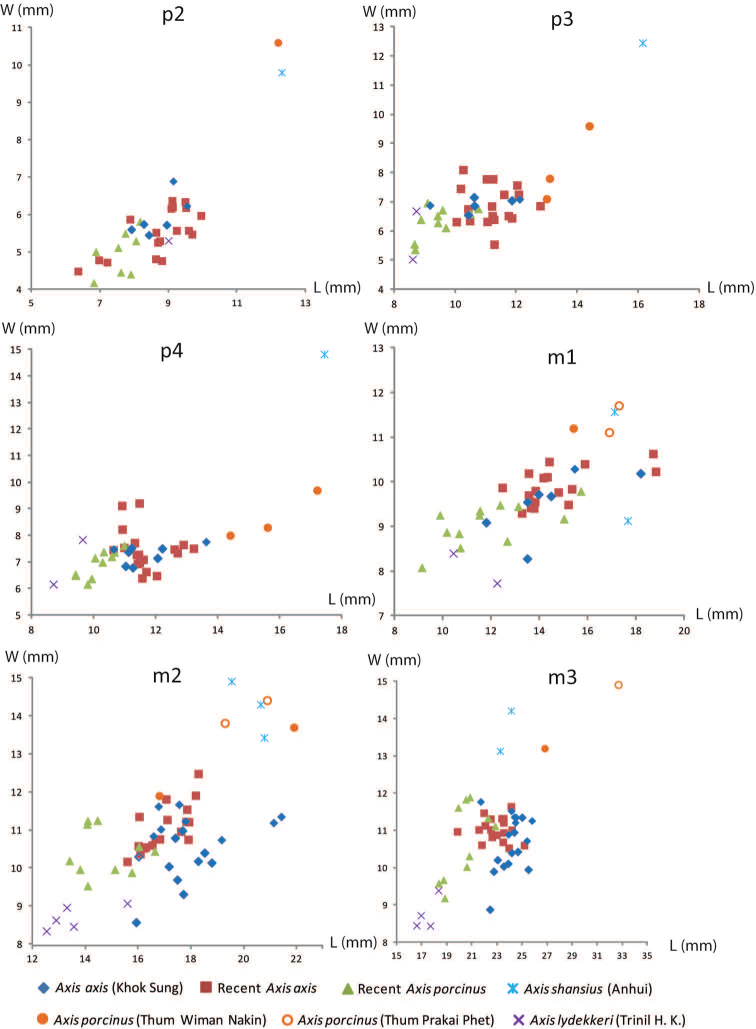
Scatter diagrams of lower cheek tooth (p2–m3) lengths and widths of recent and fossil *Axis*. Data of *Axis javanicus* (Trinil H. K.) and *Axis porcinus* (Thum Wiman Nakin and Thum Prakai Phet) are from von Koenigswald (1933), Tougard (1998), and Filoux et al. (2015), respectively.

Based on our observation on the extant comparative material of *Axis axis* (e.g., the specimens MNHN-ZMO-1901-547, MNHN-ZMO-1988-153, ZSM-1951-70, and ZSM-1958-88), we thus demonstrate some dental morphological variation within species. The m3 of *Axis axis* appears more morphologically variable than the other molars, such as the more or less developed posterior talonids and the presence/absence of back fossae. The cheek teeth of extant *Axis axis* are relatively similar to those of *Axis porcinus* (e.g., the specimens MNHN-ZMO-1904-60, MNHN-ZMO-1962-4188, ZSM-1968-493, and ZSM-1969-63). However, *Axis axis* differs from *Axis porcinus* in having less developed anterior cingulids on the lower molars and the presence of back fossae on the m3. Recent *Axis axis* represents an intermediate size between *Axis porcinus* and two cervid species (*Panolia eldii* and *Rusa unicolor*) (Tab. 14). *Axis axis* from Khok Sung also follows the size tendency of recent populations (Figs 17 and 18).

**Table 14.** Body mass prediction of Khok Sung ruminants using second molar variables, compared to relative sizes of the recent population (Grzimek 1975, Lekagul and McNeely 1988, Nowak 1999). The predictive equations follow Janis (1990: table. 16.8).

	Body mass (kg)			
**Cervidae**	**Khok Sung**			**Recent**
**Taxa**	**N**	**Range**	**Mean**	**Range**
*Axis axis*	17	67.6–127.6	90.8	75–100
*Panolia eldii*	7	99.1–157.6	133.5	95–150
*Rusa unicolor*	5	215.6–332.3	255.4	100–350
**Bovidae**	**Khok Sung**			**Recent**
**Taxa**	**N**	**Range**	**Mean**	**Range**
*Bos sauveli*	3	660.8–756.0	720.5	700–900
*Bos gaurus*	3	808.5–940.8	873.2	700–1000
*Bubalus arnee*	12	694.5–1243.0	944.7	700–1200

Compared to other Pleistocene cervid species, the cheek teeth of *Axis axis* from Khok Sung are smaller than those of *Axis shansius* from Anhui and Yunnan (China) and of *Axis javanicus* from Ngandong and Buitenzorg in Java and Carnul Cave in India, but are larger than those of *Axis lydekkeri* from Trinil H. K. (Java) (Figs 17 and 18). Although, *Axis javanicus* is closely related to or even synonymous with *Axis axis* according to Meijaard and Groves (2004), it is considered as a valid species due to studies of the geometric morphometric analysis performed on the teeth (Gruwier et al. 2015). According to the scatter diagrams of the dental sizes (Figs 17 and 18), Thum Wiman Nakin and Thum Prakai Phet fossil teeth assigned to *Axis porcinus* (Tougard 1998, Filoux et al. 2015) are much larger than their extant populations and those from Khok Sung. Although the Pleistocene hog deer probably show clinal variation in size (Bergmann’s rule) in response to colder climates. The fossil teeth attributed to *Axis porcinus* from Thum Wiman Nakin and Thum Prakai Phet, identified by Tougard (1998) and Filoux et al. (2015), possibly reveal a double size (or more) of the recent population. We suggest that these fossils likely belong to either other larger or new cervid species that lived during the Pleistocene across mainland Southeast Asia. We also cast doubt on the occurrence of *Axis porcinus* in the Middle Pleistocene of Boh Dambang, Cambodia (Demeter et al. 2013). The existence of *Axis porcinus* in Southeast Asia during the Middle Pleistocene is still doubtful.

###### Genus *Panolia* Gray, 1843

####### Panolia eldii

Taxon classificationAnimaliaArtiodactylaCervidae

(M’Clelland, 1842)

######## Referred material.

A cranium with a right partial antler, DMR-KS-05-04-20-4; a right P2, DMR-KS-05-03-15-11; two left M1—DMR-KS-05-03-00-24 and DMR-KS-05-03-00-25; six M2—DMR-KS-05-03-00-23 (right), DMR-KS-05-03-30-5 (right), DMR-KS-05-04-3-4 (right), DMR-KS-05-03-30-6 (left posterior lobe), DMR-KS-05-03-27-7 (left), and DMR-KS-05-04-3-5 (left); five M3—DMR-KS-05-03-27-6 (right), DMR-KS-05-04-9-1 (right), DMR-KS-05-04-8-3 (right), DMR-KS-05-03-00-22 (left), and DMR-KS-05-04-9-2 (left); two left mandibles—DMR-KS-05-03-27-2 (with p2–m3) and DMR-KS-05-04-9-5 (with p2–m2); a right i1, DMR-KS-05-03-29-2; a right scapula, DMR-KS-05-06-24-4; a left humerus, DMR-KS-05-04-11-35; a right fragmentary humerus, DMR-KS-05-03-18-1 (proximal part); three radii—DMR-KS-05-03-31-10 (right), DMR-KS-05-04-11-3 (right), and DMR-KS-05-03-19-16 (left); a right metacarpus, DMR-KS-05-03-24-2; two right femora—DMR-KS-05-03-27-11 and DMR-KS-05-03-17-36; five fragmentary femora—DMR-KS-05-04-05-38 (right proximal part), DMR-KS-05-03-28-20 (right distal part), DMR-KS-05-03-00-119 (right distal part), DMR-KS-05-03-19-2 (right distal part), and DMR-KS-05-08-16-1 (left proximal part); three left metatarsi—DMR-KS-05-03-25-8, DMR-KS-05-03-28-17, and DMR-KS-05-03-15-15.

######## Material description.

**Figure 19.**
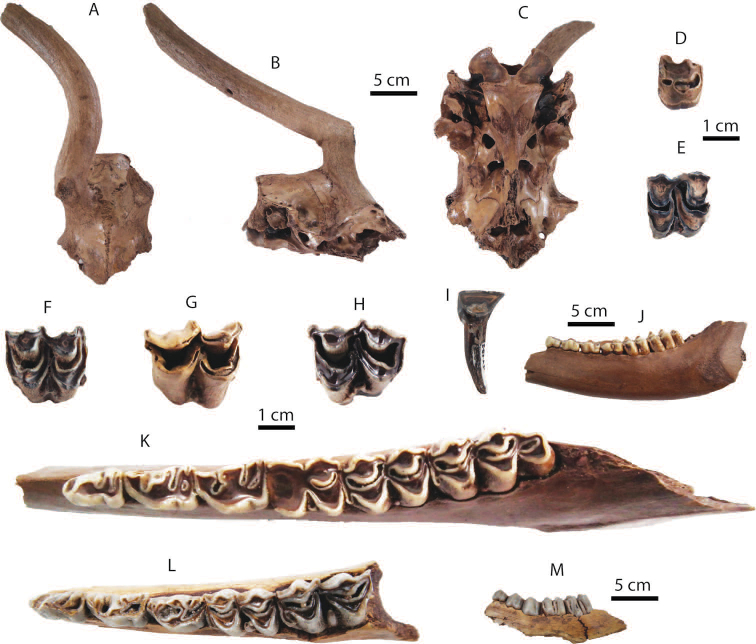
Remains of *Panolia eldii* from Khok Sung: **A–C** DMR-KS-05-04-20-4, a cranium in dorsal (**A**), lateral (**B**), and ventral (**C**) views **D** DMR-KS-05-03-15-11, a right P2 **E** DMR-KS-05-03-00-24, a left M1 **F** DMR-KS-05-03-00-23, a right M2 **G** DMR-KS-05-03-27-6, a left M3 **H** DMR-KS-05-04-9-2, a left M3 **I** DMR-KS-05-03-29-2, a right i1 in lingual view **J–K** DMR-KS-05-03-27-2, a left mandible in lateral (**J**) and occlusal (**K**) views **L–M** DMR-KS-05-04-9-5, a left mandible in occlusal (**L**) and lateral (**M**) views. All teeth are shown in occlusal view.

**Cranium and upper dentition**: DMR-KS-05-04-20-4 is an incomplete cranium, lacking the whole anterior parts (nasal, jugal, palatine, and maxilla) (Fig. 19A–C) (for measurements, see Appendix 4). This specimen is a juvenile individual according to the incompletely fused sutures. The basioccipital and basisphenoid are triangular in outline and have straight lateral edges (Fig. 19C), different from those of *Axis*, and as observed on the recent skull of *Panolia eldii* (e.g., MNHN-ZMO-1937-157, MNHN-ZMO-1944-307, MNHN-ZMO-2011-190, and NMW-2975). The foramina ovale of DMR-KS-05-04-20-4 are more circular and open more anteriorly than those of *Axis*. The right partial antler contains a half of the slender main beam, but lacks a brow tine entirely (Fig. 19A, B). The divergent angle between the main beam and the brow tine is of about 110°, similar to recent skulls of *Panolia eldii* (e.g., THNHM-M-125). The antler surface is smooth and the burr is poorly developed in relation to the ontogenetic stages. The preserved shed antler shows a typical character of *Panolia eldii*, whose main beams strongly project and curve laterally (Fig. 19A).

P2 exhibits a prominent medial crista which divides the fossette into two islands (Fig. 19D). The separated anterior fossette is larger than the posterior one. On the upper molars, the buccal styles, anterior cingula, and entostyles are distinct (for measurements, see Tab. 12). The entostyle is bifurcated (Fig. 19E–H). The metaconule fold (spur) is poorly developed. The posterior lobe of the M3 is reduced in width (Fig. 19G, H). The buccal wall of the posterior lobe is oblique in occlusal view.

**Mandibles and lower dentition**: Two mandibles (DMR-KS-05-03-27-2: Fig. 19J, K and DMR-KS-05-04-9-5: Fig. 19L, M) are nearly complete, preserving the bodies with cheek tooth rows (for measurements, see Appendix 5). The first specimen also preserves a partial ramus and is more complete than the second one in which the mandibular body is broken.

An isolated i1 is spatulate (Fig. 19I). Lower premolars show more complex patterns compared to *Axis* (e.g., the bifurcation of the metaconid on the p3, the irregular shape of the posterior valley, and the presence of more developed pre- and postprotoconulidcristids) (Fig. 19K, L). Lower molars display well-developed anterior cingulids and stylids (for measurements, see Tab. 12). The m3 is characterized by the presence of a posterior ectostylid (Fig. 19K). The shape of the posterior lobe of the m3 resembles that of *Axis axis*.

**Figure 20.**
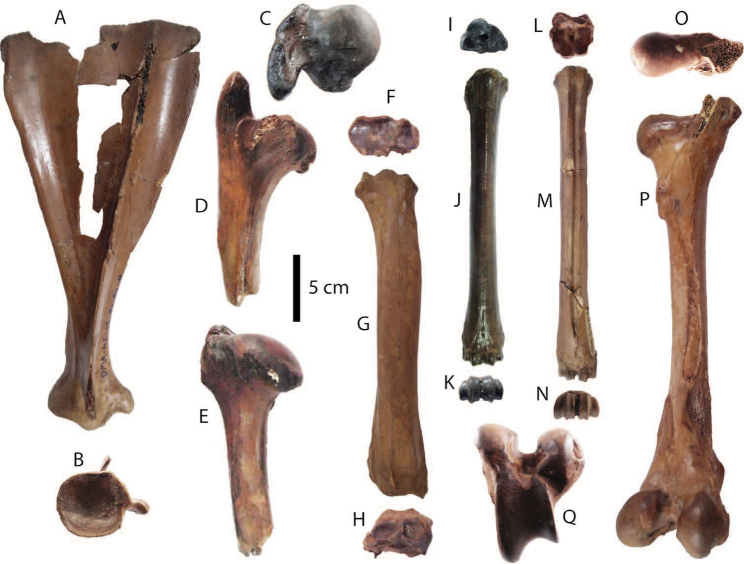
Postcranial remains of *Panolia eldii* from Khok Sung: **A–B** DMR-KS-05-06-24-4, a right scapula in lateral (**A**) and distal (**B**) views **C–E** DMR-KS-05-03-18-1, a right proximal humerus in proximal (**C**), anterior (**D**), and posterior (**E**) views **F–H** DMR-KS-05-03-31-10, a right radius in proximal (**F**), anterior (**G**), distal (**H**) views **I–K** DMR-KS-05-03-24-2, a right metacarpus in proximal (**I**), anterior (**J**), and distal (**K**) views **L–N** DMR-KS-05-03-25-8, a left metatarsus in proximal (**L**), anterior (**M**), distal (**N**) views **O–Q** DMR-KS-05-03-17-36, a right femur in proximal (**O**), posterior (**P**), distal (**Q**) views.

**Postcranial remains**: postcranial bones include a scapula (Fig. 20A, B), humeri (Fig. 20C–E), radii, a metacarpus (Fig. 20I–K), femora (Fig. 20O–Q), and metatarsi (Fig. 20L–N). They are almost complete. We identify these postcranial bones based on the correlation of size and proportion with the extant specimens of *Panolia eldii* (Tab. 13, and Appendices 1, 6–10, and 12).

######## Taxonomic remarks and comparisons.

Several authors consider Eld’s deer as belonging to either the genus *Cervus* (e.g., Lekagul and McNeely 1988, Tougard 2001, Gruwier et al. 2015) or *Rucervus* (e.g., Grubb 2005). However, Groves and Grubb (2011) suggested that placement of the Eld’s deer in the genus *Panolia* is an acceptable alternative based on mtDNA analysis (Pitra et al. 2004).

**Figure 21.**
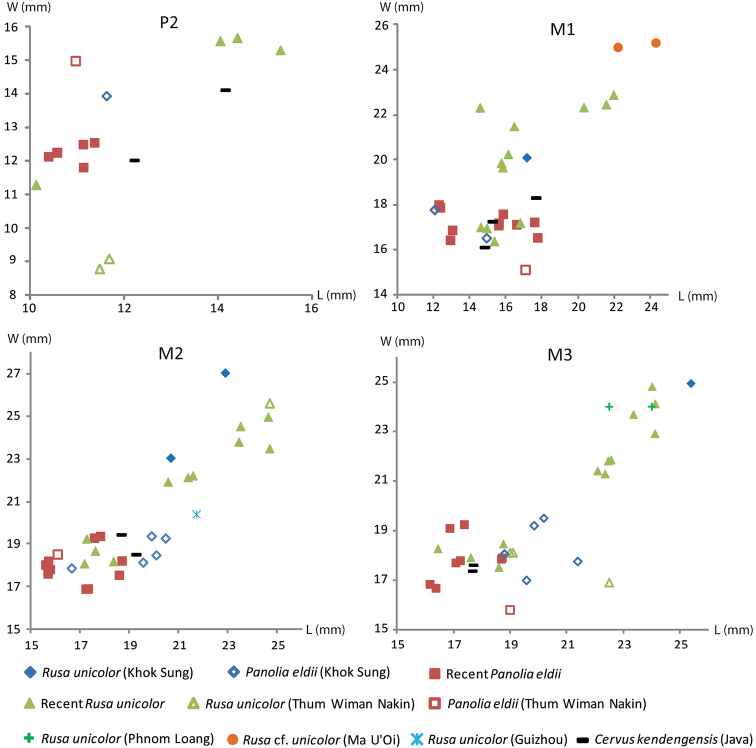
Scatter diagrams of upper cheek tooth (P2, M1, M2, and M3) lengths and widths of some recent and fossil large cervids. The measurements of fossil cervids from the caves of Phnom Loang, Thum Wiman Nakin, and Ma U’Oi are obtained from Beden and Guérin (1973), Tougard (1998), and Bacon et al. (2004), respectively.

**Figure 22.**
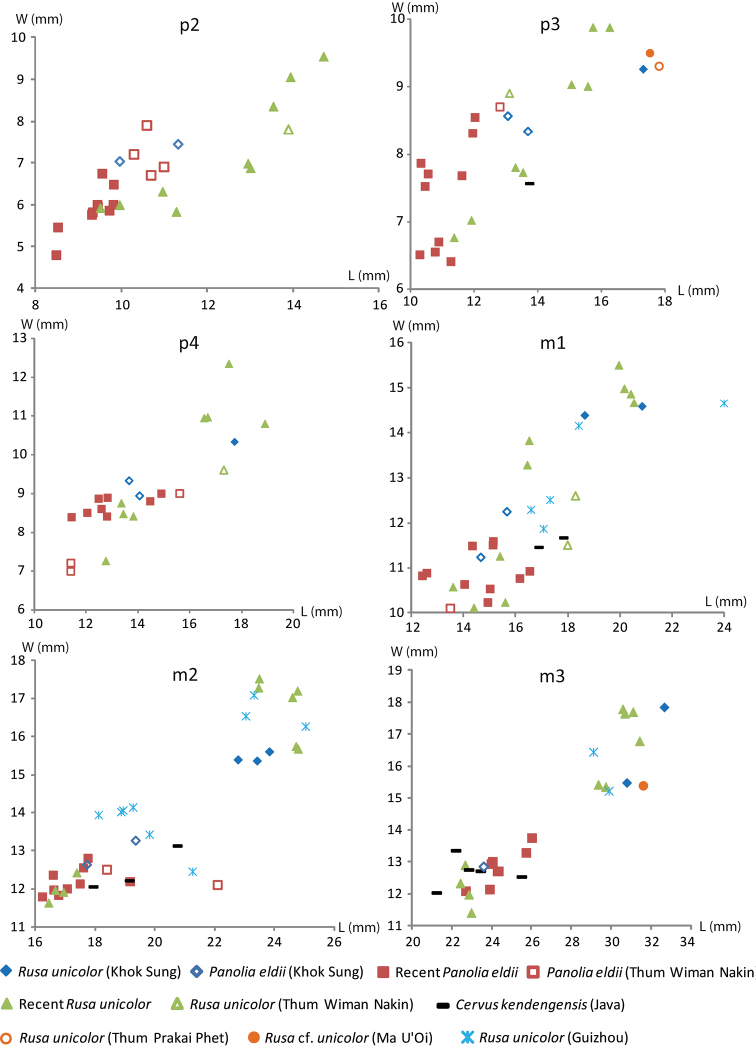
Scatter diagrams of lower cheek tooth (p2–m3) lengths and widths of some recent and fossil large cervids. The measurements of fossil cervids from the caves of Thum Wiman Nakin, Thum Prakai Phet, and Ma U’Oi are obtained from Tougard (1998), Filoux et al. (2015), and Bacon et al. (2004), respectively.

The shed antler of the Eld’s deer, *Panolia eldii*, is characterized by bow- or lyre-like shapes, long, noticeable, and laterally bending-main beams with a distal portion curving medially, and small ornamented branches of brow tines. The cheek teeth of *Panolia eldii* differ from those of *Axis axis* in having a larger size, a more complex wear pattern of the mesolingual conids on the p3, more developed anterior cingulids on the lower molars, and a posterior ectostylid on the m3. The Khok Sung specimens assigned to *Panolia eldii* are similar in morphology to the extant specimens. As demonstrated by the body mass estimation (Tab. 14) and scatter diagrams (Figs 21 and 22), *Panolia eldii* from Khok Sung is also comparable in size to recent populations, to that from Thum Wiman Nakin, and to some fossil species (e.g., *Cervus kendengensis* from the Pleistocene of Bangle and Kali Gedeh in Java). However, we suggest that some isolated teeth of cervids from Thum Wiman Nakin (Tougard 1998) reveal an improper taxonomic identification. The P2 (TF 3371 and TF 4570), p2 (TF 3938, TF 3313, TF 3358, and TF 3983), p3 (TF 3373), and m2 (TF 4025), attributed to *Panolia eldii*, may belong to other cervids (possibly *Rusa unicolor*) due to their larger sizes. Our identification thus confirms the existence of *Panolia eldii* in Thailand during the late Middle Pleistocene.

###### Genus *Rusa* Hamilton-Smith, 1827

####### Rusa unicolor

Taxon classificationAnimaliaArtiodactylaCervidae

(Kerr, 1792)

######## Referred material.

Three right antlers—DMR-KS-05-03-20-11 (nearly complete specimen), DMR-KS-05-03-26-2 (fragment), and DMR-KS-05-03-28-23 (fragment); a right M1, DMR-KS-05-03-22-10; two left M2—DMR-KS-05-04-9-3 and DMR-KS-05-04-3-3; a left M3, DMR-KS-05-03-31-1; two right mandibles—DMR-KS-05-03-31-2 (with m2) and DMR-KS-05-03-13 (with p4–m3); two left mandibles—DMR-KS-05-03-00-101 (with p3–m3) and DMR-KS-05-03-27-4 (with m3); a right m1, DMR-KS-05-03-00-5; a left fragmentary humerus, DMR-KS-05-03-15-43 (distal part); three right fragmentary radii—DMR-KS-05-03-25-9 (proximal part), DMR-KS-05-03-19-14 (proximal part), and DMR-KS-05-03-26-19 (distal part); a left metacarpus, DMR-KS-05-03-17-26; six fragmentary femora—DMR-KS-05-03-19-7 (right proximal part), DMR-KS-05-03-12-2 (right proximal part), DMR-KS-05-04-11-2 (right distal part), DMR-KS-05-03-26-5 (left proximal part), DMR-KS-05-04-30-9 (left distal part), and DMR-KS-05-04-19-10 (left distal part); a right tibia, DMR-KS-05-03-28-16; a right metatarsus, DMR-KS-05-03-19-11

######## Material description.

**Figure 23.**
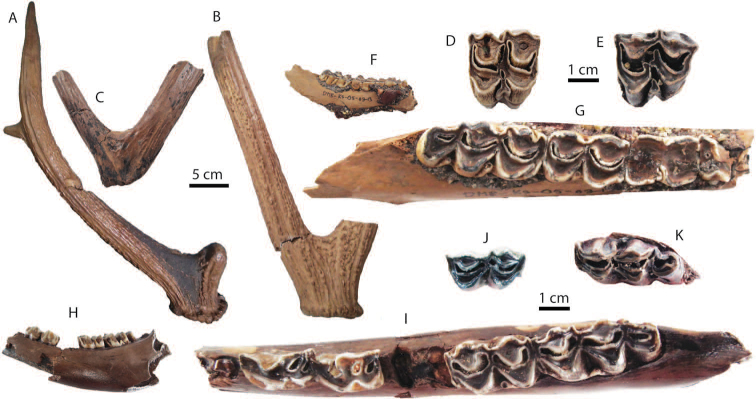
Remains of *Rusa unicolor* from Khok Sung: **A** DMR-KS-05-03-20-11, a right antler in lateral view **B** DMR-KS-05-03-26-2, a right antler fragment in lateral view **C** DMR-KS-05-03-28-23, a right antler fragment in medial view **D** DMR-KS-05-04-9-3, a left M2 **E** DMR-KS-05-03-31-1, a left M3 **F–G** DMR-KS-05-03-13, a right mandible in lateral (**F**) and occlusal (**G**) views **H–I** DMR-KS-05-03-00-101, a left mandible in lateral (**H**) and occlusal (**I**) views **J** DMR-KS-05-03-00-5, a right m1 **K** DMR-KS-05-03-27-4, a left m3. All isolated teeth are shown in occlusal view.

**Antlers**: DMR-KS-05-03-20-11 is a nearly complete antler, slightly broken at the middle part of the main beam (Fig. 23A). The fragmentary antler DMR-KS-05-03-26-2 comprises a burr, a broken brow tine, and a half of the main beam (Fig. 23B). The specimen DMR-KS-05-03-28-23 preserves the broken brow tine and main beam (Fig. 23C). The antler surface is rough. The shed antlers are morphologically characterized by three main tines, a long and slender main beam, a forked construction at the tip, and a well-developed burr (Fig. 23A–C). On the apical bifurcation, the postero-internal tine is much shorter than the antero-external one. The main beam and brow tine are also much more robust, compared to the extant males of *Axis porcinus* (e.g., the specimen MNHN-ZMO-1904-60 and NMW-2546). The divergent angle between the main beam and brow tine ranges from 50° to 90°. The shed antlers of *Rusa unicolor* are different from those of *Axis axis* in having slightly rougher surfaces, more divergent insertion relative to the frontal orientation, a shorter main beam, and a smaller angle between the main beam and the brow tine, and in lacking small-ornamented tines or knobs on the brow tine (Fig. 23A–C). These characters match well the recent *Rusa unicolor*.

**Upper dentition**: upper molars are robust (Tab. 12) and show well-developed styles (particularly the mesostyle), anterior cingula, and entostyles (Fig. 23D, E). The entostyle is bifurcated, like in *Panolia eldii*, in relation to the moderately to strongly worn teeth. The fossettes are present at least in the middle stage of wear. The metaconule fold is poorly developed or sometimes absent. On the M3, the anterior lobe is wider than the posterior one (Fig. 23E).

**Mandibles and lower dentition**: four mandibles are incomplete (for measurements, see Appendix 5). The specimens DMR-KS-05-03-13 (Fig. 23F, G) and DMR-KS-05-03-00-101 (Fig. 23H, I) preserve a partially broken mandibular body. The manidibles DMR-KS-05-03-31-2 and DMR-KS-05-03-27-4 are very fragmentary. All lower cheek teeth of *Rusa unicolor* are obviously larger than those of other Khok Sung cervids (Tab. 12). Lower molars display cervid-like patterns, such as well developed styles, anterior cingulids, and ectostylids (Fig. 23J, K). On the m3, the posterior lobe of the talonid in *Rusa unicolor* is more developed than those in *Axis*. Moreover, the posterior ectostylid is present (Fig. 23G, I, K), unlike in *Axis*.

**Figure 24.**
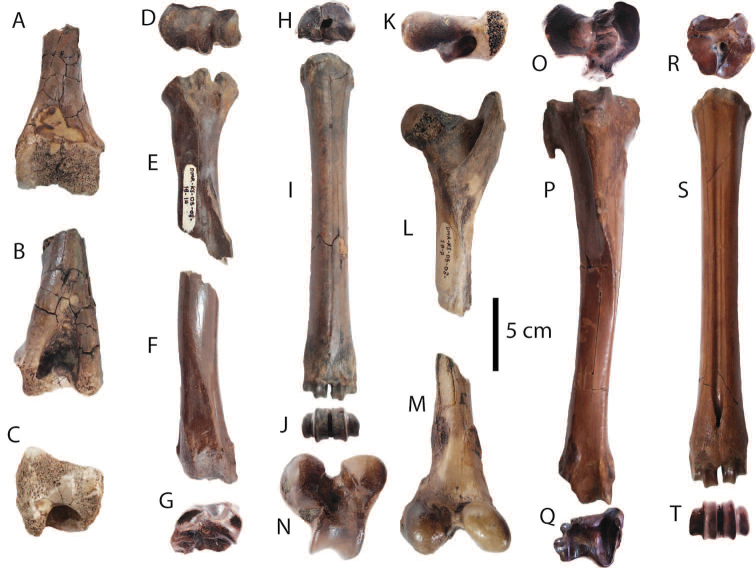
Postcranial remains of *Rusa unicolor* from Khok Sung: **A–C** DMR-KS-05-03-15-43, a left humerus in anterior (**A**), posterior (**B**), and distal (**C**) views **D–E** DMR-KS-05-03-19-14, a right proximal radius in proximal (**D**) and anterior (**E**) views **F–G** DMR-KS-05-03-26-19, a right distal radius in anterior (**F**) and distal (**G**) views **H–J** DMR-KS-05-03-17-26, a left metacarpus in proximal (**H**), anterior (**I**), and distal (**J**) views **K–L** DMR-KS-05-03-19-7, a right proximal femur in proximal (**K**) and anterior (**L**) views **M–N** DMR-KS-05-04-30-9, a left distal femur in posterior (**M**) and distal (**N**) views **O–Q** DMR-KS-05-03-28-16, a right tibia in proximal (**O**), anterior (**P**), and distal (**Q**) views **R–T** DMR-KS-05-03-19-11, a right metatarsus in proximal (**R**), anterior (**S**), and distal (**T**) views.

**Postcranial remains**: postcranial elements include a humerus (Fig. 24A–C), radii (Fig. 24D–G), a metacarpus (Fig. 24H–J), femora (Fig. 24K–N), a tibia (Fig. 24O–Q), and a metatarsus (Fig. 24R–T). All radii and femora are fragmentary. We assign these postcranial bones to *Rusa unicolor* according to the sized and proportional correlation with the extant specimens (Tab. 13 and Appendices 1 and 7–12).

######## Taxonomic remarks and comparisons.

According to Leslie (2011), we regard here *Rusa* as a separate genus within the family Cervidae. Four species are currently recognized: *Rusa unicolor* (sambar), *Rusa marianna* (Philippine deer), *Rusa timorensis* (rusa), and *Rusa alfredi* (Prince Alfred’s deer).

Antlers of *Rusa unicolor* are characterized by its typical three tines and forked beams at the tip, similar in shape to those of *Axis porcinus* but much more robust. The sambar deer shares a similar dental morphology with the Eld’s deer. But it differs from *Panolia eldii* as well as *Axis axis* in being larger-sized and in having more developed anterior cingulids on lower molars. The sambar deer is much larger than *Axis axis* (Figs 21 and 22). Based on the body mass estimated from the second molar sizes, Khok Sung large cervids fit well the size tendency of the modern populations of *Rusa unicolor* (Tab. 14). As demonstrated by the scatter diagrams (Figs 21 and 22), the recent sambar deer shows a wide range of size variation that sometimes overlaps with the Eld’s deer. The cheek teeth of Khok Sung *Rusa unicolor* conform to the size variability of their recent population. They are also comparable in size and morphology to the fossil sambar deer from Thum Prakai Phet (Filoux et al. 2015), Phnom Loang (Beden and Guérin 1973), and Ma U’Oi (Bacon et al. 2004) (Figs 21 and 22). As is the case for *Panolia eldii*, some cervid specimens described from Thum Wiman Nakin are improperly identified. For instance, the P2 (TF 3371 and TF 4570) probably do not belong to *Rusa unicolor* according to their smaller sizes. The taxonomic revision of fossil cervids from Thum Wiman Nakin would lead to the recognition of either higher or lower diversity of cervids in Southeast Asia during the Middle Pleistocene.

##### Family BOVIDAE Gray, 1821

###### Genus *Bos* Linnaeus, 1758

####### Bos sauveli

Taxon classificationAnimaliaArtiodactylaBovidae

Urbain, 1937

######## Referred material.

A left DP3, DMR-KS-05-03-29-8; a left P3, DMR-KS-05-04-01-4; a left fragmentary M1 or M2 (posterior portion), DMR-KS-05-03-23-2; a right M3, DMR-KS-05-03-29-6; a right mandible with m1–m3, DMR-KS-05-03-9-1; two left mandibles—DMR-KS-05-04-9-1 (with p2, p4, and m1–m3) and DMR-KS-05-04-29-1 (with m3); a left i2, DMR-KS-05-03-15-12; a right i3, DMR-KS-05-03-23-4; a right p2, DMR-KS-05-04-01-6; a right m1, DMR-KS-05-03-15-10; a right m2, DMR-KS-05-03-29-7; two m3—DMR-KS-05-04-28-4 (right broken posterior lobe) and DMR-KS-05-03-24-5 (left); a left humerus, DMR-KS-05-03-20-2(1).

######## Material description.

**Figure 25.**
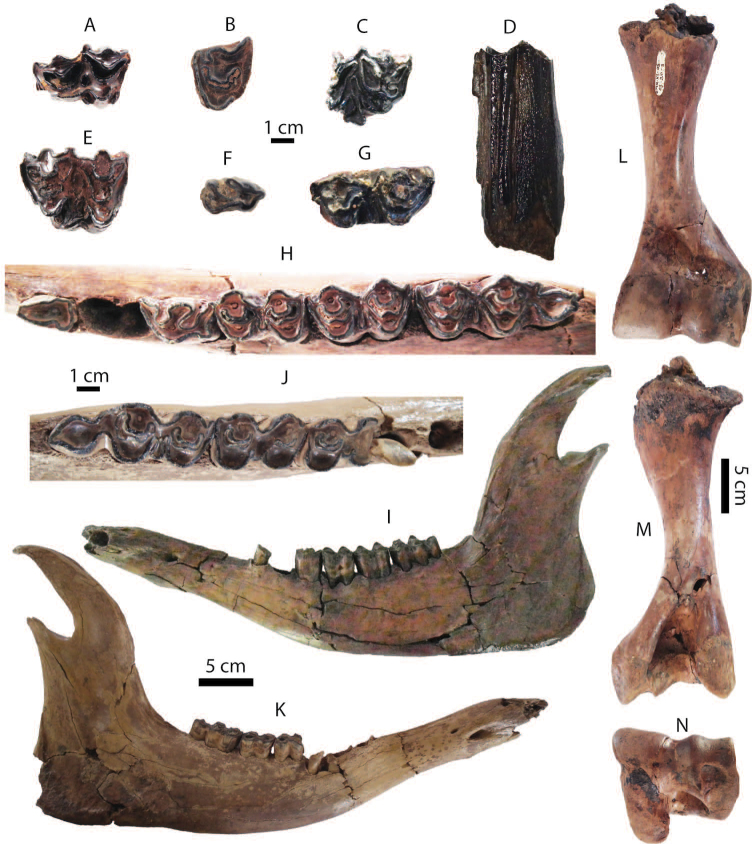
Remains of *Bos sauveli* from Khok Sung: **A** DMR-KS-05-03-29-8, a left DP3 **B** DMR-KS-05-04-01-4, a left P3 **C–D** DMR-KS-05-03-23-2, a left M1 or M2 in occlusal (**C**) and lingual (**D**) views **E** DMR-KS-05-03-29-6, a right M3 **F** DMR-KS-05-04-01-6, a right p2 **G** DMR-KS-05-04-28-4, a broken right m3 **H–I** DMR-KS-05-04-9-1, a left mandible in occlusal (**H**) and lateral (**I**) views **J–K** DMR-KS-05-03-9-1, a right mandible in occlusal (**J**) and lateral (**K**) views **L–N** DMR-KS-05-03-20-2(1), a left humerus in anterior (**L**), posterior (**M**), and distal (**N**) views. All isolated teeth are shown in occlusal view.

**Upper dentition**: DP3 (DMR-KS-05-03-29-8) is molariform and elongated, characterized by well-developed anterior and posterior cingula, buccal styles, and medial fossettes, a slightly-developed entostyle, and a reduction of the anterior lobe width and height compared to the posterior lobe (Fig. 25A). The P3 (DMR-KS-05-04-01-4) has distinct styles (particularly the metastyle), protocone, and hypocone and an irregular fossette. (Fig. 25B). Upper molars have a rectangular outline and distinct styles, entostyles, and single medial fossettes with wear (Fig. 25C, E) (for measurements, see Tab. 15). The infundibula are X- or metacentric chromosome-shaped on the moderately worn molars (Fig. 25C, E). The entostyles (column) of DMR-KS-05-03-23-2 (M1 or M2: Fig. 25C, D) and DMR-KS-05-03-29-6 (M3: Fig. 25E) are often bifurcated and lingually flat in occlusal view. A distinct longitudinal groove runs along the lingual surface of the entostyle (Fig. 25D). The M3 is more rectangular in outline compared to other upper molars. The posterior lobe of the M3 is relatively reduced in width and the fossettes are large (Fig. 25E).

**Table 15.** Measurements (lengths and widths in millimeters) of large bovid teeth from Khok Sung. N=number of specimens.

	Length			Width		
	N	Range	Mean	N	Range	Mean
***Bos sauveli***						
DP3	1	27.39	–	1	14.91	–
P3	1	17.57	–	1	19.71	–
M1 or M2	–	–	–	1	25.63	–
M3	1	35.46	–	1	23.55	–
i2	1	13.67	–	1	11.53	–
i3	1	13.68	–	1	8.68	–
p2	2	14.13–14.77	14.45	2	8.52–10.39	9.46
p4	1	23.39	–	1	12.91	–
m1	3	27.24–27.96	27.72	3	17.21–18.26	17.73
m2	3	29.70–32.47	30.11	3	17.87–18.79	18.29
m3	3	40.60–47.60	43.78	5	17.09–19.91	18.37
***Bos gaurus***						
DP2	1	22.28	–	1	10.67	–
P2	2	19.42–20.79	20.11	2	13.55–15.58	14.57
DP3	1	28.73	–	1	18.97	–
DP4	1	29.75	–	1	22.55	–
M1	1	26.33	–	1	29.95	–
M3	1	36.96	–	1	26.94	–
i1	1	20.30	–	1	11.35	–
p2	1	13.77	–	1	8.56	–
p3	1	21.58	–	1	11.92	–
p4	1	21.11	–	1	12.72	–
m1	2	25.29–28.67	26.98	2	18.25–19.28	18.77
m2	3	30.36–35.09	32.82	3	19.00–20.07	19.45
m3	2	42.56–46.23	44.40	2	18.72–18.79	18.76
***Bubalus arnee***						
P2	3	22.30–26.78	24.04	3	14.47–17.26	15.76
DP3	1	31.92	–	1	19.75	–
P3	7	17.85–25.03	21.58	7	15.56–21.93	20.32
DP4	1	31.60	–	1	23.36	–
P4	7	17.76–23.55	20.46	7	21.01–23.20	22.34
M1	9	25.73–33.16	28.61	8	26.01–29.79	27.30
M2	8	30.45–36.18	33.11	7	26.09–29.23	27.23
M3	6	33.74–37.40	36.07	6	25.26–27.30	26.29
i1	1	21.21	–	1	10.31	–
i2	1	16.17	–	1	11.94	–
i3	1	16.61	–	1	11.63	–
i4	1	15.82	–	1	8.80	–
p2	4	13.56–16.24	15.05	4	8.01–9.80	8.87
dp3	2	21.59–23.20	22.40	2	8.65–9.90	9.28
p3	3	21.88–23.09	22.30	3	10.23–13.09	11.80
dp4	3	37.25–42.59	40.74	3	13.34–15.24	14.39
p4	2	23.81–24.97	24.39	3	11.93–13.26	12.76
m1	9	30.49–36.77	32.66	6	17.67–20.36	18.94
m2	6	32.13–39.20	36.03	5	19.00–21.22	20.18
m3	3	46.52–48.33	47.29	4	17.64–20.72	19.66

**Mandible and lower dentition**: two mandibles, DMR-KS-05-03-9-1 (Fig. 25H, I) and DMR-KS-05-04-9-1 (Fig. 25J, K), are almost complete (for measurements, see Appendix 13). All incisors and premolars dropped out of the first specimen. The second specimen lacks all incisors and the p3. Another fragmentary mandible DMR-KS-05-04-29-1 preserves only a posterior lobe of the m3.

The i2 (DMR-KS-05-03-22-15) and i3 (DMR-KS-05-03-23-4) are spatulate and small, compared to other species of *Bos* (for measurements, see Tab. 15). The two p2 (DMR-KS-05-04-9-1: Fig. 25H and DMR-KS-05-04-01-6: Fig. 25F) is small and shows a protruding preprotoconulidcristid and a fusion between the postentocristid and the posthypocristid. The p4 displays well-developed conids and cristids. The postprotocristid is large, compared to other *Bos* species. On the lower molars, the metastylid is poorly-developed, but becoming more prominent in m3 (Fig. 25H). The anterior and posterior fossettes is metacentric chromosome-shaped with wear (Fig. 25H, J). The posterior talonid of the m3 is well-developed (Fig. 25H, J). The posthypoconulidcristid protrudes posteriorly and sometimes bifurcates into two flanges, as observed on the specimen DMR-KS-05-04-9-1 (Fig. 25H). The entostylid slightly protrudes lingually in relation to heavy wear and the posterior ectostylid is usually absent.

**Postcranial remains**: a humerus, DMR-KS-05-03-20-2(1), preserves the shaft and distal part (Fig. L–N). We attribute this humerus to *Bos sauveli* according to the proportional correlation with the extant specimens (Tab. 13 and Appendix 7). This specimen is also smaller than that of extant *Bos javanicus* and *Bos gaurus* (Appendices 1 and 7).

######## Taxonomic remarks and comparisons.

Southeast Asian large bovids are accurately identified by differences in cranial features (especially horn cores), although they show sexual and ontogenetic variation in morphology. Lacking the cranial remains, it is difficult to make a distinction within the species of *Bos*. Due to the lack of cranial remains of koupreys (*Bos sauveli*) collected from Khok Sung, we identify these fossils on the basis of dental features.

**Figure 26.**
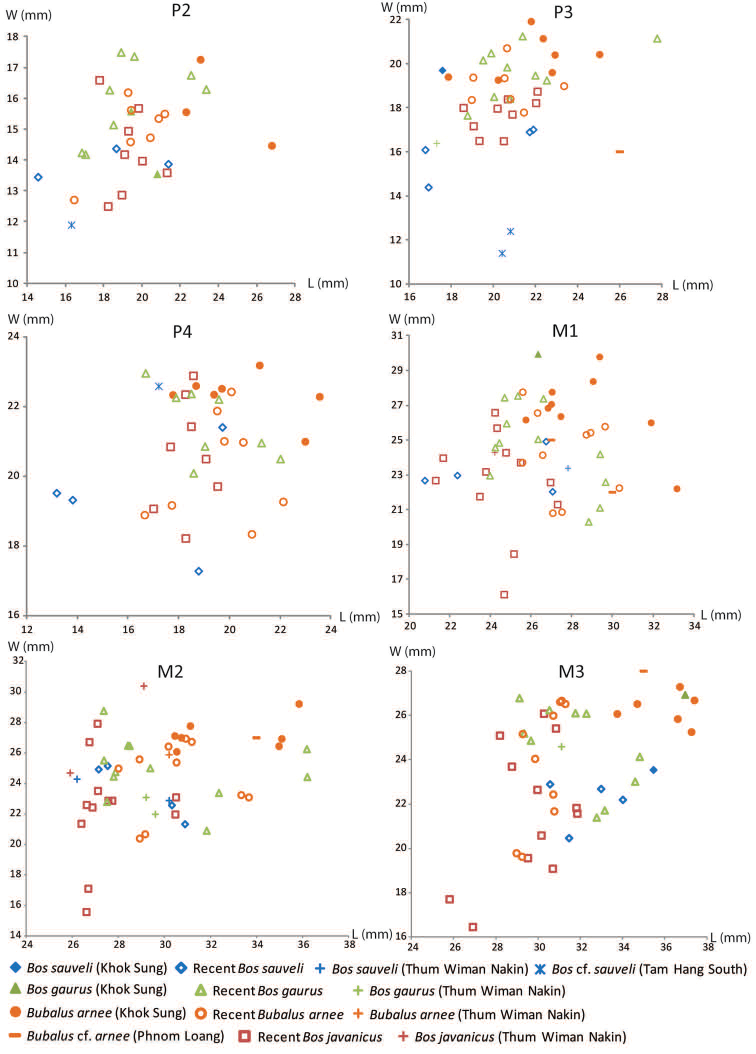
Scatter diagrams of upper cheek tooth (P2–M3) widths of recent and fossil large bovids. Fossil data from Phnom Loang, Lang Trang, Thum Wiman Nakin, and Tam Hang South are from Beden and Guérin (1973), de Vos and Long (1993), Tougard (1998), and Bacon et al. (2011), respectively.

**Figure 27.**
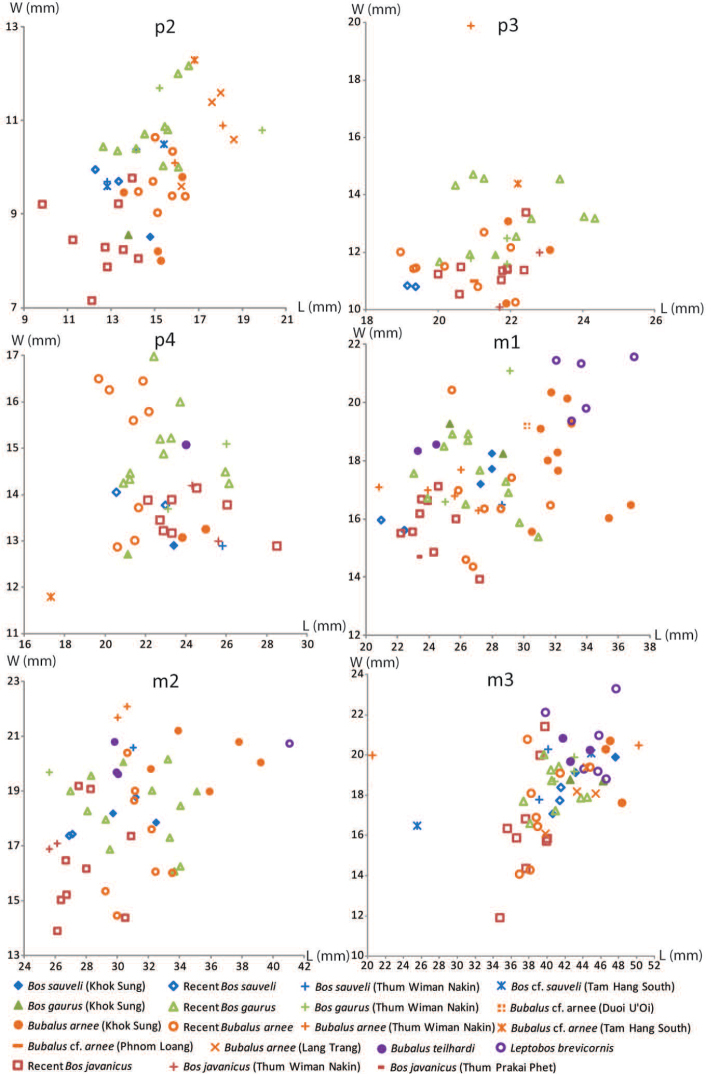
Scatter diagrams of lower cheek tooth (p2–m3) widths of recent and fossil large bovids. Fossil data from Phnom Loang, Lang Trang, Thum Wiman Nakin, Thum Prakai Phet, Duoi U’Oi, and Tam Hang South are from Beden and Guérin (1973), de Vos and Long (1993), Tougard (1998), Filoux et al. (2015), and Bacon et al. (2008b, 2011), respectively.

Based on our comparisons with some extant specimens (MNHN-ZMO-1940-51 and MNHN-ZMO-10801), the cheek teeth of koupreys are similar to those of other species of *Bos*, characterized by having hypsodont crowns, well-developed styles and stylids, a horse shoe-shaped infundibulum (anterior and posterior fossettes), and bifurcated or trifurcated entostyles depending on the wear stage. Among Southeast Asian large bovids, it differs from *Bos javanicus* (banteng) and *Bos gaurus* (gaur) in having a more developed postprotocristid on the p3 and p4, a metacentric chromosome-shaped molar in relation to the middle wear stage, a single large medial fossette on the upper molars, a flat lingual surface of the entostyle on the moderately to heavily worn molars. The M1 and M3 of *Bos sauveli* are almost more square and rectangular in outline, respectively, compared to those of other *Bos* species. *Bos sauveli* is usually smaller than *Bos gaurus* and *Bubalus arnee* (wild water buffalo), but is often comparable in size to *Bos javanicus* (Figs 26 and 27, and for the average of large bovid body mass, see Tab. 14).

According to the molecular phylogenetic analyses, the kouprey may have been domesticated in Cambodia (Hassanin et al. 2006) and they are probably a feral animal derived from hybridization between *Bos javanicus* and *Bos taurus indicus* (zebu) (Galbreath et al. 2006). However, the latter statement is not recently supported by the molecular sequences available for koupreys, bantengs, and zebus (Hassanin and Ropiquet 2007). These authors indicated that the mitochondrial sequences of Cambodian bantengs are divergent from those of Javan bantengs, but similar to those of koupreys. They also proposed that the mitochondrial genome of koupreys seems to have been transferred by natural hybridization into the ancestor of Cambodian bantengs. The taxonomic status of koupreys is currently under discussion and additional molecular analyses on Southeast Asian bantengs need to be examined in the future. However, our taxonomic identification of Khok Sung bovids suggests an existence of the Pleistocene kouprey in Thailand because of its high similarities in dental features with the type specimen MNHN-ZMO-1940-51 and the specimen MNHN-ZMO-10801.

####### Bos gaurus

Taxon classificationAnimaliaArtiodactylaBovidae

(Hamilton-Smith, 1827)

######## Referred material.

A left horn core, DMR-KS-05-03-26-22; a right DP2, DMR-KS-05-03-20-4; two right P2—DMR-KS-05-03-19-27 and DMR-KS-05-04-03-3; a right DP3, DMR-KS-05-03-20-3; a right DP4, DMR-KS-05-03-17-3; a right M1, DMR-KS-05-03-00-20; a right M3, DMR-KS-05-03-17-1; a right mandible with m1–m3, DMR-KS-05-03-00-1; a left mandible with p2–m3, DMR-KS-05-04-3-1; a left i1, DMR-KS-05-03-00-27; two left m2—DMR-KS-05-03-19-26 and DMR-KS-05-03-16-1; two humeri—DMR-KS-05-05-1-1 (right) and DMR-KS-05-03-00-62 (left); a right metacarpus, DMR-KS-05-03-26-27; two left femora—DMR-KS-05-03-9-2 and DMR-KS-05-04-30-1 (proximal part).

######## Material description.

**Figure 28.**
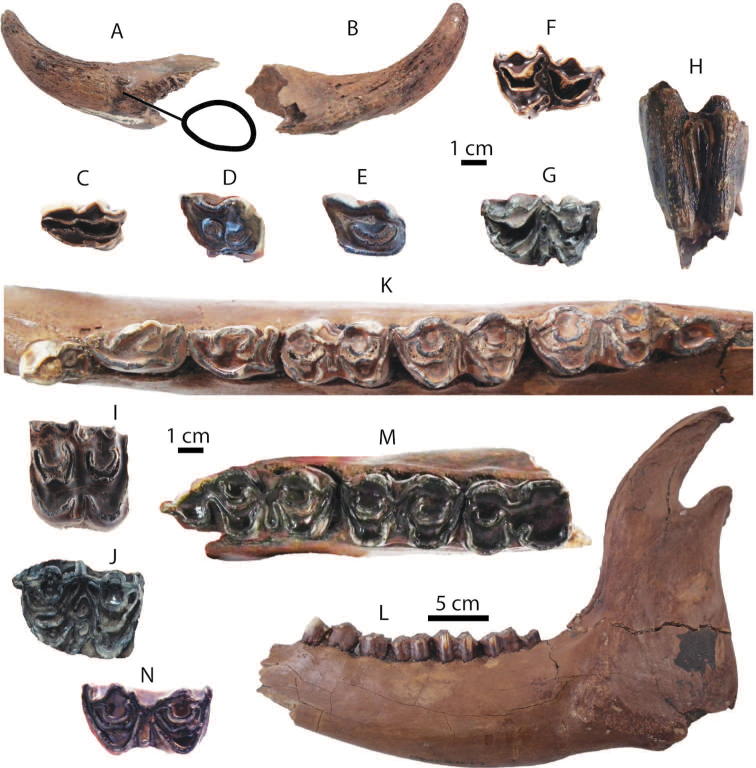
Remains of *Bos gaurus* from Khok Sung: **A–B** DMR-KS-05-03-26-22, a left horn core in posterior (**A**) and anterior (**B**) view **C** DMR-KS-05-03-20-1, a right DP2 **D** DMR-KS-05-03-19-27, a right P2 **E** DMR-KS-05-04-03-03, a right P2 **F** DMR-KS-05-03-20-3, a right DP3 **G–H** DMR-05-03-17-3, a right DP4 in occlusal (**G**) and lingual (**H**) views **I** DMR-05-03-17-1, a right M3 **J** DMR-05-03-19-26, a left m2 **K–L** DMR-KS-05-04-3-1, a left mandible in and occlusal (**K**) and lateral (**L**) views **M** DMR-KS-05-03-00-1, a fragmentary mandible in occlusal view. All isolated teeth are shown in occlusal view.

**Horn core**: a single horn core (DMR-KS-05-03-26-22) is small, curved upward (Fig. 28A, B) and slightly backward. The horn core base is oval in cross-section (Fig. 28A). A longitudinal ridge on the anterior surface of the horn core is present (Fig. 28B). This specimen belongs to a juvenile individual according to its very small size.

**Upper dentition**: DP2 (DMR-KS-05-03-20-4) is small and elongated, characterized by three main cones (anterior cone, paracone, and metacone) and a well-developed metastyle (Fig. 28C) (for measurements, see Tab. 15). The anterior and posterior fossettes fuse together. Two P2 (DMR-KS-05-03-19-27; Fig. 28D and DMR-KS-05-04-03-3: Fig. 28E) have a well developed paracone rib close to the parastyle and a nearly flat lingual wall. The fossettes are separated into two islands (larger for the anterior one) due to the heavy wear stage (Fig. 28D). The P2 shows a nearly straight posterior wall and is wider than the DP2 (Fig. 28E). On the molarized DP3, the posterior lobe is broader than the anterior lobe (Fig. 28F). A small medial fossette is present. The entostyle is short and projects posteriorly. The molarized DP4 (DMR-KS-05-03-17-3) is slightly worn, characterized by a rectangular outline, well-developed buccal styles, an unfused entostyle, and two separated medial fossette (Fig. 28G–H). The entostyle is bifurcated and situated between the protocone and hypocone (Fig. 28G). Two parallel longitudinal grooves are present along the lingual surface of the enstostyle, likely resulting in a trifurcated pattern in relation to the middle wear stage (Fig. 28H). The heavily worn M1 (DMR-KS-05-03-00-20) displays a subsquare outline and an unbifurcated entostyle positioned between the protocone and hypocone (Fig. 28I). The medial fossette is absent due to the heavy wear stage. The M3 (DMR-KS-05-03-17-1) exhibits well-developed buccal styles and large medial fossettes splitting into 2 islands with wear (Fig. 28J). The entostyle on the M3 is short, not bifurcated, and close to the hypocone.

**Mandibles and lower dentition**: DMR-KS-05-04-3-1 is complete, posterior to the p2, with the exception of a small part of the angular region (Fig. 28K, L) (for measurements, see Appendix 13). Another mandible (DMR-KS-05-03-00-1) preserves only a portion of the ramus with the complete molar row (Fig. 28M and Appendix 13). The isolated i1 (DMR-KS-05-03-00-27) is heavily worn, spatulate, and robust. Lower premolars have well-developed main cuspids and cristids (Fig. 28K, M). On the p2, the protocone is the highest cuspid and the posterior fossette is present. The p3 is elongated as long as the p4. The premetacristid is poorly developed. The postprotocristid on the p3 is larger than that on the p4. On the p4, the postprotocristid is narrow and anteroposteriorly constricted. The metaconid is most developed, compared to *Bos sauveli* and *Bos javanicus* as well as *Bubalus arnee*. For all lower molars, the ectostylid is slightly developed and not bifurcated (Fig. 28K, M–N) (for measurements, see Tab. 15). In lingual view, the metastylid is absent at the medium wear stage (Fig. 28K, M). In occlusal view, the entostylid is straight and short. The buccal outline of the protoconid and hypoconid is U-shaped in relation to the strong wear (Fig. 28M). The posterior talonid on the m3 is well-developed. The posthypoconulidcristid protrudes posteriorly.

**Figure 29.**
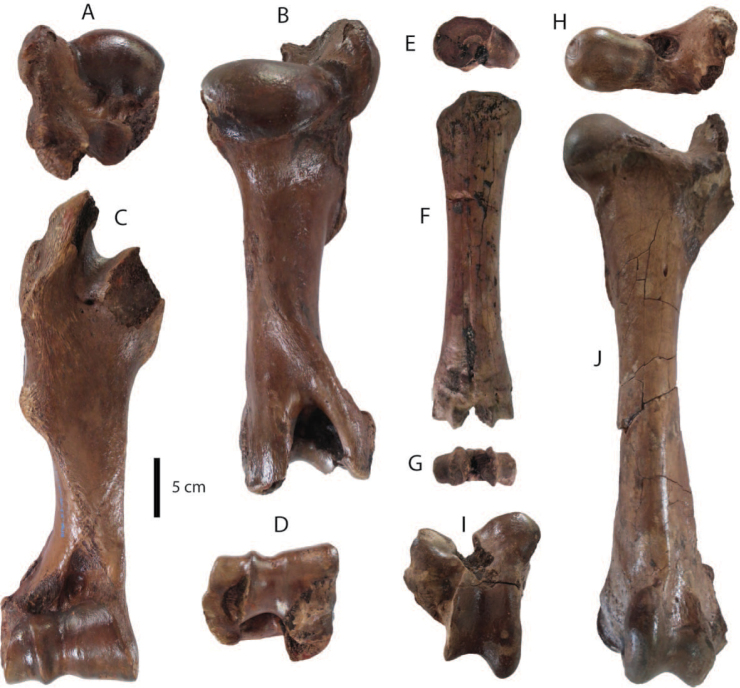
Postcranial remains of *Bos gaurus* from Khok Sung: **A–D** DMR-KS-05-05-1-1, a right humerus in proximal (**A**), posterior (**B**), anterior (**C**), and distal (**D**) views **E–G** DMR-KS-05-03-26-27, a right metacarpus in proximal (**E**), anterior (**F**), and distal (**G**) views **H–J** DMR-KS-05-03-9-2, a left femur in proximal (**H**), distal (**I**), and anterior (**J**) views.

**Postcranial remains**: postcranial elements include humeri (Fig. 29A–D), a metacarpus (Fig. 29E–G), and femora (Fig. 29H–J) (for measurements, see Appendix 1). The femur DMR-KS-05-04-30-1 lacks a distal portion. We assign these postcranial bones based on the proportional correlations with the recent specimens of *Bos gaurus* (Tab. 13 and Appendices 7 and 9–12).

######## Taxonomic remarks and comparisons.

According to IUCN (2015), the wild forms of gaurs are considered as *Bos gaurus*, while their domestic forms are recognized as *Bos frontalis* (Gentry et al. 2004). We consider here the Pleistocene fossil gaurs as belonging to wild forms in terms of taxonomic nomenclature.

We assign the juvenile horn core (DMR-KS-05-03-26-22) to *Bos gaurus* because the horn cores of gaurs are different from all other *Bos* species. They grow outward and curve upward, similar to those of *Bubalus arnee*, but their apical portion curves inward and slightly forward (Lekagul and McNeely 1988).

Mandibles and isolated teeth of *Bos gaurus* are also observed. The cheek teeth of *Bos gaurus* are distinguished from *Bos sauveli* and *Bos javanicus* by having two separate fossettes on the P2, more developed metaconids and more anteroposteriorly constricted postprotocristids on the p3 and p4, and more robust cheek teeth (Figs 26 and 27, and Tab. 15). The entostyles are usually bifurcated or sometimes trifurcated on the slightly to moderately worn upper molars (our observations on the comparative material of recent *Bos gaurus*: e.g., ZSM-1972-5 and ZSM-1961-313), similar to those of *Bos javanicus*. But the entostyle is not bifurcated, when the molar is extremely worn, as seen on the specimen DMR-KS-05-03-00-20 (Fig. 28I). This character is therefore morphologically variable through wear. On the m3, the entostylid and posterior talonid in *Bos gaurus* is almost more developed than that in *Bos javanicus*. The angle between the posthypocristid and prehypoconulidcristid is slightly more divergent in *Bos sauveli* than in *Bos gaurus*. The size of Khok Sung *Bos gaurus* falls within the range of the recent population (Figs 26 and 27, and Tab. 14). We elucidate here the co-occurrence of two *Bos* species, *Bos sauveli* and *Bos gaurus* (larger), in Khok Sung.

###### Genus *Bubalus* Hamilton-Smith, 1827

####### Bubalus arnee

Taxon classificationAnimaliaArtiodactylaBovidae

(Kerr, 1792)

######## Referred material.

A nearly complete cranium associated with a right mandible, DMR-KS-05-03-20-1; a cranium with a right tooth row (P3–M3), DMR-KS-05-03-21-1; a partial cranium with two tooth rows (P3–M1), DMR-KS-05-03-16-3; a partial cranium with a right tooth row (P3–M3), DMR-KS-05-03-11-1; three horn cores—DMR-KS-05-03-16-2 (right), DMR-KS-05-03-31-6 (right), and DMR-KS-05-03-19-28 (left); a left P2, DMR-KS-05-03-18-14; a left DP3, DMR-KS-05-03-00-103; two right P3—DMR-KS-05-03-22-14 and DMR-KS-05-04-05-3; a right DP4, DMR-KS-05-04-29-8 (broken anterior lobe); two P4—DMR-KS-05-03-18-13 (right) and DMR-KS-05-03-18-9 (left); four M1—DMR-KS-05-03-31-5 (right), DMR-KS-05-03-18-12 (right), DMR-KS-05-03-18-6 (left), and DMR-KS-05-03-22-13 (left); five M2—DMR-KS-05-03-00-2 (right), DMR-KS-05-03-25-21 (right), DMR-KS-05-03-18-5 (right), DMR-KS-05-03-16-2(1) (left), and DMR-KS-05-03-18-7 (left); four M3—DMR-KS-05-03-00-7 (right), DMR-KS-05-03-22-12 (left), DMR-KS-05-03-14-1 (left), and DMR-KS-05-03-18-10 (left); a right mandible with p2–m1, DMR-KS-05-03-20-2; three left mandibles—DMR-KS-05-03-10-3 (with p2–m3), DMR-KS-05-03-20-10 (with p2–m1), and DMR-KS-05-03-20-20 (with m1 and m2); a right i1, DMR-KS-05-03-18-8; a right i2, DMR-KS-05-03-22-15; a left i3, DMR-KS-05-03-00-106; a right i4, DMR-KS-05-03-16-3; a right p3, DMR-KS-05-03-14-4; a left dp4, DMR-KS-05-03-00-4; a right p4, DMR-KS-05-03-19-6; four m1—DMR-KS-05-03-25-3 (right), DMR-KS-05-03-18-18 (right), DMR-KS-05-03-00-105 (left), and DMR-KS-05-03-00-3 (left); two m2—DMR-KS-05-03-27-12 (right) and DMR-KS-05-03-25-2 (left); two m3—DMR-KS-05-03-18-11 and DMR-KS-05-04-29-2 (left posterior lobe); eleven thoracic vertebrae—DMR-KS-05-04-1-11 (T3), DMR-KS-05-04-1-26 (T4), DMR-KS-05-04-1-13 (T5), DMR-KS-05-04-1-14 (T6), DMR-KS-05-04-1-15 (T7), DMR-KS-05-04-1-16 (T8), DMR-KS-05-04-1-12 (T9), DMR-KS-05-04-1-17 (T10), DMR-KS-05-04-1-18 (T11), DMR-KS-05-04-1-19 (T12), and DMR-KS-05-04-1-20 (T13); four lumbar vertebrae—DMR-KS-05-04-1-24 (L1), DMR-KS-05-04-1-23 (L2), DMR-KS-05-04-1-22 (L3), and DMR-KS-05-04-1-21 (L4); two humeri—DMR-KS-05-03-31-1 (right) and DMR-KS-05-03-31-8 (left); two scapulae—DMR-KS-05-03-26-2 (right) and DMR-KS-05-02-20-4 (left); three ulnae and radii—DMR-KS-05-03-00-61 (right), DMR-KS-05-03-31-2 (right) and DMR-KS-05-03-31-9 (left); a right metacarpus, DMR-KS-05-03-26-3(1); a pelvis, DMR-KS-05-04-1-25; two femora—DMR-KS-05-04-1-1 (right) and DMR-KS-05-04-1-2 (left); a right fragmentary femur, DMR-KS-05-03-20-8 (distal part); three tibiae—DMR-KS-05-4-1-11 (right), DMR-KS-05-04-1-3 (left), and DMR-KS-05-03-20-9 (left); two fourth tarsal bones—DMR-KS-05-04-1-7 (right) and DMR-KS-05-04-1-5 (left); three metatarsi—DMR-KS-05-04-1-8 (right), DMR-KS-05-04-1-6 (left), and DMR-KS-05-03-28-30 (left); a left astragalus, DMR-KS-05-04-1-4; a left phalanx I, DMR-KS-05-04-1-9; a left phalanx II, DMR-KS-05-04-1-10.

######## Material description.

**Figure 30.**
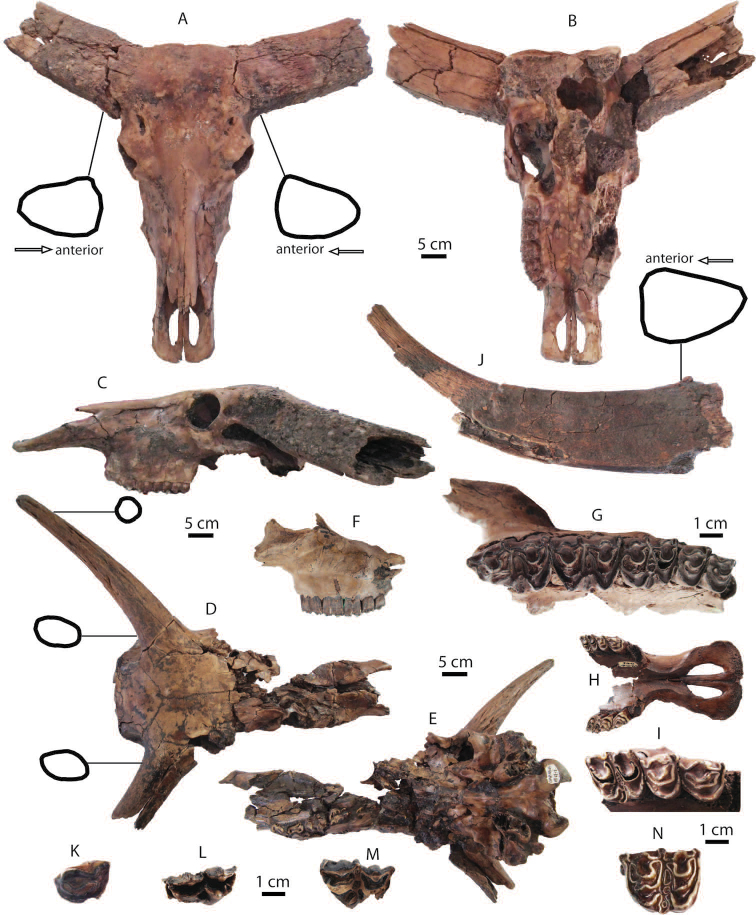
Cranial and upper dental remains of *Bubalus arnee* from Khok Sung: **A–C** DMR-KS-05-03-20-1, a cranium in dorsal (**A**), ventral (**B**), and lateral (**C**) views and **D–E** DMR-KS-05-03-21-1, a cranium in dorsal (**D**) and ventral (**E**) views **F–G** DMR-KS-05-03-11-1, a right upper jaw in lateral (**F**) and occlusal (**G**) views **H–I** DMR-KS-05-03-16-3, a partial cranium in ventral view (**H**) with a right tooth row (**I**) **J** DMR-KS-05-03-16-2, a right horn core in dorsal view **K** DMR-KS-05-03-18-14, a left P2 **L** DMR-KS-05-03-00-103, a left DP3 **M** DMR-KS-05-04-29-8, a right DP4 **N** DMR-KS-05-03-00-7, a right M3. Cross-sections of basal horn cores are given. All isolated teeth are shown in occlusal view.

**Crania and upper dentition**: DMR-KS-05-03-20-1 is undeformed and nearly complete (for measurements, see Appendix 14). Only the right maxilla, squamosals, and basicranium are damaged (Fig. 30A–C). The horn cores are broken at their middle portion. The cross-section of the horn core base is subtriangular and anteriorly flat (Fig. 30A). The frontals are narrow between the orbits and are flat or slightly convex at the region between horn core bases (Fig. 30A, C). The supraorbital foramina are large. The orbits face slightly forward (Fig. 30A, B), not laterally like *Leptobos brevicornis* and *Bubalus teilhardi* (Dong et al. 2014). The lateral margins of the premaxilla are concave (Fig. 30B).

DMR-KS-05-03-21-1, a juvenile cranium, is incomplete but slightly deformed. The posterior part of the skull is almost complete but the anterior part is broken (Fig. 30D, E). The cranium is likely elongated and laterally compressed (Fig. 30D). This specimen preserves two horn cores (broken at the right one) and a right tooth row with the M1, the P3 and P4 roots, and the unerupted M2 and M3 (Fig. 30E). The horn cores of DMR-KS-05-03-21-1 are slender, straight, and inclined upward and backward, and bend outward (Fig. 30D), similar to that of recent *Bubalus arnee* (e.g., MNHN-ZMO-1863-65). The horn cores are subtriangular in cross-section base, becoming subrounded toward the apex (Fig. 30D). The divergent angle between the horn cores is 105°. The frontals are short and narrow, forming an obtuse angle with the occipital plane. The parietals merged together. The occiput extends so far, posterior to the horn core bases. The basioccipital is laterally concave and triangular in outline (Fig. 30E).

DMR-KS-05-03-11-1 preserves the right zygomatic bone and the premaxilla and maxilla with a nearly complete tooth row (P3–M3) (Fig. 30F, G). Another specimen, DMR-KS-05-03-16-3, preserves the premaxilla and maxilla with P3–M1 (Fig. 30H, I). In dorsal and ventral views, the lateral margins of the premaxilla are concave, as expected for *Bubalus* (Fig. 30H).

Three isolated horn cores (DMR-KS-05-03-16-2: Fig. 30J, DMR-KS-05-03-31-6, and DMR-KS-05-03-19-28) are incomplete. The apical portion is broken away on each specimen. All horn cores are robust, long, and curved backward. Their anterior and dorsal surfaces are flat and their cross-sections are subtriangular at the base (Fig. 30J).

Upper cheek teeth of *Bubalus arnee* are more robust, compared to those of *Bos*. P2 (DMR-KS-05-03-18-14: Fig. 30K) is elongated. The parastyle on the P2 is less developed than that on the P3 and P4. The molarized DP3 (DMR-KS-05-03-00-103: Fig. 30L) is characterized by a well-developed buccal styles, anterior cingulum, entostyle, and spur, and a larger posterior lobe. The P3 is subtriangular in outline and is marked by a distinct parastyle, paracone rib, and metastyle and a U-shaped fossette (Fig. 30G, I). The parastyle of the P3 often curves posteriorly. The DP4 (DMR-KS-05-04-29-8: Fig. 30M) is also molarized with the broken protocone. This specimen has well-developed buccal styles and two separate medial fossettes. The entostyle curves posteriorly in occlusal view and is positioned more lingually than the protocone and hypocone. The P4 is similar in morphology to the P3, but is more anteroposteriorly compressed.

Upper molars display *Bos*-like patterns (e.g., the degree of the hypsodonty and selenodonty and the presence of distinct styles) but are more robust than most species of *Bos* (e.g., *Bos sauveli* and *Bos javanicus*) (Tab. 15). However, the mesostyles of upper molars of *Bubalus arnee* are more developed than those of *Bos*. The medial fossette between the anterior and posterior fossettes (infundibula) is well-developed, often separating into two or three islands with wear (Fig. 30G, I, N). The infundibula are U-shaped but sometimes become metacentric chromosome-shaped due to strong wear, like in *Bos sauveli* (Fig. 30G, N). In occlusal view, the entostyle is long and straight or curves posteriorly, depending on the stage of wear, but is never bifurcated (Fig. 30G, I, N). The small fossette is sometimes present within the entostyle in relation to strong wear (Fig. 30N).

**Figure 31.**
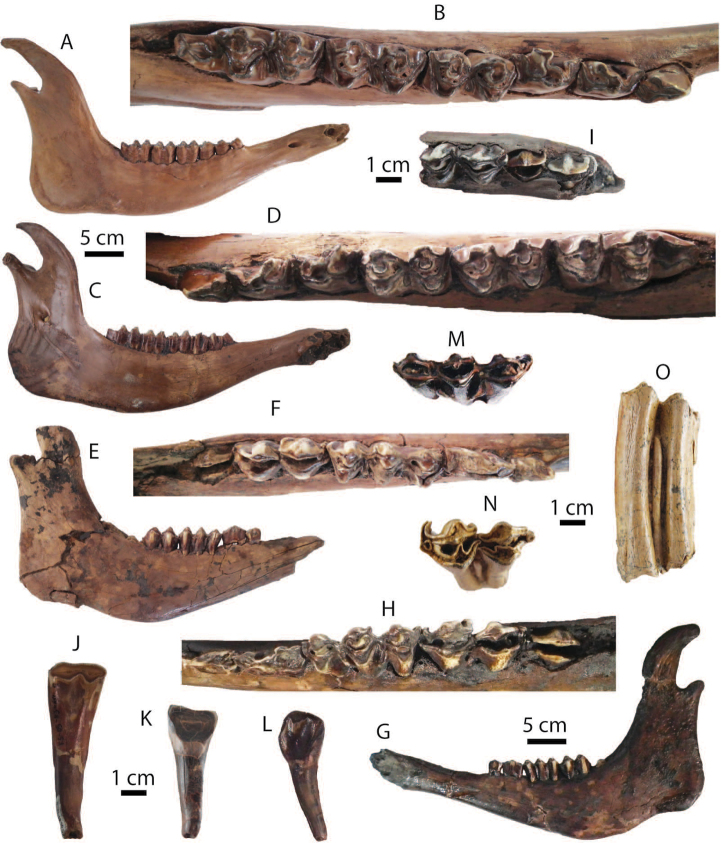
Mandibular and lower dental remains of *Bubalus arnee* from Khok Sung: **A–B** DMR-KS-05-03-20-1, a right mandible in lateral (**A**) and occlusal (**B**) views **C–D** DMR-KS-05-03-10-3, a left mandible in mesial (**C**) and occlusal (**D**) views **E–F** DMR-05-03-20-2, a right mandible in lateral (**E**) and occlusal (**F**) views **G–H** DMR-05-03-20-10, a left mandible in lateral (**G**) and occlusal (**H**) views **I** DMR-KS-05-03-20-20, a left fragmentary mandible with m1 and m2 in occlusal view **J** DMR-KS-05-03-18-8, a right i1 in lingual view; (**K**) DMR-KS-05-03-00-106, a left i3 in lingual view **L** DMR-KS-05-03-16-3, a right i4 **M** DMR-KS-05-03-00-4, a left dp4 in occlusal view **N–O** DMR-KS-05-03-00-105, a left m1 in occlusal (**N**) and buccal (**O**) views.

**Mandibles and lower dentition**: five mandibles: DMR-KS-05-03-20-1 (Fig. 31A, B), DMR-KS-05-03-10-3 (Fig. 31C, D), DMR-KS-05-03-20-2 (Fig. 31E, F), DMR-KS-05-03-20-10 (Fig. 31G, H), and DMR-KS-05-03-20-20 (Fig. 31I), are almost complete (for measurements, see Appendix 13). The first specimen is associated with the cranium. The right specimen DMR-KS-05-03-20-2 and the left specimen DMR-KS-05-03-20-20 belong to the same individual, bearing p2, dp3, dp4, and an unerupted m2. The left one is very fragmentary. Another mandible DMR-KS-05-03-20-10 is nearly complete, preserving the mandibular symphysis and bearing an unerupted m2, but lacking all incisors. All incisors drop out of the mandibles. The isolated lower incisors are spatulate in shape (Fig. 31J–L). The i2 is similar in size to the i3 (Tab. 15).

All lower cheek teeth are robust. All lingual stylids are distinct. The p2 has a well-developed postentocristid and posthypocristid (Fig. 31B, D, F, H). The metaconid is positioned more lingually than all of lingual cristids. The dp3 is elongated (Fig. 31F, H). The postprotocristid is large and the metaconid is well-developed. A small anterior fossette is present with wear. The p3 displays a well-developed preprotoconulidcristid and a posteriorly bending metaconid (Fig. 31B, D). The isolated dp4 (DMR-KS-05-03-00-4: Fig. 31M) is trilobed and elongated with a well-developed stylids (anterior and posterior ectostylid, parastylid, metastylid, and entostylid. On the dp4, the buccal outline of the protoconulid, protoconid, and hypoconid is V-shaped in occlusal view (Fig. 31F, H, M). The anterior ectostylid curves slightly posteriorly in contrast to the posterior ectostylid that bends anteriorly (Fig. 31M). A large fossette is present between the medial and posterior valley in relation to middle wear stage (Fig. 31M). On the p4, the metaconid is most lingually positioned (Fig. 31B, D). The premetacristid is more developed than the postmetacristids. The postprotocristid is very anteroposteriorly constricted. The postentocristid fuses with the posthypocristid beyond the middle stage of wear.

Lower molars have well-developed stylids and conids. The metastylid is most developed on the unworn to slightly worn specimens (Fig. 31F, H, I, N and Tab. 15). The metastylid is located closely to the metaconid. In occlusal view, the anterior and posterior fossettes are U-shaped, similar to that of *Bos*. The entostylid is well-developed and sometimes curves anteriorly (Fig. 31F, I). On the m3, the posterior ectostylid is absent. The posthypoconulidcristid protrudes posteriorly slightly and is sometimes bifurcated (Fig. 31B, D). The back fossette is sometimes present with wear.

**Figure 32.**
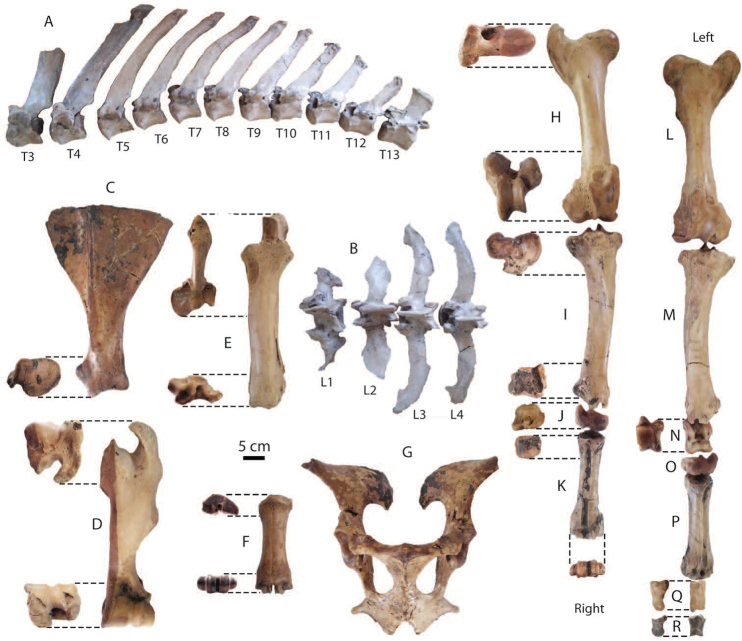
Articulated postcranial skeletons of *Bubalus arnee* from Khok Sung: **A** thoracic (abbreviated as “T”) vertebrae in lateral view: DMR-KS-05-04-1-11 (T3), DMR-KS-05-04-1-26 (T4), DMR-KS-05-04-1-13 (T5), DMR-KS-05-04-1-14 (T6), DMR-KS-05-04-1-15 (T7), DMR-KS-05-04-1-16 (T8), DMR-KS-05-04-1-12 (T9), DMR-KS-05-04-1-17 (T10), DMR-KS-05-04-1-18 (T11), DMR-KS-05-04-1-19 (T12), and DMR-KS-05-04-1-20, (T13) **B** lumbar (L) vertebrae in dorsal view: DMR-KS-05-04-1-24 (L1), DMR-KS-05-04-1-23 (L2), DMR-KS-05-04-1-22 (L3), and DMR-KS-05-04-1-21 (L4) **C–E** a left forelimb in anterior view: (**C**) DMR-KS-05-02-20-4, a scapula in lateral and distal views **D** DMR-KS-05-03-31-8, a humerus in proximal and distal views **E** DMR-KS-05-03-31-9, an ulna and a radius in proximal and distal views **F** DMR-KS-05-03-26-3(1), a right metacarpus in proximal, anterior, and distal views **G** DMR-KS-05-04-1-25, a pelvis in ventral view **H–R** hindlimbs in anterior view: **H** DMR-KS-05-04-1-1, a right femur in proximal and distal views **I** DMR-KS-05-4-1-11, a right tibia in proximal and distal views; (**J**) DMR-KS-05-04-1-7, a right 4^th^ tarsal bone in dorsal view **K** DMR-KS-05-04-1-8, a right metatarsus in proximal and distal views **L** DMR-KS-05-04-1-2, a left femur **M** DMR-KS-05-04-1-3, a left tibia **N** DMR-KS-05-04-1-4, a left astragalus in plantar view **O** DMR-KS-05-04-1-5, a left 4^th^ tarsal bone **P** DMR-KS-05-04-1-6, a left metatarsus **Q** DMR-KS-05-04-1-9, a left phalanx I in lateral view **R** DMR-KS-05-04-1-10, a left phalanx II in lateral view.

**Postcranial remains**: postcranial elements include scapulae (Fig. 32C), humeri (Fig. 32D), ulnae and radii (Fig. 32E), femora (Fig. 32H, L), tibiae (Fig. 32I, M), fourth tarsal bones (Fig. 32O), metacarpi (Fig. 32F), metatarsi (Fig. 32K, P), phalanges (Fig. 32Q, R), a pelvis (Fig. 32G), and thoracic and lumbar vertebrae (Fig. 32A, B). Most of postcranial remains belong to the same individual because they were found in connection. But some isolated specimens (scapula: DMR-KS-05-03-26-2, ulna and radius: DMR-KS-05-03-00-61, femur: DMR-KS-05-03-20-8, and metatarsus: DMR-KS-05-03-28-30) were found separately. The articulated skeletons show a typical character of *Bubalus arnee* whose postcranial bones are more massive and thicker than those of *Bos* (Fig. 32 and Appendix 1).

######## Taxonomic remarks and comparisons.

According to IUCN (2015), the wild forms of water buffaloes are considered as *Bubalus arnee*, while their domestic forms are regarded as *Bubalus bubalis* (Gentry et al. 2004).

Although the cheek teeth of *Bos* and *Bubalus* are almost morphologically identical and often show highly variable occlusal morphologies in relation to the wear stages, they are distinguishable based on the dental morphology. Bacon et al. (2011) mentioned that *Bubalus arnee* is distinguished from *Bos* by several dental characters: more massive and voluminous cones, conids, and lingual stylids, more complex patterns of folded infundibula on the upper molars, U-shaped protoconids and hypoconids on the lower molars, and unbilobed entostyles and ectostylids. However, the latter two characters are highly variable with wear, as observed on many extant specimens of *Bubalus arnee* from MNHN, ZSM, and THNHM. Among the modern large bovids in Southeast Asia, some lower premolar (p3 and p4) and third molar features are more informative for the species identification than others (Thein 1974). Our comparisons suggest that the cheek teeth of *Bubalus arnee* differ from those of *Bos* in having more developed mesostyles, more complex shapes of the infundibulum at the similar stages of wear, less developed or smaller metaconids and narrower postprotocristids on the p3 and p4, a presence of the small fossette within the entostyle and an absence of the longitudinal groove on the lingual surface of the entostyle on upper molars, more distinct entostylids on the m3, and a presence of the back fossette on the m3. For the incisors, it is difficult to make a morphological distinction between *Bubalus* and *Bos*. However, we assign these isolated lower incisors to *Bubalus arnee* because they were found together with their molars at the same spot.

As demonstrated by the scatter diagrams (Figs 26 and 27), the cheek teeth of recent *Bos* and *Bubalus* populations are highly overlapping in size. The lower molar sizes of *Bubalus arnee* also overlap with some fossil species (*Bubalus teilhardi* and *Leptobos brevicornis*). However, tooth dimensions are informative to make an ongoing distinction among the Khok Sung large bovids. The largest bovid in this locality is *Bubalus arnee*, followed by *Bos gaurus* and *Bos sauveli*, respectively, similar to the size tendency of their recent population (Tab. 14).

###### Genus *Capricornis* Ogilby, 1836

####### Capricornis sumatraensis

Taxon classificationAnimaliaArtiodactylaBovidae

(Bechstein, 1799)

######## Referred material.

A left M2, DMR-KS-05-03-18-16; three m3—DMR-KS-05-04-05-4 (right), DMR-KS-05-03-27-5 (left), and DMR-KS-05-03-28-10 (left posterior fragment).

######## Material description.

**Figure 33.**
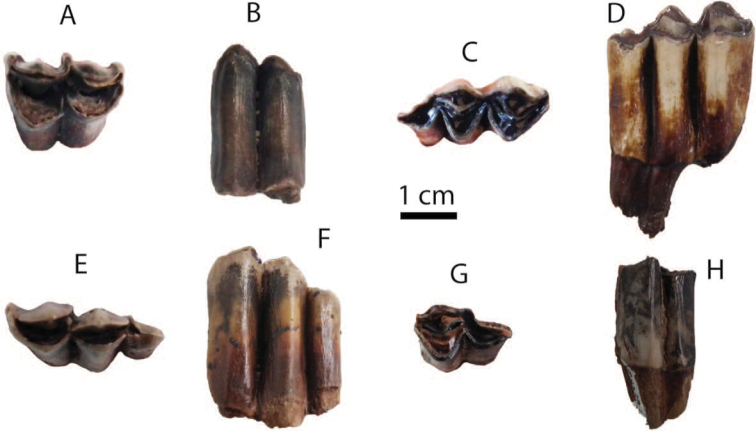
Dental remains of *Capricornis sumatraensis* from Khok Sung: **A–B** DMR-KS-05-03-18-16, a left M2 in occlusal (**A**) and lingual (**B**) views **C–D** DMR-KS-05-04-05-4, a right m3 in occlusal (**C**) and buccal (**D**) views **E–F** DMR-KS-05-03-27-5, a left m3 in occlusal (**E**) and buccal (**F**) views **G–H** DMR-KS-05-03-28-10 in occlusal (**G**) and buccal (**H**) views.

Isolated teeth are almost complete (for measurements, see Tab. 16), with the exception of the specimen DMR-KS-05-03-28-10 that preserves only a posterior lobe (Fig. 33G). Molars show typical features of *Capricornis* characterized by hyposodont crowns, smooth enamel, and distinct styles and stylids, and an absence of the ectostylids (Fig. 33). The parastyle, mesostyle, and metastyle on the M2 are perpendicular to the buccal wall (Fig. 33A). On the m3, the mesostylid is more developed than the other stylids and the posthypoconulidcristid protrudes posteriorly (Fig. 33C, E).

**Table 16.** Measurements (lengths and widths in millimeters) of molars of Khok Sung *Capricornis sumatraensis*.

**Specimen**		**Length**	**Width**
DMR-KS-05-03-18-16	M2	17.02	15.62
DMR-KS-05-03-28-10	m3	–	10.72
DMR-KS-05-03-27-5	m3	23.94	9.94
DMR-KS-05-04-05-4	m3	21.99	9.52

######## Taxonomic remarks and comparisons.

**Figure 34.**
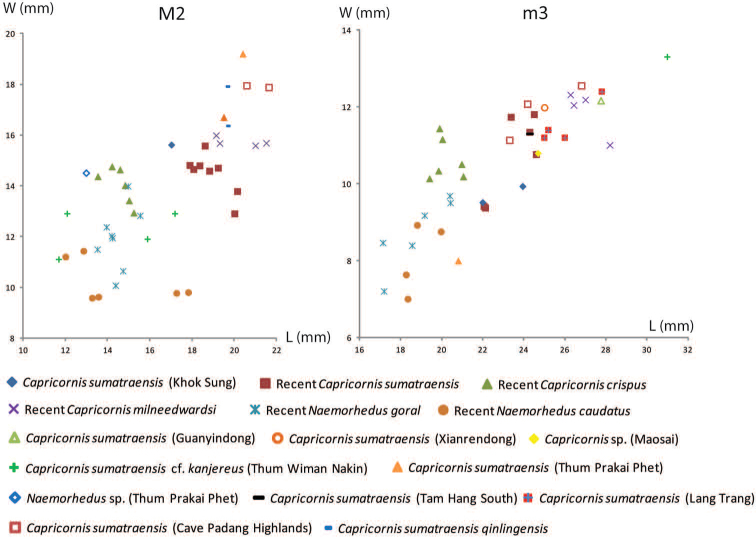
Scatter diagrams of M2 and m3 lengths and widths of recent and fossil serows and gorals. The measurements of fossil specimens from Lang Trang, Thum Wiman Nakin, Thum Prakai Phet, and Tam Hang South are from de Vos and Long (1993), Tougard (1998), Filoux et al. (2015), and Bacon et al. (2011), respectively.

We assign these isolated teeth from Khok Sung to *Capricornis sumatraensis* (Sumatran serow) because they are comparable in size and morphology to the extant specimens (Fig. 34). Among congeneric species, *Capricornis sumatraensis* is larger than *Capricornis crispus* as well as two goral species (*Naemorhedus goral* and *Naemorhedus caudatus*), but is smaller than *Capricornis milneedwardsi*. In addition, it differs from *Capricornis crispus* in having more developed metastylid and entostylid and a presence of back fossettes on the slightly worn m3 and from *Capricornis milneedwardsi* in having less developed metastylid and posthypoconulidcristid on the m3.

Compared to other fossil records, *Capricornis sumatraensis* from Khok Sung is smaller than that from the Late Pleistocene of Lang Trang in Vietnam (de Vos and Long 1993), Tam Hang South in Laos (Bacon et al. 2011), Padang Cave in Sumatra (Hooijer 1958), and Xianrendong in China (Chen and Qi 1978, Chen and Li 1994) (Fig. 34) and from the late Middle Pleistocene of Guanyindong (Li and Wen 1986) in China. The Khok Sung material also matches morphologically that of the subspecies *Capricornis sumatraensis kanjereus* from the Middle Pleistocene of Yenchingkuo in China (Colbert and Hooijer 1953) and from the late Middle Pleistocene of Thum Wiman Nakin in Thailand (Tougard 1998). However, *Capricornis sumatraensis* from Khok Sung is larger than that from Thum Wiman Nakin and *Naemorhedus* from Thum Prakai Phet. It differs from *Capricornis sumatraensis qinlingensis* described from the middle Early Pleistocene of Gongwangling in northern China (Hu and Qi 1978, Zhu et al. 2015) in having its smaller size and less developed parastyle and metastyle on the M2. However, we do not assign the material to the subspecies level based on the few isolated teeth.

### Class REPTILIA Laurenti, 1768

#### Order CROCODILIA Owen, 1842

##### Family CROCODYLIDAE Laurenti, 1768

###### Genus *Crocodylus* Laurenti, 1768

####### Crocodylus cf. siamensis

Taxon classificationAnimaliaCrocodyliaCrocodylidae

Schneider, 1801

######## Referred material.

A fragmentary cranium, DMR-KS-05-03-30-30; a dentary fragment with one tooth, DMR-KS-05-03-21-1; five isolated teeth—DMR-KS-05-03-00-19, DMR-KS-05-03-14-3, DMR-KS-05-03-22-22, DMR-KS-05-04-06-3, and DMR-KS-05-04-29-10; three osteoderms—DMR-KS-05-03-29-57, DMR-KS-05-03-29-58, and DMR-KS-05-03-27-25.

######## Material description.

**Figure 35.**
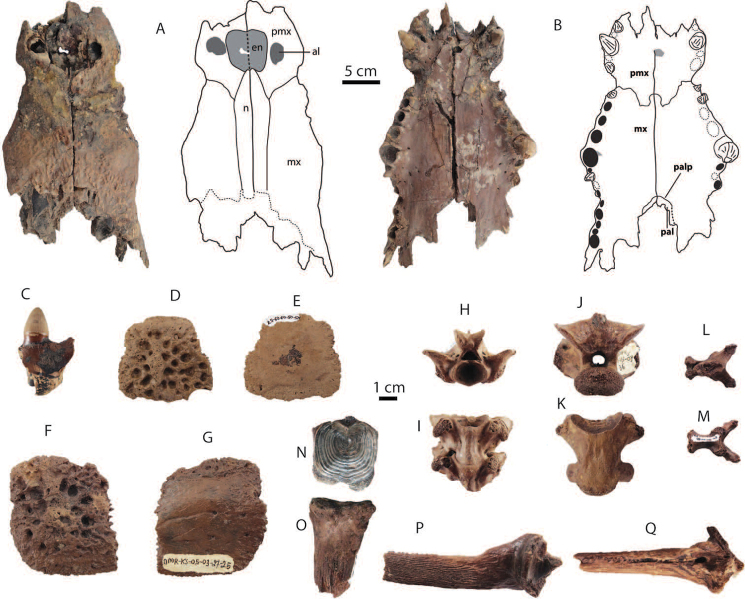
Remains of non-mammalian vertebrates from Khok Sung: Crocodylus cf. siamensis— **A–B** DMR-KS-05-03-30-30, a cranium in dorsal (**A**) and ventral (**B**) views **C** DMR-KS-05-03-21-1, a tooth in lingual view **D–E** DMR-KS-05-03-29-57 and **F–G** DMR-KS-05-03-27-25, osteoderms in dorsal (**D, F**) and ventral (**E, G**) views; *Python* sp.— **H–I** DMR-KS-05-03-00-16, a trunk vertebra in anterior (**H**) and ventral (**I**) views; *Varanus* sp. **J–K** DMR-KS-05-03-08-36, a trunk vertebra in anterior (**J**) and ventral (**K**) views; Galliformes indet.— **L–M** DMR-KS-05-04-05-40, a cervical vertebra fragment in dorsal (**L**) and ventral (**M**) views; Siluridae indet.— **N–O** DMR-KS-05-03-22-76, a vertebra in anterior (**N**) and lateral (**O**) views **P** DMR-KS-05-04-11-20, a pectoral spine in dorsal view **Q** DMR-KS-05-04-05-25, a pectoral spine in medial view. Anatomical abbreviations: **al**, alveolus; **pmx**, premaxilla; **en**, external naris; **n**, nasal; **mx**, maxilla; **pal**, palatine; **palp**, palatine process.

**Skull and dentition**: DMR-KS-05-03-30-30 is a slightly deformed skull preserving a nearly complete premaxilla, maxilla, nasal, and palatine process (Fig. 35A, B), and a partial palatine at the ventral part. The minimum length of the skull is 315 mm. The external naris is wide, dorsally directed, and presumably subcircular in outline (Fig. 35A). The nasal becomes narrower at the nearly premaxillary-maxillary suture and tapers into a point at the posterior rim of the naris. The premaxilla is broken anteriorly at the hole for the reception of the first dentary alveolus. The premaxilla contains at least four teeth on each side. The second one is the largest tooth in the premaxillary rows, regularly corresponding to the position of a large alveolar hole in dorsal view. A short premaxillary process extends to the second maxillary alveolus centrally or the first interalveolus laterally in ventral view (Fig. 35B). The premaxillary–maxillary suture is characterized by distinct notches. A maxilla comprises 14 alveoli, with the largest tooth crown (44.3 mm high) positioned at the fifth dentary alveolus. The width of the skull at the fifth maxillary tooth is 171.8 mm (the maximum width of the preserved skull). The width of the skull at the diastema between the last premaxillary tooth and the first maxillary tooth (the minimum width of the preserved skull) is 98.9 mm. Many small foramina in front of the alveoli are situated on both the premaxilla and the maxilla. Along the anterior to posterior maxillary rims, the tooth row is slightly convex until ending at the eighth or ninth alveolus. Teeth are characterized by their conical forms and striated surfaces. However, they are highly variable in shape and size, in relation to the position along the tooth row. The teeth of crocodyles are either slender and pointed or short and blunt (Fig. 35C) but much more massive than those of gharials. Asymmetrical surfaces of the tooth are divided by two prominent longitudinal ridges that are positioned anteriorly and posteriorly.

**Osteoderms**: two nearly complete specimens (Fig. 35D–G) and one small fragment are characterized by rectangular shapes, wider than long (about 5–6 cm long and 7–8 cm width), and slightly flat to convex and irregular edges with small spiny outgrowths. A short median keel does not extend far anteriorly or posteriorly (Fig. 35D, F). The external surface has several large and rounded to elliptical pits on the dorsal part and fewer small foramina and striae with surrounding fibrous patterns on the ventral part (Fig. 35E, G). These specimens differ from Gavialis cf. bengawanicus (Martin et al. 2012) in the same locality by their more ornamented pits and more irregular surfaces on the dorsal surface.

######## Taxonomic remarks and comparisons.

The specimen DMR-KS-05-03-30-30 is a crocodilian cranium with a possible maximum length up to 50 cm. All morphological characters of the Khok Sung crocodiles are congruent with the extant fresh water crocodile, *Crocodylus siamensis*, as well as with its fossils recovered from the Early and Middle Pleistocene of Java (Trinil H. K., Kedung Brubus, and Kedung Lumbu) (Delfino and de Vos 2010). However, the Khok Sung cranium preserves only the anterior midway portion of the skull and does not allow some morphological access to other important parts (e.g., lacrymals, jugals, and pterygoids). We thus attribute this material to Crocodylus cf. siamensis.

#### Order SQUAMATA Oppel, 1811

##### Suborder SERPENTES Linnaeus, 1758

###### Family BOIDAE Gray, 1825

####### Genus *Python* Daudin, 1803

######## Python sp.

Taxon classificationAnimaliaSquamataBoidae

######### Referred material.

Four trunk vertebrae—DMR-KS-05-03-00-21, DMR-KS-05-03-00-16 (two attached vertebrae), and DMR-KS-05-04-28-12.

######### Material description.

Vertebrae are almost complete and represent a large-sized snake (for measurements, see Tab. 17). In anterior view, the cotyle is suboval in outline with the dorsoventral compression (Fig. 35H). The ventro-lateral margins of the cotyle are nearly straight. The neural spine is well-developed and steep. The neural canal is narrow. The dorsal margin of the zygosphene is convex. The tubercle is located at the junction between the base of the zygoshene and the top of the neural canal. In posterior view, the neural arch is high and massive. The zygantra are wide and deep. In dorsal view, the median tubercle at the base of the zygosphene is distinct and the interzygapophyseal constriction is well-developed. In ventral view, the haemal keel is high (Fig. 35I) and the subcentral groove is poorly developed.

**Table 17.** Measurements (in millimeters) of vertebrae of *Python* and *Varanus* from Khok Sung. Abbreviations: CL, centrum length (measured at the ventral midline); **H**, maximum height (measured from the tip of the neural spine to the ventral rim of the cotyle); WPP, width between pre- and postzygapophyseal processes; Wpre, width across zygapophyseal processes; Wpost, width across postzygapophyseal processes; Wcd, width of the condyle; Hcd, height of the condyle (measured from the dorsal to ventral rim); Wct, width of the cotyle; Hct, height of the cotyle. ^+^ refers to the measurement of two attached vertebrae and * indicates an incomplete preservation.

	CL	H	WPP	Wpre	Wpost	WCd	Hcd	WCd	Hct
***Python* sp.**									
DMR-KS-05-03-00-21	20.85	40.36	22.47	36.48	15.27	14.03	13.95	12.87	15.12
DMR-KS-05-03-00-16	28.75^+^	26.35*	23.82	35.23	13.73	–	12.04	–	16.44
DMR-KS-05-04-28-12	14.06	17.69*	17.18	20.46	23.64	6.50	6.80	6.62	7.82
***Varanus* sp.**									
DMR-KS-05-03-29-36	24.98	25.39*	27.90	21.91	34.98	7.18	9.21	18.96	22.09
DMR-KS-05-03-08-36	31.73	28.56	34.11	36.21	35.60	7.82	12.68	18.27	21.91

######### Taxonomic remarks and comparisons.

These four vertebrae are attributed to the family Boidae because of the following characters: a short, wide, and massive vertebral body (i.e., the widths of the centra are greater than the lengths, sensu Delfino et al. (2004)), a small prezygapophyseal process, paradiapophyses weakly subdivided into para- and diapophyseal surfaces, and an absence of spine-like hypapophyses on mid- and posterior-trunk vertebrae (replaced by haemal keels) (Szyndlar and Böhme 1996, Rage 2001). Vertebrae of pythonines are commonly identified by many distinct characters: a straight and posteromedially angled zygapophyseal bridge, a triangular-shaped neural canal, a prominent zygosphenal tuberosity, a steep anterior border of the neural spine, a posterior border of the neural spine overhanging posteriorly, an absence of the paracotylar foramina, a haemal keel of mid- and posterior-trunk vertebrae delimited laterally by subcentral grooves that reach the cotylar rim, and a haemal keel projecting below the centrum (Scanlon and Markness 2001, Szyndlar and Rage 2003). The Khok Sung snake vertebrae are identified based on overall similarities with extant taxa (from the original description by Hoffstetter (1964)): a relatively elongated centrum compared to the neural arch width and the vertebral height, a longitudinal ridge along the haemal keel, and a thick zygosphenal base. The Khok Sung specimens are comparable in size to recent (e.g., *Python molurus bivittatus*: the specimen NMW 17117) and fossil (e.g., *Python* sp.: the specimens RMNH DUB 5794, DUB 6951, and DUB 6952 recovered from Trinil H. K., Java) python vertebrae. According to the fact that the species-level distinction based on the vertebral morphology is poorly known, we therefore assign these vertebrae to *Python* sp.

##### Suborder LACERTILIA Günther, 1867

###### Family VARANIDAE Merrem, 1820

####### Genus *Varanus* Merrem, 1820

######## Varanus sp.

Taxon classificationAnimaliaSquamataVaranidae

######### Referred material.

Two trunk vertebrae—DMR-KS-05-03-08-36 and DMR-KS-05-03-29-36.

######### Material description.

The vertebra DMR-KS-05-03-08-36 is more complete than the specimen DMR-KS-05-03-29-36 (for measurements, see Tab. 17). The pre- and postzygapophyses are slightly broken at the second specimen. In both specimens, the neural spines are unfortunately broken away. In anterior view, the cotyle is oval in outline, dorsoventrally compressed, and ventrally oriented (Fig. 35J). The prezygapophyses lack a part of the prezygapophyseal process and are dorsally inclined about 45°. The neural canal is narrow. The neural arch lacks a part of the zygosphene. No paracotylar foramina are present. In posterior view, the condyle and the postzygapophyses show a mirrored morphology with the anterior part. No zygantrum is observed. In dorsal view, the prezygapophyseal facets are drop-shaped and project laterally. The interzygapophyseal constriction is also present. In ventral view, the synapophyses protrude laterally and the centrum is triangular in outline (Fig. 35K).

######### Taxonomic remarks and comparisons.

We assign these two vertebrae to the the family Varanidae due to the following morphological characters: a centrum tapering posteriorly, a precondylar constriction, a ventrally facing cotyle, and a large and flared condyle (Romer 1956, Averianov and Danilov 1997). The Khok Sung vertebrae match well the genus *Varanus* because the condyle is much wider than the posterior end of the centrum and none of the articulatory surface is visible in ventral view. They are also similar in morphology to *Varanus* according to an amphicoelous centrum, condyles facing very dorsally (anterodorsal direction), an oval-shaped cotyle, a short neural spine, and an absence of the zygosphenes and zygantra (Lee 2005). *Varanus* sp. is reported from the Middle Pleistocene of Phnom Loang (Beden and Guérin 1973). Two varanid species, Varanus cf. komodoensis (larger) and *Varanus salvator*, are described from the Middle Pleistocene of Trinil H. K. (Hocknull et al. 2009). The Khok Sung specimens are comparable in size to the recent (e.g., *Varanus salvator*: NMW 39446/1) and fossil (e.g., *Varanus* sp.: RMNH DUB 3 and RMNH DUB 5792 recovered in Trinil H. K., Java) specimens. Identifying these vertebrae more precisely to the species-level, more detailed morphological comparisons need to be made in the future.

## Faunal composition of Khok Sung vertebrate assemblage

**Figure 36.**
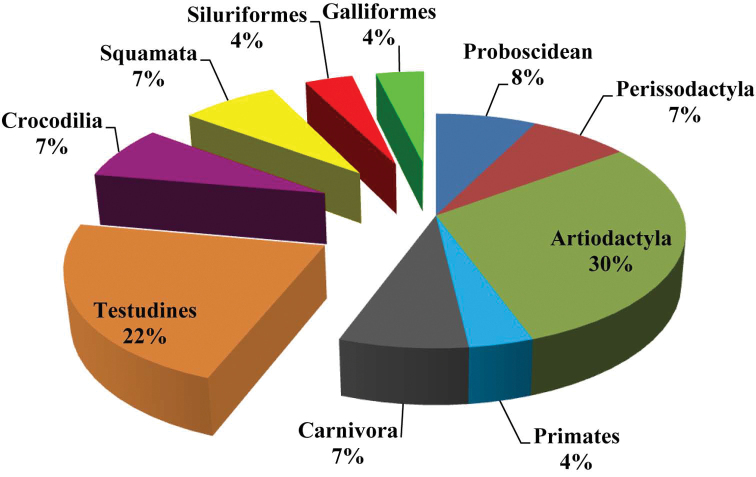
Pie chart showing the species richness of Khok Sung vertebrate fauna.

Nine taxa: seven Testudines, an extinct gharial (Gavialis *bengawanicus*), and a spotted hyaena (*Crocuta crocuta ultima*), have been previously described from Khok Sung by Claude et al. (2011), Martin et al. (2012), and Suraprasit et al. (2015), respectively. In this paper, we studied other undescribed vertebrate fossils from Khok Sung. As a result, fourteen mammalian and three reptilian taxa are identified and added to the faunal list (Tab. 18). Overall, the Khok Sung fauna consists of at least fifteen mammalian (thirteen genera) and ten reptilian (nine genera) species. The mammalian assemblage comprises megaherbivores (> 1000 kg) of approximately 19% of the species (including proboscideans, rhinoceroses, water buffaloes) and other large species of about 37% (including artiodactyls, primates, and carnivores) of the vertebrate fauna (Fig. 36). The most abundant mammal group of the locality is represented by the artiodactyls (9 species). The non-mammalian species consists of about 44% of the total vertebrate fauna. The order Testudines is the most diverse group of non-mammalian taxa in the locality (22% of the fauna). In addition, other vertebrates such as birds and fish are tentatively observed. A single fragmentary cervical vertebra of the bird order Galliformes is also present (Fig. 35L, M). Numerous fish remains including vertebrae (e.g., the specimen DMR-KS-05-03-22-76: Fig. 35N) and pectoral spines (e.g., the specimen DMR-KS-05-04-11-20: Fig. 35P and DMR-KS-05-04-05-25: Fig. 35Q) are assigned to large silurids. Regarding our observations on the Khok Sung vertebrate collection, there are some complete reptile (e.g., carapaces of tortoises and soft-shelled turtles) and fish remains that have not been identified yet. The reptile and fish assemblages would probably indicate a higher diversity than those described from this study, if these undescribed specimens are taxonomically studied in the future. However, it is assumed that the identified mammal remains represent herein the whole mammalian fauna because we have already described almost all of vertebrate fossils (especially skulls and teeth) recovered from the Khok Sung sand pit during the excavation. Only few postcranial remains of mammals such as fragmentary or incomplete bones are unidentified according to the limitation of morphological accessibilities.

**Table 18.** Fauna list of Khok Sung vertebrate fauna.

**Mammalia**		
Proboscidea		
	Stegodontidae	
		Stegodon cf. orientalis
	Elephantidae	
		*Elephas* sp.
Perissodactyla		
	Rhinocerotidae	
		*Rhinoceros sondaicus*
		*Rhinoceros unicornis*
Artiodactyla		
	Bovidae	
		*Bos sauveli*
		*Bos gaurus*
		*Bubalus arnee*
		*Capricornis sumatraensis*
	Cervidae	
		*Axis axis*
		*Panolia eldii*
		*Rusa unicolor*
	Suidae	
		*Sus barbatus*
Primates		
	Cercopithecidae	
		*Macaca* sp.
Carnivora		
	Hyaenidae	
		*Crocuta crocuta ultima* (identified by Suraprasit et al. 2015)
	Canidae	
		*Cuon* sp.
**Reptilia**		
Testudines (identified by Claude et al. 2011)		
	Geoemydidae	
		Batagur cf. trivittata
		*Heosemys annandalii*
		Heosemys cf. grandis
		*Malayemys* sp.
	Trionychidae	
		*Chitra* sp.
		cf. *Amyda* sp.
Crocodilia		
	Gavialidae	
		Gavialis cf. bengawanicus (identified by Martin et al. 2012)
	Crocodylidae	
		Crocodylus cf. siamensis
Squamata		
	Varanidae	
		*Varanus* sp.
	Boidae	
		*Python* sp.
**Actinopterygii**		
	Siluridae indet.	
**Aves**		
Galliformes indet.		

According to the fact that Khok Sung yields only large mammals (> 8 kg), the absence of medium- and small-sized mammal remains is likely due to taphonomic conditions and/or fossil collecting methods. Similarly to most of the Middle and Late Pleistocene fossil sites in Southeast Asia, the biodiversity of Khok Sung large mammals is likely greater than that of present-day faunas (see Appendices 15–18 for the fossil and present-day fauna lists in South China and Southeast Asia) because the Southeast Asian fossil and present-day faunas mostly yield an average of approximately 13 species per site (Tougard and Montuire 2006) and of less than eleven species per area, respectively (Lekagul and McNeely 1988, Corbet and Hill 1992). It is obvious that the Khok Sung mammalian assemblage is characterized by genera and/or species that are similar to the living population in the same area and surrounding regions. However, some mammalian (*Crocuta crocuta*, *Rhinoceros unicornis*, *Axis axis*, and *Sus barbatus*) and reptilian (Batagur cf. trivittata) species in the Khok Sung fauna are no longer present in the region but occur far away from Thailand or even from Southeast Asia. Moreover, two taxa, Stegodon cf. orientalis and Gavialis cf. bengawanicus were present in the locality but became globally extinct later. The Khok Sung vertebrate fauna totally contains 19 of 27 identified taxa that are currently present in Thailand (Tab. 18 and Appendix 18).

## Individual species distribution patterns

Past records and recent distribution patterns of large mammalian species present in Khok Sung are revealed in this work. Paleontological sites in Southeast Asia as well as South China are examined for the Early, Middle, and Late Pleistocene, compared with the modern distribution patterns. We only focus on mammalian taxa assigned to the species-level, including *Stegodon orientalis* and its co-occuring species, *Rhinoceros sondaicus*, *Rhinoceros unicornis*, *Sus barbatus*, *Axis axis*, *Panolia eldii*, *Rusa unicolor*, *Bos sauveli*, *Bos gaurus*, *Bubalus arnee*, and *Capricornis sumatraensis*.

### Stegodontids and elephantids

**Figure 37.**
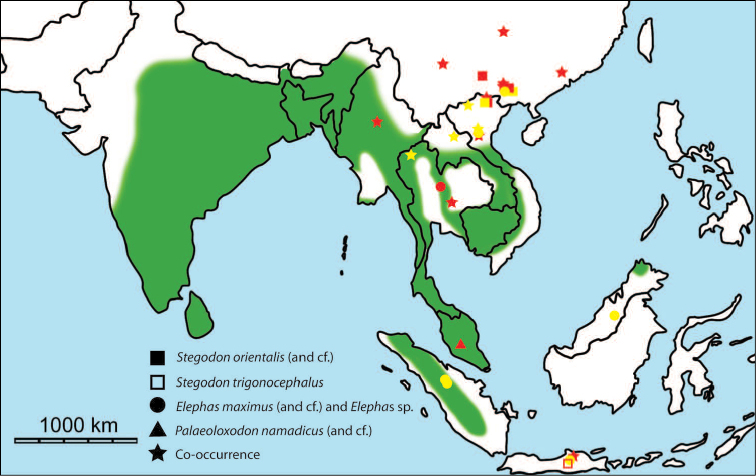
The Middle (red) and Late (yellow) Pleistocene records of stegodontids and relative fossil elephants, and the current distribution (green) of *Elephas maximus* (Indian elephant). Stars indicate the co-occurrence of sympatric proboscideans. The current distribution of Indian elephants is compiled from Lekagul and McNeely (1988).

The earliest records of derived *Stegodon* (e.g., *Stegodon orientalis* from Dayakou (Chen et al. 2013) and *Stegodon trigonocephalus* from Ci Saat (Sondaar 1984, van den Bergh et al. 2001) are likely from the Early Pleistocene. Fossils identified as *Stegodon orientalis* or Stegodon cf. orientalis are recorded from South China (e.g., Daxin (Rink et al. 2008), Hejiang (Zhang et al. 2014), and Panxian Dadong (Han and Xu 1985, Bekken et al. 2004, Schepartz et al. 2005)) and Vietnam (Tham Khuyen, Tham Hai, and Tham Om (Olsen and Ciochon 1990)). Another species, *Stegodon trigonocephalus*, is reported from Javanese localities (van den Bergh et al. 2001). During the Middle to Late Pleistocene, *Stegodon orientalis* co-occurred with *Elephas* sp. or *Elephas maximus* in many localities throughout the Indochinese province (Fig. 37). The two species are found together from the late Middle Pleistocene of Khok Sung and the Late Pleistocene of the Cave of the Monk (Zeitoun et al. 2005, 2010) in Thailand, the early Late Pleistocene of Nam Lot and Tam Hang South (Bacon et al. 2008a, 2011, 2012, 2015) in Vietnam, and the Middle Pleistocene of Ganxian and Wuyun in South China (Chen et al. 2002, Rink et al. 2008, Wang et al. 2007, 2014). *Stegodon orientalis* is found in the Late Pleistocene of Luna (South China) and Keo Leng (northern Vietnam) caves (Olsen and Ciochon 1990, Wang et al. 2014). Perhaps, this species survived until the Holocene in South China (Ma and Tang 1992, Tong and Patou-Mathis 2003, Tong and Liu 2004). The number of species of *Stegodon* lessens from the Early to Late Pleistocene, based on the fossil records of South Chinese localities (Louys et al. 2007). Although *Stegodon orientalis* is likely to have had a less widespread distribution in the Late Pleistocene than in the Middle Pleistocene (Fig. 37), the Pleistocene geographical distribution of this species is only based on a limited number of localities.

A fossil species of *Palaeoloxodon* is reported from several Middle Pleistocene localities in mainland Southeast Asia (Fig. 37), often co-occurring with *Stegodon orientalis* (e.g., the sites of Maba (Han and Xu 1985, Wu et al. 2011) and Tham Khuyen (Olsen and Ciochon 1990)). *Palaeoloxodon* is found in the Late Pleistocene fissure-filling deposits of Hum Hang, Lang Trang, and Ma U’Oi in northern Vietnam (Olsen and Ciochon 1990, Long et al. 1996, Bacon et al. 2004, 2006), similar distribution to that of *Elephas*, but became extinct before the Holocene (Tong and Patou-Mathis 2003, Louys et al. 2007). The cause of global and local extinction of *Stegodon orientalis* and *Palaeoloxodon* is unknown at this time.

*Elephas maximus* is known from the late Middle Pleistocene of Thum Wiman Nakin (northeastern Thailand) (Tougard 1998, 2001), and possibly reached the Indonesian islands of Sumatra, Borneo and Java during the late Pleistocene. *Elephas* is one of two living genera of elephants. The Indian elephant, *Elephas maximus*, is the only extant species. It is distributed throughout mainland Asia (including India, Nepal, Bangladesh, Bhutan, Myanmar, Thailand, Malaysia, Sumatra, Laos, Cambodia, and Vietnam) (Lekagul and McNeely 1988). The Indian elephant is not widespread throughout Southeast Asia as it is not found in central and northeastern Thailand and central southern Myanmar (Fig. 37). Those areas are mostly lowland or highland floodplains today, while Indian elephants prefer deep forest canopy (Lekagul and McNeely 1988, Corbet and Hill 1992). However, this preference for deep forests may be the result of humans encroaching and impacting their preferred habitats (Pushkina et al. 2010). It is possible that *Elephas maximus* became extinct locally in Java before 37 ka as it is absent from the locality of Wajak (dated to 37 ka, van den Brink (1982)). This local extinction is probably due to the drier and cooler climate beginning at 81 ka in Java (van der Kaars and Dam 1995) and/or the loss of rainforest habitats (Storm et al. 2005).

### Javan and Indian rhinoceroses

**Figure 38.**
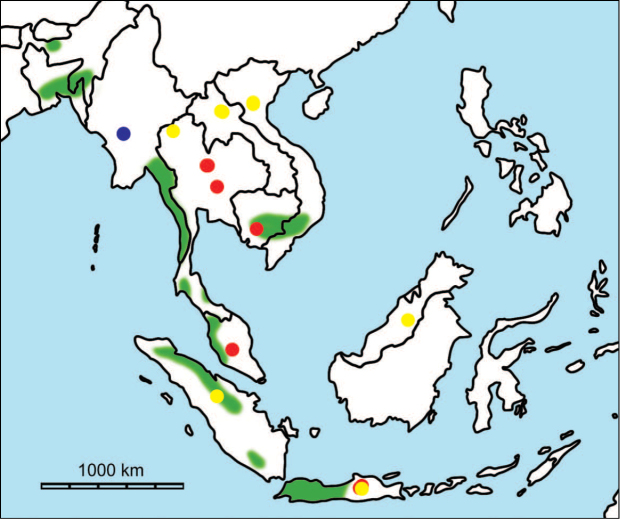
The Early (blue circle), Middle (red circle), and Late (yellow circle) Pleistocene records and the current distribution (green) of *Rhinoceros sondaicus* (Javan rhinoceros). The current distribution of the species is compiled from Groves (1967), Rookmaker (1980), and Groves and Leslie (2011).

The Early Pleistocene records of Asian rhinoceroses are poorly documented in Southeast Asia. Only *Rhinoceros sondaicus* is reported from the upper part of the Irrawaddy Formation, near Pauk Township in central Myanmar (Zin-Maung-Maung-Thein et al. 2006) and from Sangiran in Java (Hooijer 1964) (Fig. 38).

**Figure 39.**
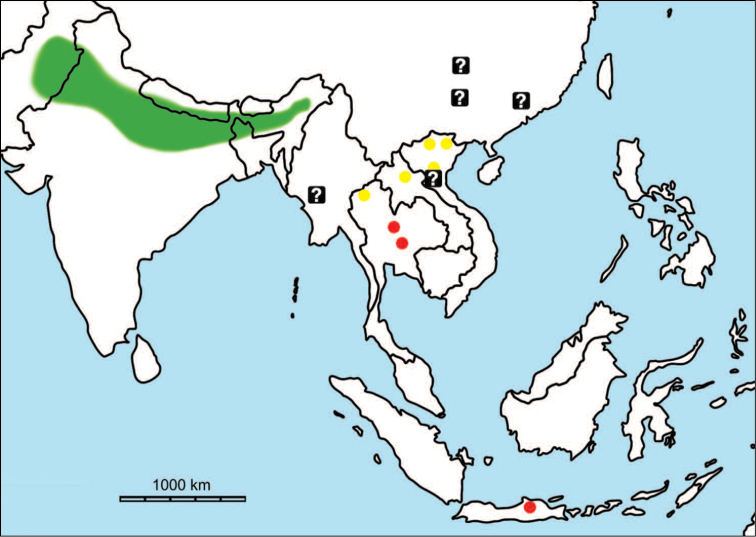
The Middle (red circle) and Late (yellow circle) Pleistocene records and the current distribution (green) of *Rhinoceros unicornis* (Indian rhinoceros). “?” indicates the possible record of *Rhinoceros unicornis* according to Antoine (2012). The current distribution of the species is modified from Laurie et al. (1983).

The Middle Pleistocene record, especially the late Middle Pleistocene, includes numerous reports of Asian rhinoceroses (Figs 38 and 39). In the Indochinese subregion during the Middle Pleistocene, fossils of *Rhinoceros unicornis* are found from Hsingan (Kahlke 1961) and Maba (Wu et al. 2011) in South China, from Yenangyaung in Myanmar (sensu Antoine 2012), from Tham Hai and Tham Om in northern Vietnam (sensu Antoine 2012). During the late Middle Pleistocene, fossils of *Rhinoceros unicornis* are known from Thum Prakai Phet (Tougard 1998) in northeastern Thailand. Remains of *Rhinoceros sondaicus* are recovered from the Middle Pleistocene of Phnom Loang (Beden and Guérin 1973). The only co-occurrences of these two species are from the late Middle Pleistocene of Thum Wiman Nakin (Tougard 1998, 2001) and from our discoveries at Khok Sung. In the Sundaic subregion, fossils of Indian rhinoceroses have been described from the Middle Pleistocene of Tumbun (Malaysia) and Trinil H. K. (Java) (Hooijer 1962, Medway 1972, van den Bergh et al. 2001) and from the early Middle Pleistocene of Kedung Brubus where Javan rhinoceroses co-occurred (Hooijer 1946). In other biogeographic regions, *Rhinoceros unicornis* occurred in Yenchingkou (central eastern China) (sensu Antoine 2012). According to original faunal descriptions, many Middle Pleistocene localities in China and Vietnam yielded fossil specimens of *Rhinoceros sinensis*. This species was later synonymized with *Rhinoceros unicornis* by Antoine (2012). However, *Rhinoceros sinensis* is recently recognized as a valid species (Yan et al. 2014), so there remains some confusion about the presence of *Rhinoceros unicornis* in many localities.

During the late Pleistocene, Javan and Indian rhinoceroses were widespread in Indochinese subregion (Figs 38 and 39). They co-occurred in the Cave of the Monk (Ban Fa Suai, northern Thailand) (Zeitoun et al. 2005, 2010), in Nam Lot and Tam Hang South (northern Laos) (Bacon et al. 2008a, 2011, 2012, 2015), and in Duoi U’Oi and Ma U’Oi (northern Vietnam) (Bacon et al. 2004, 2006, 2008b). Indian rhinoceros fossils were also found in the caves of Ham Hang and Keo Leng, northern Vietnam (Olsen and Ciochon 1990), while Javan rhinoceroses were recovered from Niah caves (Borneo, Malaysia) (Medway 1972, Harrison 1996) and several Indonesian localities: Lida Ajer and Sibrambang in Sumatra (de Vos 1983) and Punung, Gunung Dawung, and Wajak in Java (Badoux 1959, van den Brink 1982, Storm et al. 2005, 2013). Indian rhinoceroses seem to go extinct in Java after the middle Middle Pleistocene, as none are reported from Trinil H. K. (dated to ~540-430 ka, Joordens et al. (2014)) and early Late Pleistocene to Holocene sites.

Nowadays, the Indian rhinoceros is locally extinct from the Thai territory and several other countries in Southeast Asia. The species is restricted to Nepal and India and some parts of northernmost Myanmar (Laurie et al. 1983) (Fig. 39). The Javan rhinoceros survives across the Indochinese Peninsula and the Sundaic subregions (Groves and Leslie 2011) but became extinct in the island of Borneo during the Holocene (Medway 1960, Cranbrook 2000, Cranbrook et al. 2000, Cranbrook and Piper 2007) (Fig. 38). The modern co-occurrences of the two species are restricted to a small area in eastern India (Antoine 2012). In the Holocene, the Javan rhinoceros likely co-occurred with the Sumatran rhinoceros, *Dicerorhinus sumatrensis*, but they are not sympatric today almost certainly because of human induced habitat loss leading to reduction of their geographic range during the last century (Groves and Leslie 2011).

### Bearded pigs

**Figure 40.**
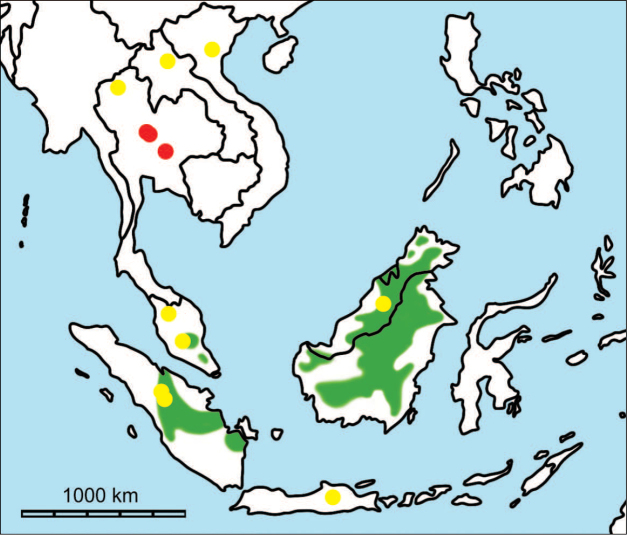
The Middle Pleistocene (red circle) and Late Pleistocene to Holocene (yellow circle) records and the current distribution (green) of *Sus barbatus* (bearded pig). The current distribution of the species is compiled from Corbet and Hill (1992).

During the Middle Pleistocene, *Sus barbatus* (bearded pig) is known from the caves of Thum Wiman Nakin and Thum Prakai Phet (Tougard 1998, 2001) and the terrace deposit of Khok Sung (Fig. 40). Among these Thai localities, *Sus barbatus* co-occurred with *Sus scrofa* at least in Thum Wiman Nakin and Thum Prakai Phet.

In the late Pleistocene, *Sus barbatus* is well-documented from many localities, extending its geographic distribution across Sumatra, Borneo, and Java. This species is likely more widespread in the late Pleistocene than the Middle Pleistocene (Fig. 40). In Indochinese and Sundaic subregions, the co-occurrence of *Sus barbatus* and *Sus scrofa* is known from the “Cave of the Monk” (Ban Fa Suai) in northern Thailand (Zeitoun et al. 2005, 2010), Tam Hang South in northern Laos (Bacon et al. 2008, 2011, 2015), Batu caves and Gua Cha (Holocene) in Peninsular Malaysia (Groves 1985, Ibrahim et al. 2013), Lida Ajer and Sibrambang in Sumatra (de Vos 1983), and Punung in Java (Badoux 1959). Only fossils of bearded pigs are collected from the latest Pleistocene of Niah Cave, Borneo (Medway 1972, Harrison 1996).

Today *Sus barbatus* is restricted to Peninsular Malaysia, Sumatra, and Borneo (Corbet and Hill 1992) (Fig. 40), in contrast with its widespread distribution across the Indochinese subregion during the Middle to Late Pleistocene. This species dispersed to Indonesian islands by the Late Pleistocene, as it is recorded from Punung of Java (Badoux 1959). After the land bridges submerged by rising sea level, some populations of *Sus barbatus* were probably trapped on islands (Tougard 2001). Later on, *Sus barbatus* went extinct in mainland Southeast Asia after the late Pleistocene. The cause of local extinction of *Sus barbatus* in mainland Southeast Asia is unknown at this time. This taxon also became locally extinct later in Java as none is recorded from the Late Pleistocene of Wajak site (van den Brink 1982). The drier and cooler climates during the middle Middle Pleistocene or the reduction of rainforest habitats possibly explain the local extinction for bearded pigs in Java.

### Chitals

**Figure 41.**
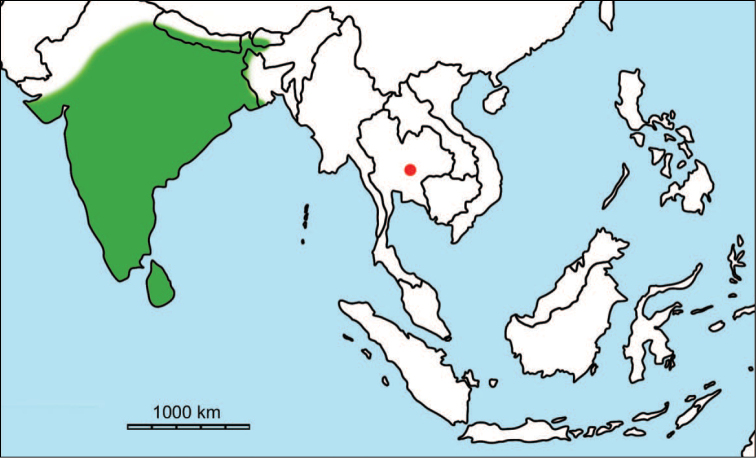
The Middle Pleistocene record (red circle) and the current distribution (green) of *Axis axis* (chital). The current distribution of the species is compiled from Duckworth et al. (2008a).

Fossils of *Axis axis* (chital) have never been previously recorded from Thailand but were present in mainland Southeast Asia, at least in Khok Sung, during the late Middle Pleistocene (Fig. 41). Only Axis cf. porcinus is reported from the Late Pleistocene of the Cave of the Monk (Zeitoun et al. 2005, 2010). Other species of *Axis* are also described in Asia. *Axis shansius* and *Axis rugosa* are reported from the Early Pleistocene of China (Han and Xu 1985), whereas *Axis lydekkeri* is recorded from the Early to Middle Pleistocene of Java (Gruwier et al. 2015). The Bawean deer, *Axis kuhli*, is also reported in Java since the Holocene (van den Bergh et al. 2001, Moigne et al. 2004).

Nowadays *Axis axis* is restricted to the Indian Subcontinent (India, Nepal, Sikkim, and Sri Lanka) (Fig. 41). Its habitat preferences are grasslands and open forests (Nowak 1999). The Pleistocene chital has a different geographical distribution as it was present in Khok Sung. The distribution range of *Axis axis* in the Pleistocene is probably wider than in the present day. Rainforests became more dominant across Southeast Asia during the Late Pleistocene (Heaney 1991, Meijaard 2003, Louys et al. 2007). The local extinction of the chital in Thailand is likely caused by the reduction of open grasslands. In the future, additional fossil records of *Axis axis* in Southeast Asia would allow to address some issues related to its local extinction, as well as its past distribution.

### Eld’s and sambar deer

**Figure 42.**
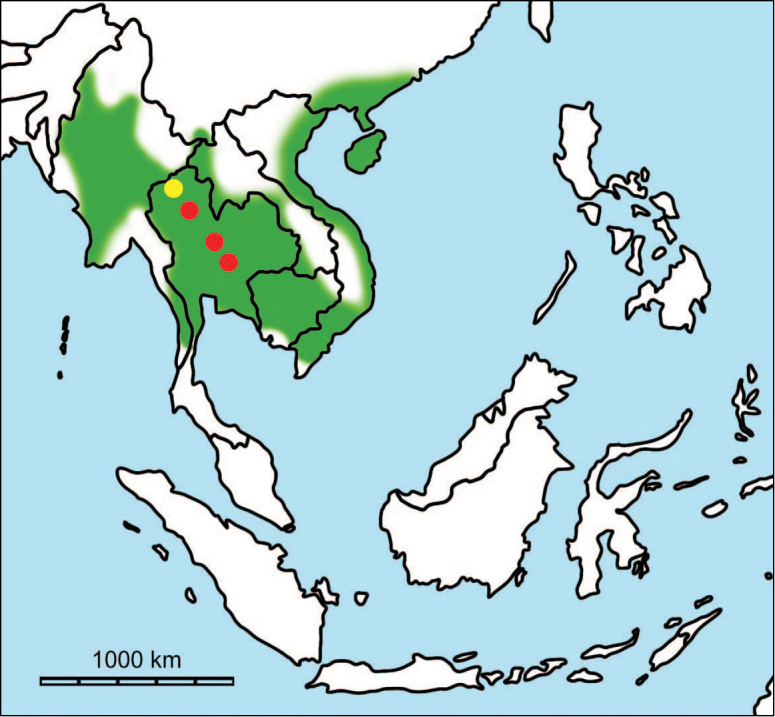
The Middle (red circle) and Late (yellow circle) Pleistocene records and the current distribution (green) of *Panolia eldii* (Eld’s deer). The current distribution of the species is compiled from Lekagul and McNeely (1988).

**Figure 43.**
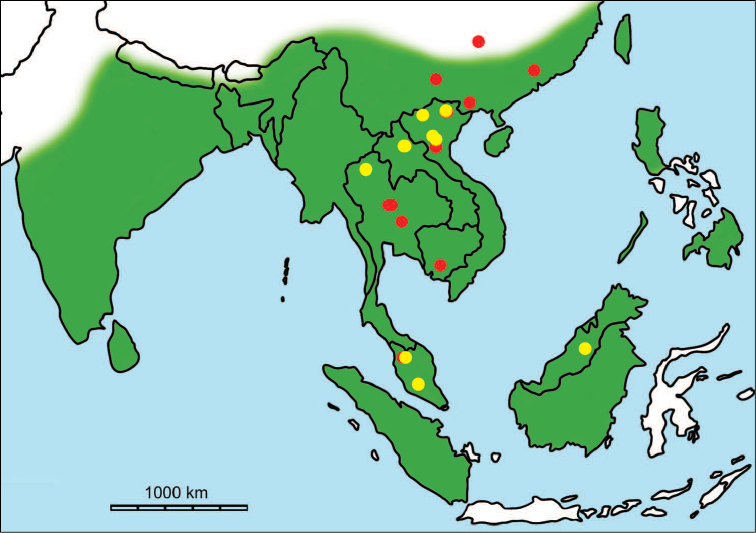
The Middle Pleistocene (red circle) and Late Pleistocene to Holocene (yellow circle) records and the current distribution (green) of *Rusa unicolor* (sambar deer). The current distribution of the species is compiled from Lekagul and McNeely (1988).

The Eld’s deer is known from the Middle Pleistocene of Thailand. Fossils of *Panolia eldii* are collected from the caves of Thum Wiman Nakin and Kao Pah Nam (Pope et al. 1981, Tougard 1988, 2001) and from the Khok Sung sand pit (Fig. 42). Fossils of sambar deer are widely recorded from many Middle Pleistocene sites in mainland Southeast Asia: Hejiang, Panxian Dadong, and Maba in South China (Han and Xu 1985, Bekken et al. 2004, Schepartz et al. 2005, Wu et al. 2011, Zhang et al. 2014), Thum Wiman Nakin (Tougard 1998, 2001), Thum Prakai Phet (Tougard 1998, Filoux et al. 2015), and Khok Sung in Thailand, Tham Khuyen, Tham Hai, and Tham Om in Vietnam (Olsen and Ciochon 1990), Phnom Loang and Boh Dambang in Cambodia (Beden and Guérin 1973, Demeter et al. 2013), and Badak Cave in Peninsular Malaysia (Ibrahim et al. 2013) (Fig. 43). Both taxa co-occurred in Thum Wiman Nakin and Khok Sung.

During the Late Pleistocene, the Eld’s and sambar deer co-occurred in the Cave of the Monk (Ban Fa Suai), northern Thailand (Zeitoun et al. 2005, 2010). The sambar deer is widespread across Laos (Nam Lot and Tam Hang South (Bacon et al. 2008a, 2011, 2012, 2015)), Vietnam (Hang Hum, Keo Leng, Lang Trang, Duoi U’Oi, and Ma U’Oi (Olsen and Ciochon 1990, Long et al. 1996, Bacon et al. 2004, 2006, 2008b)), Peninsular Malaysia (Batu Cave, Gua Gunung Runtuh, and Gua Cha (Holocene) (Groves 1985, Davidson 1994, Ibrahim et al. 2013)), and Borneo (Niah Cave (Medway 1972, Harrison 1996, Barker et al. 2007)). However, none are recorded in Sumatra and Java (Fig. 43).

Nowadays, *Panolia eldii* is restricted to the Indochinese province (Fig. 42). *Rusa unicolor* is a widespread species native to the Indian Subcontinent, southern China, and Southeast Asia (both Indochinese and Sundaic subregions with the exception of Java (Fig. 43)) (Lekagul and McNeely 1988).

### Koupreys, gaurs, and wild water buffaloes

**Figure 44.**
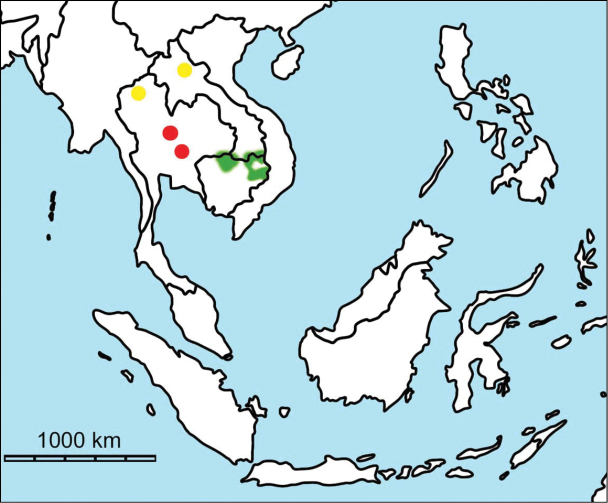
The Middle (red circle) and Late (yellow circle) Pleistocene records and the current distribution (green) of *Bos sauveli* (kouprey). The current distribution of the species is compiled from Lekagul and McNeely (1988) and Timmins et al. (2008).

**Figure 45.**
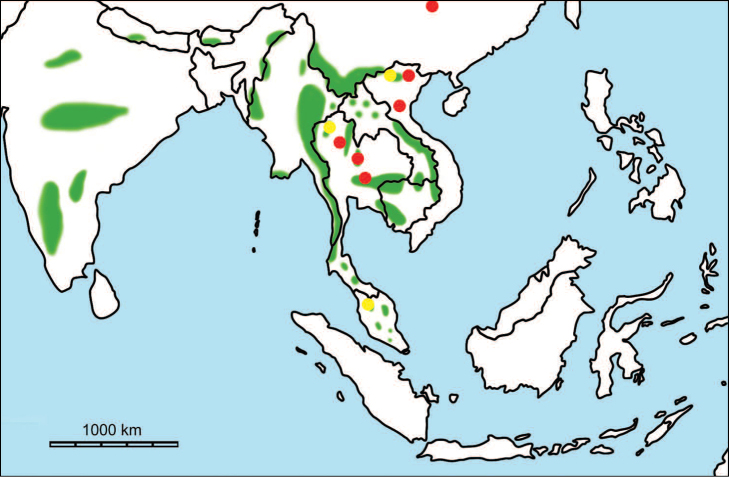
The Middle (red circle) and Late (yellow circle) Pleistocene records and the current distribution (green) of *Bos gaurus* (gaur). The current distribution of the species is compiled from Lekagul and McNeely (1988) and Duckworth et al. (2008b).

**Figure 46.**
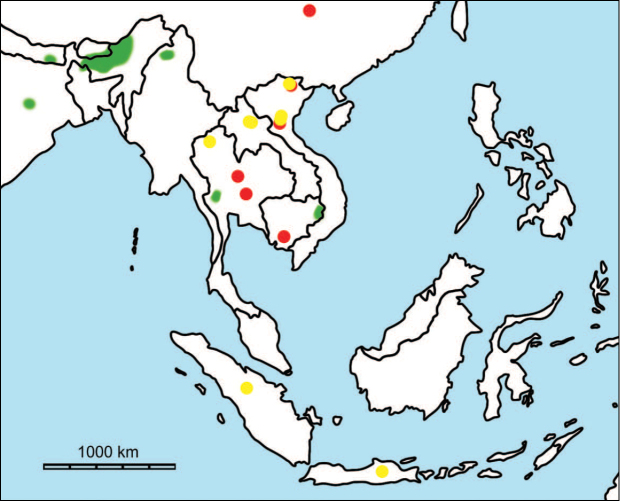
The Middle (red circle) and Late (yellow circle) Pleistocene records and the current distribution (green) of *Bubalus arnee* (wild water buffalo). The current distribution of the species is compiled from Lekagul and McNeely (1988) and Hedges et al. (2008).

Large bovids in Southeast Asia currently comprise four wild species: *Bos sauveli* (kouprey), *Bos javanicus* (banteng), *Bos gaurus* (gaur), and *Bubalus arnee* (wild water buffalo). Bantengs, gaurs, and koupreys presumably shared a common ancestor at 2.6 Ma (Plio-Pleistocene) and their lineages split in a short period of time (i.e., between 200 and 300 ka) based on the molecular estimations of divergence times (Hassanin and Ropiquet 2004). These molecular estimations are congruent with the fossil records of bantengs and gaurs in Asia. Fossil remains attributed to these species have been recorded in Southeast Asia since the Middle Pleistocene. The co-occurrence of these Pleistocene large bovids is reported from Thum Wiman Nakin (Tougard 1998, 2001) and Khok Sung in northeastern Thailand (Figs 44–46). Fossil remains of gaurs are also reported from the Middle Pleistocene of Kao Pah Nam in northern Thailand (Pope et al. 1981), the middle Middle Pleistocene of Tham Khuyen and the late Middle Pleistocene of Tham Om in Vietnam (Olsen and Ciochon 1990), and the Middle Pleistocene of Yenchingkou in central eastern China (Colbert and Hooijer 1953) (Fig. 45). In addition, remains of fossil water buffaloes are described from the late Middle Pleistocene of Phnom Loang and Boh Dambang in Cambodia (Beden and Guérin 1973, Demeter et al. 2013).

During the Late Pleistocene, the locality of the Cave of the Monk (Ban Fa Suai) yielded remains of these bovid species (cf.) (Zeitoun et al. 2005, 2010). Other localities yielded either only one species of *Bos* or the co-occurrence of two *Bos* species and *Bubalus*. *Bubalus arnee* occurred not only in Sumatra but also in Java during the latest Middle/early Late Pleistocene according to their fossil records in Sibrambang and Punung (Badoux, 1959, de Vos 1983, Storm and de Vos 2006), respectively (Fig. 46). Both taxa disappeared subsequently in Sumatra either after the early Late Pleistocene or during the Holocene. Neither koupreys nor gaurs are identified in insular Southeast Asia, thus most likely restricted to mainland Southeast Asia (Fig. 44).

The historical distribution of koupreys during the last century is restricted to Cambodia, southern Laos, southeastern Thailand, and western Vietnam (Lekagul and McNeely 1988, Corbet and Hill 1992). They become globally extinct today. Gaurs recently occur throughout mainland South and Southeast Asia and Sri Lanka (Lekagul and McNeely 1988, Duckworth et al. 2008b) (Fig. 45). Nowadays, they are also present in South China where their fossils have never been found. Wild water buffaloes are currently native to Bhutan, Cambodia, India, Myanmar, Nepal, and Thailand (Lekagul and McNeely 1988, Hedges et al. 2008). They become locally extinct in Vietnam (likely), Laos, Indonesia, Sri Lanka, and Bangladesh (Fig. 46).

Overall, the Pleistocene large bovid species in Southeast Asia is more widespread than the modern population. The anthropogenic impacts on the environments and landscapes seem to have caused the reduction of large bovid population in several areas during the past decade. The koupreys is more widely distributed during the Pleistocene than today (Fig. 44). In addition to the human activity, the cause of reduction and extinction of koupreys is likely due to their high degrees of habitat specificity such as deciduous dipterocarp forests and especially in areas with extensive grasslands (Timmins et al. 2008), and/or according to high levels of niche competition with other large bovids.

### Sumatran serows

**Figure 47.**
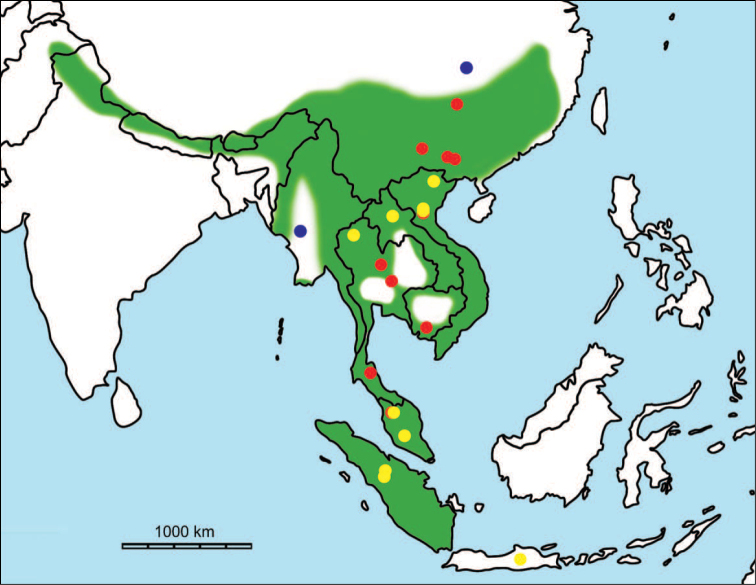
The Early Pleistocene (blue circle), Middle Pleistocene (red circle), and Late Pleistocene (yellow circle) records and the current distribution (green) of *Capricornis sumatraensis* (serow). The current distribution of the species is compiled from Lekagul and McNeely (1988).

The possible earliest records of *Capricornis sumatraensis* are from the middle Early Pleistocene site of Gongwangling (Hu and Qi 1978, Han and Xu 1985), dated to 1.63 Ma (Zhu et al. 2015), in central mainland China and from the Early Pleistocene of the Upper Irrawaddy Formation (Colbert 1938, Takai et al. 2006) in central Myanmar. *Capricornis sumatraensis* during the Middle Pleistocene is widespread throughout mainland Asia and Southeast Asia (Fig. 47). It is known from the Middle Pleistocene of Yenchingkou in central eastern China (Colbert and Hooijer 1953), Wuming, Panxian Dadong, and Wuyun in South China (Han and Xu 1985, Chen et al. 2002, Bekken et al. 2004, Schepartz et al. 2005, Rink et al. 2008, Wang et al. 2007, 2014), Tham Om in Vietnam (Olsen and Ciochon 1990), Thum Wiman Nakin, Thum Prakai Phet, and Khok Sung in Thailand (Tougard 1998, 2001, Filoux et al. 2015), Boh Dambang in Cambodia (Demeter et al. 2013), and Badak Cave in Peninsular Malaysia (Ibrahim et al. 2013). Fossils of *Capricornis sumatraensis* are also described from the latest Middle/early Late Pleistocene of Lida Ajer and Sibrambang in Sumatra and of Punung in Java (Badoux 1959, de Vos 1983, van den Bergh et al. 2001, Storm and de Vos 2006). However, no serows are recorded from Borneo.

The Sumatran serow is a widespread species, native to mountain forests on the Himalayan range (northern India, Sikkim, and Nepal) of the Indochinese subregion (Southern China, Myanmar, Thailand, Laos, Cambodia, Vietnam, and Peninsular Malaysia) and on the island of Sumatra (Lekagul and McNeely 1988) (Fig. 47). *Capricornis sumatraensis* became locally extinct in Java during the middle Late Pleistocene according to the lack of fossil records in Wajak (~37 ka). The advocated cause for the local extinction of serows is possibly related to the unfavorable climatic conditions. The drier and cooler climate that occurred after 81 ka in Java (van der Kaars and Dam 1995) probably affects significantly the niche preferences of forest-dwelling taxa.

## Faunal comparisons of the assemblage with other penecontemporaneous assemblages

For the comparisons of vertebrate faunas between Khok Sung and other Pleistocene sites, we focus only on large mammals (for the mammalian fauna lists of the Middle to Late Pleistocene of Southeast Asian sites, see Appendices 16 and 17). The identification of the family level referred to “indet.” and the species level designated “sp.” are herein excluded from our comparisons. The Khok Sung large mammalian assemblage yields most extant and some extinct taxa, which are characteristic of the *Ailuropoda*-*Stegodon* assemblage. Compared to other Thai Pleistocene faunas, the Khok Sung mammalian assemblage shares 10 ten species with Thum Wiman Nakin (Tougard 1998, 2001), six species with Thum Prakai Phet (Tougard 1998, Filoux et al. 2015), and nine species with the Cave of the Monk (Zeitoun et al. 2005, 2010). However, most of the mammalian taxa from the Cave of the Monk are assigned to “cf.” (the open nomenclature) and the presence of fossil spotted hyaena, *Crocuta crocuta*, in this locality is still doubtful, i.e. only one fragmentary tooth is identified as belonging to Hyaenidae indet. by Zeitoun et al. (2005, 2010) (Appendix 16). Compared to the surrounding Pleistocene faunas, the Khok Sung mammalian assemblage has taxonomic similarities of seven species with Nam Lot (Bacon et al. 2012, 2015), eight species with Tam Hang South (Bacon et al. 2008a, 2011, 2015), four species with Tham Khuyen (Olsen and Ciochon 1990), two species with Tham Hai (Olsen and Ciochon 1990), five species with Tham Om (Olsen and Ciochon 1990), four species with Hang Ham (Olsen and Ciochon 1990), five species with Keo Leng (Olsen and Ciochon 1990), four species with Lang Trang (Long et al. 1996), three species with Ma U’Oi (Bacon et al. 2004, 2006), six species with Duoi U’Oi (Bacon et al. 2008b), four species with Boh Dambang (Demeter et al. 2013), and four species with Phnom Loang (Beden and Guérin 1973) (Appendices 16 and 17). The Khok Sung assemblage is more different from other Pleistocene faunas, especially from the Indonesian islands, which mainly yield endemic forms. According to the number of shared taxa, the Khok Sung mammalian assemblage more nearly resembles diversified faunas from Thum Wiman Nakin, Thum Phra Khai Phet, Nam Lot, and Tam Hang South than the others.

The Khok Sung assemblage shares at least one similar archaic mammal taxon such as *Crocuta crocuta ultima* and *Stegodon orientalis*, with these faunas. *Crocuta crocuta* is also recorded from Thum Wiman Nakin, Thum Prakai Phet, and Nam Lot, whereas *Stegodon orientalis* is reported from two Laotian sites: Nam Lot and Tam Hang South. By the way, most of forest dwelling and carnivorous taxa that are representatives of Middle Pleistocene mammalian assemblages such as *Ailuropoda melanoleuca* (giant panda), *Ursus thibetanus* (Asiatic black bear), *Pongo pygmaeus* (orang-utan), *Muntiacus muntjak* (Southern red muntjac), and *Tapirus indicus* (Malayan tapir) are absent in Khok Sung. The paleoenvironments of Khok Sung corresponded to a floodplain near the river channel (Duangkrayom et al. 2014, Suraprasit et al. 2015). The absence of most of these taxa in Khok Sung is likely explained by the local environments that are unfavourable to those species. Although some forest-inhabiting taxa (e.g., *Elephas maximus* and *Capricornis sumatraensis*) are found in the locality, these fossils (rare, fragmentary, or represented by isolated teeth only) were transported from the surrounding upland forests by the river.

The degree of the faunal similarity also depends on the number of identified taxa for each site. We further analyse the relationships between the geographic regions and faunas in Southeast Asia, using the Simpson coefficient of faunal similarity (Tab. 19) performed with the multivariate clustering analysis. The final dataset analysed for the similarity comprises 18 localities and 85 taxa. The analysis is based on the presence/absence of mammalian taxa in the fauna lists complied from literatures (Appendices 15 and 16).

**Table 19.** Similarity matrix based on the Simpson coefficients. Locality abbreviations: YCK, Yenchingkou; KLS, Koloshan; DX, Daxin; HJ, Hejiang; GX, Ganxian; PXDD, Panxian Dadong; WY, Wuyun; MB, Maba; HST, Hoshantung; KS, Khok Sung; TWN ; Thum Wiman Nakin; TPKP, Thum Phra Khai Phet; TK, Tham Khuyen; TO, Tham Om; BDB, Boh Dambang; KDBB, Kedung Brubus; TNHK, Trinil Hauptknochenschicht; ND, Ngandong.

	YCK	KLS	DX	HJ	GX	PXDD	WY	MB	HST	KS	TWN	TPKP	TK	TO	BDB	KDBB	TNHK	ND
**YCK**	1.00																	
**KLS**	0.38	1.00																
**DX**	0.54	0.31	1.00															
**HJ**	0.55	0.27	0.45	1.00														
**GX**	0.50	0.20	0.40	0.40	1.00													
**PXDD**	0.53	0.46	0.46	0.55	0.30	1.00												
**WY**	0.53	0.23	0.38	0.45	0.70	0.33	1.00											
**MB**	0.94	0.38	0.62	0.55	0.50	0.44	0.47	1.00										
**HST**	0.50	0.30	0.20	0.30	0.20	0.40	0.40	0.50	1.00									
**KS**	0.50	0.00	0.08	0.18	0.10	0.25	0.17	0.25	0.10	1.00								
**TWN**	0.48	0.15	0.31	0.27	0.60	0.24	0.40	0.50	0.30	0.83	1.00							
**TPKP**	0.58	0.17	0.17	0.36	0.30	0.33	0.33	0.50	0.30	0.50	0.92	1.00						
**TK**	0.63	0.31	0.54	0.55	0.60	0.41	0.40	0.63	0.40	0.33	0.53	0.42	1.00					
**TO**	0.94	0.38	0.54	0.55	0.50	0.50	0.47	0.81	0.40	0.42	0.63	0.50	0.75	1.00				
**BDB**	0.80	0.10	0.20	0.20	0.30	0.40	0.50	0.60	0.20	0.40	0.80	0.60	0.60	0.60	1.00			
**KDBB**	0.11	0.08	0.00	0.09	0.00	0.06	0.07	0.13	0.10	0.17	0.22	0.17	0.06	0.13	0.10	1.00		
**TNHK**	0.21	0.08	0.08	0.09	0.00	0.14	0.14	0.21	0.10	0.08	0.14	0.17	0.07	0.14	0.30	0.64	1.00	
**ND**	0.10	0.10	0.00	0.10	0.00	0.10	0.10	0.10	0.10	0.00	0.10	0.00	0.00	0.10	0.00	0.90	0.60	1.00

**Figure 48.**
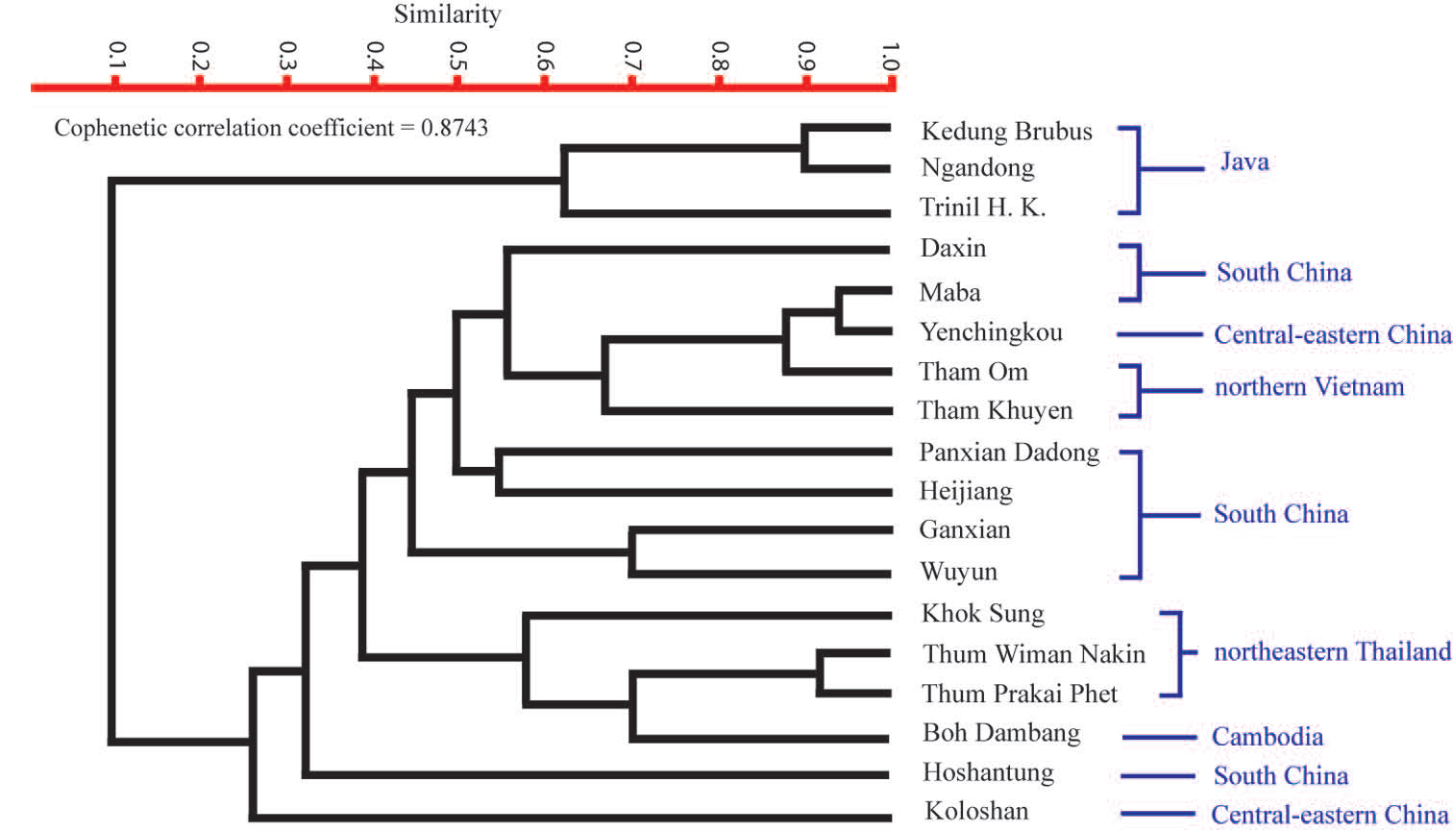
Cluster analysis of the Middle Pleistocene mammalian fossil records in Southeast Asian and some central-eastern Chinese localities based on the Simpson coefficients.

As a result, the Middle Pleistocene Southeast Asian taxa reveal two distinct associations (Javanese and mainland Southeast Asian faunas) (Fig. 48). Within the mainland Southeast Asian assemblages, the cluster analysis resolves two different groups between the Thai, Combodian, Vietnamese, and Chinese faunas (South China and Yenchingkou) and the central-eastern Chinese one (Koloshan) (Fig. 48). Among South Chinese localities, Hoshantung fauna is a distinct subcluster separated from other mainland Southeast Asian faunas. Hoshantung probably represents a different biochronological age from each other rather than high levels of endemism. The Thai and Cambodian faunas constitute a distinctive subgroup that is differentiated from the Vietnamese and Chinese assemblages. Within the Thai and Cambodian members, the Khok Sung fauna characterizes a distinct subcluster separated from three late Middle Pleistocene assemblages: Thum Wiman Nakin, Thum Prakai Phet, and Boh Dambang (Fig. 48), although the fauna of Khok Sung is most similar in composition to that of Thum Wiman Nakin according to the Simpson’s index (Tab. 19). This is likely due to the convention of the UPGMA method, which produces equal length branches from all nodes, and to the effects of higher faunal similarity between Thum Wiman Nakin and two other faunas.

Overall, this analysis suggests initially that the differences in species composition and distribution do not follow a trend of the latitudinal gradient north to south, but show spatial and time variability of large mammalian fauna in Southeast Asia. The main problems of mammalian fauna comparisons in Southeast Asia are likely due to the poorly-known species diversity and/or the imprecisely chronological determination in several localities.

## Discussion

### Biochronology of Khok Sung fauna

According to the similarity analysis of the fauna, the mammalian fauna composition of Khok Sung is considerably different from the Early to early Middle Pleistocene assemblage of Java. This suggests an inconsistent age of the Early Pleistocene for Khok Sung. The Khok Sung assemblage is highly comparable in composition to three late Middle Pleistocene faunas: Thum Wiman Nakin (> 169 ka, Esposito et al. (1998, 2002)), Thum Prakai Phet (Tougard 1998, Filoux et al. 2015), and Boh Dambang (Demeter et al. 2013). However, our faunal comparisons suggest that the biochronological age of Khok Sung is possibly different, slightly older or younger, from those three localities according to some of the compositional dissimilarity. Two early Late Pleistocene sites: Nam Lot (≈86-72 ka, Bacon et al. (2015)), and Tam Hang South (≈94-60 ka, Bacon et al. (2015)) possibly remains contemporaneous according to the occurrence of several taxa sharing with Khok Sung (> 7 species).

On the basis of the previous paleomagnetic data analysed by Suraprasit et al. (2015), a short reversal polarity registered in a fine layer of silty mud lenses (Fig. 2) that occurs within the Brunhes normal chron could be presumably correlated to the geomagnetic excursions of either “Iceland Basin” (188 ka) or “Pringle Falls” (213 ka). In addition, the short reversal event of the paleomagnetic field in Brunhes normal chron is possibly correlated to “Blake” excursion (dated to around 120 ka, Lund et al. (2001)). However, we suggest a late Middle Pleistocene age rather than a Middle/Late Pleistocene transition according to the occurrence of several archaic taxa and to the closest faunal similarity with Thum Wiman Nakin.

### Evolutionary and biogeographic affinities of Khok Sung fauna

Relationships of the Khok Sung vertebrate fauna for dispersal events from India to Java has been first proposed by Martin et al. (2012). *Gavialis bengawanicus* and *Crocodylus siamensis* as well as monitor lizards and pythons are known as typical taxa associated with the *Stegodon*-*Homo erectus* fauna, which presumably originated from the Miocene-Pliocene of Siwalik faunas in India and Pakistan (Head 2005, de Vos 2007, Hocknull et al. 2009, Delfino and de Vos 2010, Martin et al. 2012). These taxa migrated from mainland Southeast Asia to Java, via the Siva-Malayan route, by the Early Pleistocene as they are first recorded from the Early Pleistocene of Java (von Koenigswald 1935, de Vos 1995, 2007, de Vos and Long 2001, Delfino and de Vos 2010) (Fig. 1 and Appendix 16). According to the occurrence of Gavialis cf. bengawanicus in Khok Sung, Martin et al. (2012) hypothesized that this species reached Java through the fluvial drainages of Sunda shelf (rather than the dispersal by sea) during a low sea level event (with a minimum of about 170 m below the present day) of the Early-Middle Pleistocene transition (around 0.8 Ma) (Prentice and Denton 1988, van den Bergh et al. 2001, van der Geer et al. 2010) (Fig. 50). In the light of this scenario, *Gavialis bengawanicus* might have appeared either earlier than or during the Early Pleistocene in Thailand.

However, in terms of faunal age, this scenario is no longer consistent because the Khok Sung fauna is recently attributed to a late Middle Pleistocene age (Suraprasit et al. 2015), younger than *Gavialis* and *Crocodylus*-bearing localities in Java. We propose that gharials and some other vertebrates (e.g., a freshwater crocodile, a large varanid, and a python) present in Khok Sung are possibly geographical remnants of the former Siva-Malayan fauna that survived until the late Middle Pleistocene as they occurred earlier in Java. Otherwise, these vertebrates possibly appeared either firstly or repeatedly (if the local extinction of those taxa previously occurred) in Thailand during the late Middle Pleistocene. Several cyclic occurrences of high amplitude glacial periods (~50 times since the last 2.7 Ma, Woodruff (2010)), related to the sea level lowering, during the Early to Middle Pleistocene (Prentice and Denton 1988, van der Kaars 1991, Zheng and Lei 1999) could provide high possibilities to facilitate faunal exchange between mainland and insular Southeast Asia (via the land bridges or Sunda shelf). The faunal exchanges by corridor and/or filter bridge dispersal between Thailand and Java might have occurred habitually during the glacial events.

Based on the occurrence of mammalian taxa in Khok Sung, we suggest an alternative relevance of this fauna for the “Sino-malayan” dispersal events from mainland Southeast Asia to Java (Fig. 1). This evidence is supported by the faunal turnover that occurred in Punung (Java), around 128 to 118 ka dated by luminescence and U-series analysis performed on the breccias (Westaway et al. 2007). The modern rainforest assemblage, known as the *Pongo*-*Homo sapiens* or *Elephas*-*Homo sapiens* fauna, has replaced the former *Stegodon*-*Homo erectus* faunal association in Java during since latest Middle Pleistocene (Westaway et al. 2007). The new faunal elements include *Elephas maximus*, *Pongo pygmaeus*, *Symphalangus syndactylus* (siamang), *Macaca nemestrina* (pig-tailed macaque), *Panthera tigris* (tiger), *Dicerorhinus sumatrensis* (Sumatran rhinoceros), *Helarctos malayanus* (sun bear), *Capricornis sumatraensis*, *Bubalus arnee*, *Sus scrofa*, and *Sus barbatus*. The Khok Sung mammalian assemblage consists of at least 4 of forest dwelling mammals: *Capricornis sumatraensis*, *Bubalus arnee*, *Sus barbatus*, and *Elephas* sp. (Appendices 16 and 17). These taxa presumably migrated from mainland Southeast Asian to Java and some of them are living today in the mainlafnd Southeast Asia (van den Bergh et al. 2001, van der Geer et al. 2010). The presence of exclusive tropical rainforest species in Punung indicates that their migration event could have occurred following the dry and open woodland environments of the penultimate glaciations at about 135 ka (de Vos 1983, de Vos et al. 1994). These mammals migrated southward to the exposed Sunda shelf that occurred during the late Middle Pleistocene (between 135 to 125 ka), when the sea level dropped about 150 m (van der Kaars 1991, Zheng and Lei 1999). The Sundaland was then covered partly by a savannah corridor, stretching from Thailand to the Lesser Sunda Islands (Morley and Flenley, 1987, Heaney 1991). This corridor served as a barrier to the dispersal of the rainforest-dependent species. However, the forest-dwelling mammals survived in rainforest refugia for a while before reaching Java (van den Bergh et al. 2001).

On the other hand, the Khok Sung fauna lacks any evidence of taxa originating from Java. But the possible presence of *Duboisia santeng* in Tambun site (Peninsular Malaysia) may indicate the faunal exchange from Indonesia to the mainland Southeast Asia (Hooijer 1962, Medway 1972, Tougard 2001). *Duboisia santeng* is described from the early Middle Pleistocene of Kedung Brubus and the middle Middle Pleistocene of Trinil H. K. (Hooijer 1958). This taxon presumably arrived on the island of Java via the Siva-Malayan route (von Koenigswald 1935, Tougard 2001). The poor record or absence of the Indonesian taxa in mainland Southeast Asia is likely due to the disappearance of the land bridge during the interglacial phase. This acted as a sea barrier that did not facilitate insular mammals to migrate out of the islands.

The Khok Sung mammalian assemblage supports that Thailand was a biogeographic gateway of the Sino-Malayan migration event as the mainland forested faunal association replaced the earlier Siva-Malayan fauna (*Stegodon*-*Homo erectus* complex) subsequently in Java (von Koenigswald 1938, de Vos 1995). The glacial episodes are likely a key factor of southward onland dispersal of large mammals via the Sunda shelf. In addition, the occurrence of the Khok Sung reptiles is not truly representative of the early Siva-Malayan refugees but represents practically long-term survivors (e.g., *Crocodylus siamensis*, *Heosemys annandalii*, and *Heosemys grandis*) that evidently continued to exist up until today in Thailand.

## Supplementary Material

XML Treatment for Macaca sp.

XML Treatment for Cuon sp.

XML Treatment for Stegodon cf. orientalis

XML Treatment for Elephas sp.

XML Treatment for Rhinoceros sondaicus

XML Treatment for Rhinoceros unicornis

XML Treatment for Sus barbatus

XML Treatment for Axis axis

XML Treatment for Panolia eldii

XML Treatment for Rusa unicolor

XML Treatment for Bos sauveli

XML Treatment for Bos gaurus

XML Treatment for Bubalus arnee

XML Treatment for Capricornis sumatraensis

XML Treatment for Crocodylus cf. siamensis

XML Treatment for Python sp.

XML Treatment for Varanus sp.

## Appendices

**Appendix 1.** Measurements (in millimeters) of postcranial remains of identified mammal taxa from Khok Sung. * indicates a juvenile individual.

**Scapula**															
**Specimen**	**Taxa**	**GLP**	**LG**	**SLC**	**BG**	**Ld**	**DHA**	**HS**							
DMR KS-05-03-00-58	*Rhinoceros sondaicus*	87.49	78.81	62.97	55.33	144.77	–	325.57							
DMR-KS-05-03-26-2	*Bubalus arnee*	106.72	81.99	76.45	68.40	297.28	381.27	445.28							
DMR-KS-05-02-20-4	*Bubalus arnee*	84.00	66.89	52.13	54.50	213.22	301.79	296.17							
DMR-KS-05-06-24-4	*Panolia eldii*	44.00	34.56	23.41	31.54	108.17	206.05	211.37							
**Humerus**															
**Specimen**	**Taxa**	**Bp**	**Dp**	**Bd**	**BT**	**Dd**	**SD**	**GLC**	**GLl**	**GL**					
DMR-KS-05-03-10-6	Stegodon cf. orientalis	–	–	195.27	173.98	162.73	–	–	–	–					
DMR-KS-05-03-31-3	*Rhinoceros sondaicus*	174.58	142.51	155.86	94.39	115.77	66.09	377.04	422.18	437.23					
DMR-KS-05-03-26-8	*Sus barbatus*	65.67	76.44	57.39	47.06	45.11	20.72	224.71	236.13	238.28					
DMR-KS-05-03-20-2(1)	*Bos sauveli*	> 99.80	–	88.14	84.92	88.80	38.86	–	–	–					
DMR-KS-05-03-00-62	*Bos gaurus*	> 99.45	> 98.85	103.69	98.14	96.59	50.36	328.18	347.28	353.87					
DMR-KS-05-05-1-1	*Bos gaurus*	122.37	130.12	103.54	98.77	97.16	56.37	317.26	354.28	356.28					
DMR-KS-05-03-31-1	*Bubalus arnee*	125.53	135.07	104.40	101.70	93.72	52.29	343.74	413.22	444.12					
DMR-KS-05-03-31-8	*Bubalus arnee*	125.79	138.19	104.38	99.09	94.07	52.92	347.41	424.77	449.11					
DMR-KS-05-03-13-4	*Axis axis*	–	–	34.75	31.96	34.12	< 17.02	–	–	–					
DMR-KS-05-04-11-32	*Axis axis*	–	–	35.98	33.77	34.05	17.37	–	–	–					
DMR-KS-05-03-17-17	*Axis axis*	–	–	36.86	35.57	32.77	16.10	–	–	–					
DMR-KS-05-04-11-35	*Panolia eldii*	–	> 59.10	46.10	40.85	41.18	23.3	187.53	–	–					
DMR-KS-05-03-18-1	*Panolia eldii*	53.34	65.34	–	–	–	21.45	–	–	–					
DMR-KS-05-03-15-43	*Rusa unicolor*	–	–	54.89	49.04	48.30	< 27.70	–	–	–					
**Ulna and radius**															
**Specimen**	**Taxa**	**Bp/BPC**	**BFp**	**Dp**	**Bd**	**BFd**	**Dd**	**LO**	**DPA**	**SDO**	**SD**	**PL**	**Ll**	**GLl**	**GL**
DMR-KS-05-03-00-61	*Bubalus arnee*	106.16	92.17	52.99	100.36	90.17	65.85	125.53	93.64	72.61	55.54	305.18	308.75	476.61	484.72
DMR-KS-05-03-31-2	*Bubalus arnee*	108.45	98.91	57.03	103.37	92.25	73.75	131.15	98.37	76.03	50.74	335.29	343.51	427.78	452.17
DMR-KS-05-03-31-9	*Bubalus arnee*	106.69	97.99	57.44	103.49	92.06	72.92	128.89	99.00	76.54	47.66	335.93	344.77	424.77	449.11
DMR-KS-05-03-31-10	*Panolia eldii*	39.40	36.72	20.95	37.22	32.67	21.37	–	–	–	22.52	199.23	198.21	–	197.52
DMR-KS-05-04-11-3	*Panolia eldii*	42.30	38.22	22.47	36.84	34.64	23.46	–	–	–	22.61	204.18	203.71	–	215.70
DMR-KS-05-03-19-16	*Panolia eldii*	40.16	33.03	22.16	40.62	39.21	29.89	–	–	–	23.58	197.89	193.72	–	204.53
DMR-KS-05-03-25-9	*Rusa unicolor*	55.06	53.58	28.92	–	–	–	–	–	–	–	–	–	–	–
DMR-KS-05-03-19-14	*Rusa unicolor*	52.37	50.16	30.83	–	–	–	–	–	–	28.12	–	–	–	–
DMR-KS-05-03-26-19	*Rusa unicolor*	–	–	–	43.12	41.08	32.20	–	–	–	24.83	–	–	–	–
**Pelvis**															
**Specimen**	**Taxa**	**GL**	**LA**	**LS**	**SH**	**SB**	**SC**	**LFo**	**GBTc**	**GBA**	**GBTi**	**SBI**			
DMR-KS-05-03-10-11	Stegodon cf. orientalis	> 855.79	148.93	–	139.18	66.29	399.46	206.77	–	–	–	–			
DMR-KS-05-03-10-12	Stegodon cf. orientalis	> 496.75	140.75	–	145.17	59.60	389.92	201.25	–	–	–	–			
DMR-KS-05-04-1-25	*Bubalus arnee*	494.85	96.57	149.23	70.55	35.81	256.67	103.24	517.48	303.98	319.25	204.37			
**Femur**															
**Specimen**	**Taxa**	**Bp**	**Dp**	**DC**	**Bd**	**Dd**	**SD**	**GLC**	**GL**						
DMR-KS-05-03-10-4	Stegodon cf. orientalis	–	–	125.14	178.16	211.72	126.18	–	–						
DMR-KS-05-03-00-63	*Rhinoceros unicornis*	–	–	–	140.58	183.17	46.21	–	–						
DMR-KS-05-03-9-2	*Bos gaurus*	124.57	66.64	52.31	106.89	138.24	46.15	405.57	419.28						
DMR-KS-05-04-30-1	*Bos gaurus*	150.06	> 65.51	59.63	–	–	–	–	–						
DMR-KS-05-04-1-1	*Bubalus arnee*	160.51	80.69	66.76	128.54	156.98	54.05	420.18	447.15						
DMR-KS-05-04-1-2	*Bubalus arnee*	165.98	84.20	67.30	130.92	156.78	55.12	425.66	442.68						
DMR-KS-05-03-20-8	*Bubalus arnee*	–	–	–	124.57	181.53	–	–	–						
DMR-KS-05-03-27-4	*Axis axis*	–	–	–	50.49	69.42	–	–	–						
DMR-KS-05-03-27-11	*Panolia eldii*	66.29	31.42	28.4	52.67	70.12	21.97	–	–						
DMR-KS-05-03-17-36	*Panolia eldii*	68.57	35.49	28.73	56.56	73.06	21.97	251.58	–						
DMR-KS-05-03-28-20	*Panolia eldii*	–	–	–	53.72	72.00	–	–	–						
DMR-KS-05-04-05-38	*Panolia eldii*	67.59	35.21	27.74	–	–	–	–	–						
DMR-KS-05-03-00-119	*Panolia eldii*	–	–	–	51.60	71.30	< 22.94	–	–						
DMR-KS-05-03-19-2	*Panolia eldii*	–	–	–	51.85	73.23	< 26.51	–	–						
DMR-KS-05-08-16-1	*Panolia eldii*	59.40	32.32	26.14	–	–	–	–	–						
DMR-KS-05-04-11-2	*Rusa unicolor*	–	–	–	55.20	69.90	22.54	–	–						
DMR-KS-05-03-19-7	*Rusa unicolor*	67.01	44.50	28.58	–	–	21.72	–	–						
DMR-KS-05-03-12-2*	*Rusa unicolor*	48.57	31.96	–	–	–	18.91	–	–						
DMR-KS-05-03-26-5	*Rusa unicolor*	> 77.29	42.06	37.82	–	–	30.98	–	–						
DMR-KS-05-04-30-9	*Rusa unicolor*	–	–	–	54.46	69.12	21.85	–	–						
DMR-KS-05-04-19-10	*Rusa unicolor*	–	–	–	48.39	53.48	23.49	–	–						
**Tibia**															
**Specimen**	**Taxa**	**Bp**	**Dp**	**Bd**	**Dd**	**SD**	**Ll**	**GL**							
DMR-KS-05-03-00-52	*Rhinoceros sondaicus*	–	–	94.95	> 74.11	–	–	–							
DMR-KS-05-04-1-11	*Bubalus arnee*	132.04	125.08	87.26	69.54	55.84	363.36	437.17							
DMR-KS-05-04-1-3	*Bubalus arnee*	128.45	121.16	86.15	68.95	52.64	385.14	415.56							
DMR-KS-05-03-20-9	*Bubalus arnee*	126.74	106.22	88.37	64.62	53.42	354.45	406.77							
DMR-KS-05-03-28-16	*Rusa unicolor*	79.37	72.45	47.53	37.44	30.61	300.01	317.52							
DMR-KS-05-04-04-1	*Macaca* sp.	27.50	21.46	18.12	12.95	8.77	158.71	167.57							
**Fibula**															
**Specimen**	**Taxa**	**GL**													
DMR-KS-05-03-00-124	Stegodon cf. orientalis	> 354.56													
**Metacarpus**															
**Specimen**	**Taxa**	**Bp**	**Dp**	**Bd**	**Dd**	**DD**	**SD**	**Ll**	**GLl**	**GL**					
DMR-KS-05-03-28-29	*Rhinoceros sondaicus*	53.45	42.75	48.53	39.70	–	40.32	–	–	152.50					
DMR-KS-05-03-22-49	*Rhinoceros sondaicus*	–	–	51.80	34.10	22.26	–	–	–	–					
DMR-KS-05-04-05-15	*Rhinoceros sondaicus*	49.18	41.15	37.64	36.51	25.58	31.19	136.96	138.35	141.26					
DMR-KS-05-03-26-27	*Bos gaurus*	70.18	46.08	64.78	33.53	30.97	43.42	247.28	252.75	256.78					
DMR-KS-05-03-26-3(1)	*Bubalus arnee*	77.07	49.58	78.33	42.45	30.17	52.02	189.78	197.23	206.75					
DMR-KS-05-03-18-2	*Axis axis*	25.80	16.79	25.06	15.89	11.69	14.50	160.18	162.37	167.66					
DMR-KS-05-03-22-28	*Axis axis*	–	19.82	27.91	18.35	14.76	17.61	186.70	187.90	190.10					
DMR-KS-05-03-08-2	*Axis axis*	29.68	21.47	30.56	18.54	16.46	16.38	186.68	187.11	188.81					
DMR-KS-05-03-19-3	*Axis axis*	29.27	21.25	30.59	18.49	15.60	19.37	188.77	191.11	192.58					
DMR-KS-05-03-19-37	*Axis axis*	23.45	15.14	24.46	15.56	12.74	12.03	163.43	165.54	167.32					
DMR-KS-05-04-30-20	*Axis axis*	28.80	19.11	–	–	–	14.22	195.55	196.17	197.89					
DMR-KS-05-03-24-2	*Panolia eldii*	30.95	22.01	30.11	19.01	14.96	17.63	192.33	194.14	197.83					
DMR-KS-05-03-17-26	*Rusa unicolor*	37.58	29.64	37.00	24.64	19.31	24.38	216.98	217.35	224.25					
**Metatarsus**															
**Specimen**	**Taxa**	**Bp**	**Dp**	**Bd**	**Dd**	**DD**	**SD**	**Ll**	**GLl**	**GL**					
DMR-KS-05-04-1-8	*Bubalus arnee*	66.22	54.89	79.59	45.49	38.42	44.58	237.44	241.11	251.92					
DMR-KS-05-04-1-6	*Bubalus arnee*	65.79	58.19	82.22	45.07	38.88	44.44	239.66	241.52	255.33					
DMR-KS-05-03-28-30	*Bubalus arnee*	55.89	55.48	69.85	37.24	35.40	39.31	225.71	229.18	237.54					
DMR-KS-05-03-26-3	*Axis axis*	25.54	26.65	26.37	20.11	16.67	15.61	176.37	180.01	184.21					
DMR-KS-05-03-15-14	*Axis axis*	32.87	30.52	36.54	24.38	20.48	21.48	219.75	220.21	224.81					
DMR-KS-05-03-29-30	*Axis axis*	27.10	27.45	28.73	21.81	16.14	16.88	183.77	184.96	187.21					
DMR-KS-05-03-28-17	*Panolia eldii*	27.78	29.00	28.93	19.12	17.32	17.75	217.28	219.89	223.54					
DMR-KS-05-03-25-8	*Panolia eldii*	28.90	30.23	31.26	19.51	18.51	20.04	221.45	224.13	225.71					
DMR-KS-05-03-15-15	*Panolia eldii*	28.12	30.52	30.82	20.11	17.01	17.80	218.79	221.74	227.14					
DMR-KS-05-03-19-11	*Rusa unicolor*	36.89	35.69	37.75	26.63	22.36	24.08	233.75	238.89	244.97					

**Appendix 2.** Measurements (in millimeters) of mandibles of rhinoceroses from Khok Sung.

Taxon	*Rhinoceros sondaicus*		*Rhinoceros unicornis*
Mandible no.	DMR-KS-05-03-00-126	DMR-KS-05-03-31-28	DMR-KS-05-03-17-13
**Metrical parameters (mm)**			
Length of the mandible	> 311.0	> 198.3	> 404.1
Length of the mandibular symphysis	> 108.1	124.2	–
Width of the mandibular symphysis	–	59.5	–
Length of the diastema	–	50.5	–
Height of the mandibular corpus below the p2	51.7 (right)	44.5 (right)	–
	–	41.0 (left)	–
Height of the mandibular corpus below the p3	56.1 (right)	–	–
	50.1 (left)	46.8 (left)	–
Height of the mandibular corpus below the p4	76.2 (right)	–	–
	74.5 (left)	–	–
Height of the mandibular corpus below the m1	80.7 (right)	–	–
	84.2 (leftt)	–	–
Height of the mandibular corpus below the m2	94.0 (right)	–	–
	91.9 (leftt)	–	–
Height of the mandibular corpus below the m3	97.6 (right)	–	–
	92.4 (left)	–	126.6 (left)
Width of the mandibular corpus below the m1	55.3 (right)	–	–
	54.2 (left)	–	–
Width of the mandibular corpus below the m2	57.7 (right)	–	–
	57.5 (left)	–	–
Width of the mandibular corpus below the m3	57.3 (right)	–	–
	56.4 (left)	–	–

**Appendix 3.** Measurements (in millimeters) of mandible of *Sus barbatus* from Khok Sung. Numbers within the parentheses refer to the numbers used in von den Driesch’s metrical methods (1976: fig. 22b).

Taxon	*Sus barbatus*	
Mandible no.	DMR-KS-05-03-15-1	DMR-KS-05-04-19-1
**Metrical parameters (mm)**	male	female
Minimum length of the mandible	189.0	207.0
Diastema between c1 and p1	8.6	5.8
Diastema between p1 and p2	13.9	6.5
(9) Length of the premolar row, p1–p4	58.2 (left)	53.4 (right) 60.1 (left)
(9a) Length of the premolar row, p2–p4	41.4 (left)	38.4 (right) 39.0 (left)
(8) Length of the molar row	–	75.3 (right)
(7a) Length of the cheek tooth row, p2–m3	–	112.4 (right)
(7) Length of the cheek tooth row, p1–m3	–	126.3 (right)
(4) Length of the horizontal ramus: aboral border of the alveolus of m3 to infradentale	–	163.4 (right)
(6) Length: aboral border of the alveolus of m3 to aboral border of the canine alveolus	–	133.2 (right)
(11) Length: oral border of the alveolus of p2 to aboral border of the alveolus of i3	45.1 (right) 45.9 (left)	39.4 (right) 39.2 (left)
(12) Length of the median section of the body of mandible: from the mental prominence to infradentale	64,9 (right) 64.7 (left)	58.8 (right)
(16c) Height of the mandible in front of p2	50.9 (right) 50.4 (left)	41.5 (right)
(16b) Height of the mandible in front of m1	48.5 (left)	43.3 (right)
(16a) Height of the mandible in front of m3	–	45.1 (right)

**Appendix 4.** Measurements (in millimeters) of crania of cervids from Khok Sung. Numbers within the parentheses refer to the numbers used in von den Driesch’s metrical methods (1976: fig.11). * indicates measurements of the maximum length of the preservation according to incomplete specimens. “es” refers to an estimated value of the full length due to incomplete specimens.

Taxon	*Axis axis*				*Panolia eldii*
Cranium no.	DMR-KS-05-03-00-30	DMR-KS-05-04-18-50	DMR-KS-05-03-18-X9	DMR-KS-05-03-27-1	DMR-KS-05-04-20-4
**Metrical parameters (mm)**					
(6) Basicranial axis: Basion–Synsphenion	68.11	76.94	70.95	58.65*	93.34
(8) Neurocranium length: Basion–Nasion	–	–	–	143.64*	–
(10) Median frontal length: Akrokranion–Nasion	–	–	–	148.51*	–
(23) Greatest inner length of the orbit: Ectorbitale–Entorbitale	–	–	48.76 (right)	47.26 (left)	–
(25) Greatest mastoid breadth: Otion–Otion	100.94	92.35	93.29	96.29	98.28
(26) Greatest breadth of the occipital condyles	55.28	50.66	51.42	52.35	57.13
(27) Greatest breadth at the bases of the paraoccipital processes	83.89	78.94	80.24	88.51*	83.49
(28) Greatest breadth of the foramen magnum	22.53	25.15	22.65	24.83	26.20
(31) Least frontal breadth = least breadth of the forehead aboral of the orbits	88.42	99.22	–	99.06	81.08
(32) Greatest breadth across the orbits = greatest frontal breadth = nearly greatest breadth of skull: Ectorbitale–Ectorbitale	–	–	–	124.03*	–
(38) Basion–the highest point of the superior nuchal crest	62.62	61.91	62.65	63.52	66.97
(40) Proximal circumference of the burr = circumference of the distal end of the pedicle	116.2 (right), 114.50 (left)	104.56 (right), 104.81 (left)	111.87 (right), 103.59 (left)	100 (es) (right and left)	85.13 (right)
(41) Distal circumference of the burr	129.80 (left)	146.53 (right), 144.53 (left)	–	–	88.51* (right)

**Appendix 5.** Measurements (in millimeters) of mandibles of cervids from Khok Sung. Numbers within the parentheses refer to the numbers used in von den Driesch’s metrical methods (1976: fig. 21).

**Taxon**	***Axis axis***								***Panolia eldii***	
**Mandible no.**	DMR-KS-05-03-19-2	DMR-KS-05-03-19-1	DMR-KS-05-03-22-8	DMR-KS-05-04-01-1	DMR-KS-05-03-23-1	DMR-KS-05-03-29-1	DMR-KS-05-04-7-10	DMR-KS-05-03-26-10	DMR-KS-05-03-27-2	DMR-KS-05-04-9-5
**Metrical parameters (mm)**							Juvenile			
(3) Length: Gonion caudale–aboral border of the alveolus of m3	–	59.47	–	–	–	–	–	–	–	–
(5) Length: Gonion caudale–oral border of the alveolus of p2	–	149.42	–	–	–	–	–	–	–	–
(7) Length of the cheek tooth row, measured along the alveoli on the buccal side	–	–	–	80.53	88.11	84.49	–	–	89.82	–
(8) Length of the molar row, measured along the alveoli on the buccal side	–	56.09	–	54.29	54.22	54.20	–	–	54.83	–
(9) Length of the premolar row, measured along the alveoli on the buccal side	–	31.24	–	28.59	34.18	30.34	–	28.85	34.91	39.01
(11) Length of the diastema: oral border of the alveolus of p2–aboral border of the alveolus of i4	–	–	–	–	58.40	60.02	–	68.60	–	–
(12) Aboral height of the vertical ramus: Gonion ventrale–highest point of the condyle process	–	85.87	–	–	–	–	–	–	–	–
(13) Middle height of the vertical ramus: Gonion ventrale–deepest point of the mandibular notch	–	79.22	–	–	–	–	–	–	–	–
(15a) Height of the mandible behind m3 from the most aboral point of the alveolus on the buccal side	31.99	33.60	34.35	29.03	36.02	35.60	31.14	–	38.84	–
(15b) Height of the mandible in front of m1	–	26.72	–	–	29.74	26.01	–	28.85	31.13	–
(15c) Height of the mandible in front of p2	–	22.13	–	24.11	21.43	21.57	16.28	23.02	27.10	–
**Taxon**	***Axis axis***								***Rusa unicolor***	
**Mandible no.**	DMR-KS–05–03–18–22	DMR-KS–05–03–20–1	DMR-KS–05–03–22–7	DMR-KS–05–03–22–6	DMR-KS–05–04–03–1	DMR-KS–05–03–27–22	DMR-KS–05–03–27–3	DMR-KS–05–04–09–2	DMR-KS–05–03–00–101	DMR-KS–05–03–13
(3) Length: Gonion caudale–aboral border of the alveolus of m3	–	–	–	–	–	–	–	59.53	–	–
(7) Length of the cheek tooth row, measured along the alveoli on the buccal side	–	–	–	–	95.21	–	–	–	–	–
(8) Length of the molar row, measured along the alveoli on the buccal side	–	53.17	–	–	61.52	–	–	51.78	68.51	78.11
(9) Length of the premolar row, measured along the alveoli on the buccal side	–	–	–	–	35.88	–	–	–	–	–
(11) Length of the diastema: oral border of the alveolus of p2–aboral border of the alveolus of i4	–	–	–	–	58.91	–	–	–	–	–
(12) Aboral height of the vertical ramus: Gonion ventrale–highest point of the condyle process	–	–	–	–	–	–	–	88.50	–	–
(13) Middle height of the vertical ramus: Gonion ventrale–deepest point of the mandibular notch	–	–	–	–	–	–	–	84.79	–	–
(14) Oral height of the vertical ramus: Gonion ventrale–Coronion	–	–	–	–	–	–	–	125.12	–	–
(15a) Height of the mandible behind m3 from the most aboral point of the alveolus on the buccal side	–	–	36.92	–	38.71	36.22	38.50	37.30	51.76	–
(15b) Height of the mandible in front of m1	–	–	–	28.58	24.35	32.29	–	31.76	–	–
(15c) Height of the mandible in front of p2	23.21	–	–	–	19.72	–	–	–	–	–

**Appendix 6.** Measurements (in millimeters) of scapulae of extant ruminants from Southeast Asia.

	HS	DHA	Ld	SLC	GLP	LG	BG	HS/Ld	DHA/Ld	Ld/SLC	LG/BG	GLP/LG	SLC/BG
***Axis axis*** (N=6)													
Max	176.10	164.20	98.30	21.42	38.84	30.11	25.46	1.82	1.72	4.70	1.25	1.42	0.85
Min	157.80	152.10	91.20	19.39	34.32	25.50	23.66	1.73	1.58	4.51	1.02	1.19	0.82
Mean	168.62	157.47	95.18	20.75	36.60	27.83	24.69	1.77	1.65	4.59	1.13	1.32	0.84
***Axis porcinus*** (N=2)													
Max	145.40	134.10	80.90	17.73	31.45	23.58	23.82	1.80	1.66	4.61	1.05	1.35	0.79
Min	143.60	133.20	80.80	17.54	30.20	22.41	22.55	1.78	1.65	4.56	0.94	1.33	0.74
Mean	144.50	133.65	80.85	17.64	30.83	23.00	23.19	1.79	1.65	4.58	0.99	1.34	0.76
***Panolia eldii*** (N=4)													
Max	228.90	210.10	118.50	28.07	42.45	34.93	32.25	1.93	1.77	4.86	1.15	1.28	0.87
Min	200.20	178.90	109.31	22.63	39.75	31.07	27.75	1.82	1.63	4.22	1.07	1.22	0.82
Mean	214.65	194.53	114.04	25.38	41.16	33.07	29.99	1.88	1.70	4.53	1.10	1.25	0.84
***Rusa unicolor*** (N=4)													
Max	269.40	274.70	167.40	38.43	61.87	47.23	45.60	1.62	1.66	4.43	1.08	1.32	0.93
Min	233.10	235.10	152.70	34.69	52.37	40.50	37.66	1.52	1.54	4.36	1.01	1.29	0.83
Mean	250.93	254.73	159.78	36.38	56.94	43.64	41.50	1.57	1.59	4.39	1.05	1.30	0.88
***Bos sauveli*** (N=4)													
Max	389.00	359.70	216.50	66.42	73.22	62.72	56.78	2.25	2.12	3.28	1.13	1.17	1.18
Min	381.20	357.80	169.60	52.38	65.48	56.54	50.25	1.80	1.65	3.23	1.10	1.15	1.03
Mean	385.08	358.85	193.08	59.31	69.41	59.59	53.55	2.02	1.89	3.25	1.11	1.16	1.10
***Bos javanicus*** (N=6)													
Max	441.90	381.60	226.80	62.95	77.54	65.88	61.83	1.95	1.71	3.69	1.18	1.19	1.03
Min	384.30	339.10	198.20	53.91	71.11	61.66	52.24	1.83	1.65	3.60	1.07	1.15	1.01
Mean	403.53	355.58	211.47	57.94	74.20	63.41	56.79	1.91	1.68	3.65	1.12	1.17	1.02
***Bos gaurus*** (N=6)													
Max	536.10	491.20	258.70	71.23	90.16	80.07	70.45	2.07	1.90	3.72	1.19	1.20	1.20
Min	393.70	356.70	191.30	54.34	79.07	66.08	55.65	1.87	1.86	3.52	1.14	1.13	0.91
Mean	462.95	434.45	230.78	63.76	83.68	72.37	62.47	2.01	1.88	3.61	1.16	1.16	1.02
***Bubalus arnee*** (N=6)													
Max	374.00	362.80	238.10	71.55	81.72	65.77	55.71	1.66	1.52	4.29	1.39	1.31	1.50
Min	320.90	303.70	207.60	53.11	74.22	62.38	45.02	1.41	1.34	3.07	1.17	1.16	1.04
Mean	346.50	327.02	223.60	64.02	79.49	64.20	50.60	1.55	1.46	3.55	1.28	1.24	1.27

**Appendix 7.** Measurements (in millimeters) of humeri of extant ruminants from Southeast Asia.

	Bp	Dp	Bd	Dd	SD	GL	BT	GLC	GLl	GL/Bp	GL/Dp	GL/Bd	GL/Dd	Bp/Bd	Dp/Dd	Bp/Dp	Bd/Dd	Bd/BT
***Axis axis*** (N=8)																		
Max	48.01	55.42	41.82	36.58	18.48	200.30	36.44	177.10	196.80	4.20	3.77	5.13	5.67	1.30	1.68	0.91	1.23	1.17
Min	42.12	47.92	33.98	30.63	15.21	172.40	31.87	153.10	167.30	3.84	3.16	4.59	5.16	1.11	1.46	0.80	1.04	1.07
Mean	48.16	44.50	35.57	24.98	98.52	107.03	98.24	169.66	90.14	3.77	4.16	5.10	3.29	1.37	1.20	1.00	1.12	1.12
***Axis porcinus*** (N=2)																		
Max	35.42	51.11	32.42	27.11	14.67	153.20	27.65	125.10	142.30	4.40	3.02	4.87	5.78	1.12	1.91	0.69	1.23	1.19
Min	34.74	50.53	31.49	26.43	14.55	152.80	27.34	124.30	141.80	4.33	3.00	4.71	5.65	1.07	1.89	0.69	1.16	1.14
Mean	35.08	50.82	31.96	26.77	14.61	153.00	27.50	124.70	142.05	4.36	3.01	4.79	5.72	1.10	1.90	0.69	1.19	1.16
***Panolia eldii*** (N=4)																		
Max	54.56	62.98	43.48	38.15	20.40	211.70	36.81	185.80	206.30	4.08	3.40	4.87	5.57	1.26	1.66	0.87	1.19	1.19
Min	47.17	56.65	42.52	35.75	18.52	192.30	36.02	171.30	190.40	3.88	3.35	4.51	5.35	1.11	1.57	0.83	1.14	1.16
Mean	50.94	59.78	43.06	36.97	19.46	202.00	36.53	178.53	198.45	3.97	3.38	4.69	5.46	1.18	1.62	0.85	1.17	1.18
***Rusa unicolor*** (N=4)																		
Max	73.14	85.10	66.12	46.33	27.62	277.80	61.23	252.40	267.90	3.98	3.54	4.81	6.22	1.21	1.91	0.89	1.48	1.14
Min	64.44	72.40	53.35	44.66	24.82	256.40	47.29	224.10	254.30	3.80	3.26	4.19	5.54	1.10	1.56	0.86	1.16	1.08
Mean	68.75	78.66	59.81	45.61	26.06	266.83	54.21	238.05	261.23	3.89	3.40	4.49	5.85	1.15	1.73	0.88	1.31	1.11
***Bos sauveli*** (N=4)																		
Max	105.62	104.67	90.28	84.60	43.08	338.10	86.30	306.10	333.70	3.28	3.23	3.86	4.17	1.18	1.41	1.01	1.08	1.07
Min	90.62	100.44	76.98	71.24	32.67	296.80	71.78	276.10	291.20	3.19	2.94	3.74	3.99	1.17	1.23	0.90	1.07	1.05
Mean	98.12	102.63	83.64	77.67	37.89	317.35	79.09	290.95	312.63	3.24	3.09	3.80	4.09	1.17	1.33	0.95	1.08	1.06
***Bos javanicus*** (N=6)																		
Max	111.54	137.34	102.41	79.42	43.75	354.40	83.12	291.30	318.40	3.48	3.00	3.82	4.48	1.12	1.74	0.95	1.29	1.23
Min	94.97	109.87	86.67	76.08	40.06	326.10	77.15	278.80	302.50	3.13	2.58	3.46	4.25	1.08	1.43	0.80	1.14	1.12
Mean	103.36	119.39	94.08	77.42	42.71	337.57	79.41	285.97	312.97	3.27	2.85	3.60	4.36	1.10	1.54	0.87	1.21	1.18
***Bos gaurus*** (N=6)																		
Max	135.46	154.89	110.89	96.12	56.49	395.40	99.32	353.80	391.50	2.99	3.46	3.82	4.20	1.28	1.61	1.22	1.15	1.13
Min	117.31	96.43	94.21	85.01	43.22	333.20	87.12	302.10	321.50	2.79	2.55	3.49	3.64	1.21	1.05	0.86	1.04	1.06
Mean	124.31	123.64	99.99	91.03	49.09	362.02	91.55	325.98	355.32	2.91	3.00	3.62	3.98	1.24	1.35	1.03	1.10	1.09
***Bubalus arnee*** (N=6)																		
Max	102.11	106.03	87.29	77.75	50.59	319.60	80.69	271.70	317.80	3.41	3.16	3.85	4.28	1.24	1.46	0.97	1.15	1.11
Min	92.71	101.13	82.33	72.58	38.44	310.50	73.89	262.10	305.40	3.04	2.93	3.66	4.07	1.10	1.33	0.88	1.06	1.04
Mean	97.08	103.83	83.98	75.75	42.93	315.92	77.86	266.88	312.07	3.26	3.04	3.76	4.17	1.16	1.37	0.94	1.11	1.08

**Appendix 8.** Measurements (in millimeters) of radii and ulnae of extant ruminants from Southeast Asia.

	Bp	Dp	Bd	Dd	SD	GL	GLl	BFd	BFp	BPC	PL	Ll	SDO	DPA	LO
***Axis axis*** (N=8)															
Max	37.72	18.84	34.78	26.20	23.88	241.10	236.80	29.93	33.07	20.72	171.20	177.70	30.96	34.01	49.28
Min	34.16	16.11	30.00	21.74	19.46	203.70	198.90	26.97	30.12	16.33	139.40	160.30	24.87	28.49	41.44
Mean	35.43	17.78	32.68	24.28	21.06	222.60	218.74	28.37	31.30	18.93	162.54	165.38	27.89	30.82	45.17
***Axis porcinus*** (N=4)															
Max	31.15	15.78	25.09	24.81	16.45	165.70	162.80	24.02	27.11	15.44	126.03	132.09	25.17	26.16	40.22
Min	28.45	13.37	24.11	16.43	12.81	164.10	158.40	22.73	25.85	13.66	122.60	119.42	20.45	23.94	36.03
Mean	29.80	14.36	24.65	20.72	14.71	165.08	160.58	23.29	26.30	14.64	124.52	125.71	22.97	25.17	37.99
***Panolia eldii*** (N=3)															
Max	42.22	20.72	39.16	26.63	23.31	276.60	271.80	36.15	37.87	24.74	212.80	217.50	33.98	34.91	54.59
Min	38.88	19.02	35.18	25.16	19.93	254.80	249.20	33.17	35.14	20.32	196.70	200.10	30.69	33.48	46.71
Mean	40.04	19.85	36.68	25.80	21.08	262.30	256.90	34.20	36.07	21.84	202.20	206.07	31.82	34.09	49.43
***Rusa unicolor*** (N=4)															
Max	59.02	31.24	54.29	40.62	32.49	322.50	320.50	46.82	54.38	29.77	239.80	243.60	46.89	50.60	70.12
Min	51.89	24.21	44.25	34.29	29.10	296.30	291.30	41.82	46.02	23.81	217.80	222.20	39.89	42.37	57.71
Mean	55.35	27.73	49.20	37.52	30.66	309.45	305.83	44.26	50.07	26.34	228.53	232.90	43.18	46.18	63.96
***Bos sauveli*** (N=4)															
Max	89.11	38.72	83.52	58.24	42.13	372.30	364.50	74.01	78.29	45.63	316.50	314.20	51.33	64.95	109.67
Min	78.88	35.44	68.52	56.47	36.54	329.40	324.80	66.14	72.13	44.24	285.40	274.10	46.46	64.32	93.75
Mean	83.99	37.14	76.06	57.41	39.35	350.90	344.63	70.21	75.22	44.99	300.95	294.05	48.89	64.59	101.67
***Bos javanicus*** (N=6)															
Max	93.83	43.24	90.23	57.23	50.34	420.90	421.30	70.11	81.49	47.56	293.50	283.40	61.20	73.76	114.89
Min	82.22	39.12	75.30	47.26	43.74	389.20	372.40	65.35	76.32	44.59	285.10	281.30	50.30	71.04	103.98
Mean	88.05	41.32	82.89	52.20	46.17	402.75	393.83	68.04	79.14	45.96	288.23	282.52	54.78	71.99	109.80
***Bos gaurus*** (N=6)															
Max	107.54	56.43	103.42	69.94	60.98	470.60	461.70	88.76	96.43	58.43	345.60	328.60	67.68	90.12	137.40
Min	90.31	46.45	80.33	53.26	48.24	414.85	415.70	72.96	84.93	49.64	291.30	281.40	56.42	71.45	86.43
Mean	97.23	50.39	92.17	59.82	53.57	439.98	432.00	80.35	88.83	53.83	320.88	310.42	60.96	79.86	114.21
***Bubalus arnee*** (N=6)															
Max	86.89	45.55	81.67	64.24	51.84	411.20	385.40	78.73	76.52	50.66	293.50	292.60	60.43	78.24	112.34
Min	77.01	41.45	73.23	53.10	43.07	377.40	372.30	70.68	70.91	46.32	283.10	281.20	48.44	68.09	95.47
Mean	81.86	43.36	77.95	58.93	46.51	393.25	380.40	74.37	73.89	48.47	288.32	287.23	54.81	72.12	104.12
	**PL/Bp**	**PL/Dp**	**PL/Bd**	**PL/Dd**	**Bp/Bd**	**Dd/Dp**	**Bp/Dp**	**Bd/Dd**	**Bp/BFp**	**Bd/BFd**	**GL/LO**				
***Axis axis*** (N=8)															
Max	4.87	9.86	5.33	7.65	1.15	1.48	2.14	1.52	1.18	1.22	5.31				
Min	3.93	7.81	4.30	5.45	1.04	1.27	1.91	1.22	1.10	1.08	4.68				
Mean	4.59	9.16	4.98	6.73	1.09	1.37	2.00	1.35	1.13	1.15	4.93				
***Axis porcinus*** (N=4)															
Max	4.42	9.43	5.13	7.53	1.29	1.86	2.17	1.47	1.16	1.09	4.59				
Min	3.97	7.77	4.89	5.08	1.16	1.08	1.94	1.00	1.10	1.00	4.11				
Mean	4.19	8.72	5.05	6.23	1.21	1.46	2.08	1.23	1.13	1.06	4.36				
***Panolia eldii*** (N=3)															
Max	5.06	10.34	5.59	7.99	1.11	1.32	2.04	1.47	1.11	1.08	5.47				
Min	5.04	9.95	5.43	7.70	1.08	1.29	1.97	1.39	1.11	1.06	5.07				
Mean	5.05	10.19	5.51	7.84	1.09	1.30	2.02	1.42	1.11	1.07	5.32				
***Rusa unicolor*** (N=4)															
Max	4.20	9.01	4.92	6.35	1.18	1.43	2.14	1.34	1.14	1.17	5.15				
Min	4.04	7.63	4.42	5.87	1.07	1.30	1.88	1.28	1.08	1.06	4.60				
Mean	4.13	8.32	4.67	6.11	1.13	1.36	2.01	1.31	1.11	1.11	4.86				
***Bos sauveli*** (N=4)															
Max	3.62	8.93	4.17	5.60	1.15	1.59	2.51	1.48	1.14	1.13	3.97				
Min	3.55	7.38	3.79	4.90	1.06	1.50	2.04	1.18	1.09	1.03	3.01				
Mean	3.59	8.13	3.97	5.25	1.11	1.55	2.27	1.33	1.12	1.08	3.49				
***Bos javanicus*** (N=6)															
Max	3.49	7.32	3.81	6.16	1.11	1.46	2.17	1.78	1.15	1.29	3.74				
Min	3.13	6.75	3.18	5.01	0.99	1.10	2.10	1.32	1.07	1.15	3.59				
Mean	3.28	6.99	3.49	5.56	1.06	1.27	2.13	1.60	1.11	1.22	3.67				
***Bos gaurus*** (N=6)															
Max	3.58	6.97	4.03	6.08	1.12	1.25	1.96	1.64	1.12	1.17	4.80				
Min	3.13	5.99	3.18	4.94	1.01	1.14	1.90	1.48	1.06	1.09	3.42				
Mean	3.31	6.38	3.51	5.40	1.06	1.18	1.93	1.54	1.09	1.15	3.96				
***Bubalus arnee*** (N=6)															
Max	3.68	6.84	3.87	5.39	1.06	1.55	1.95	1.48	1.17	1.07	3.96				
Min	3.38	6.44	3.59	4.41	1.03	1.21	1.84	1.14	1.07	1.04	3.50				
Mean	3.53	6.66	3.70	4.92	1.05	1.36	1.89	1.33	1.11	1.05	3.79				

**Appendix 9.** Measurements (in millimeters) of metacarpi of extant ruminants from Southeast Asia.

	Bp	Dp	Bd	Dd	SD	GL	GLl	Ll	DD	GL/Bp	GL/Dp	GL/Bd	GL/Dd	Bp/Bd	Dp/Dd	Bp/Dp	Bd/Dd
***Axis axis*** (N=8)																	
Max	28.75	19.36	28.03	17.75	18.20	174.30	172.80	169.00	17.55	6.52	9.77	7.69	12.69	1.26	1.36	1.59	1.88
Min	25.13	17.23	22.56	13.59	14.18	162.10	159.50	156.70	12.33	6.06	8.74	6.22	9.52	1.03	1.02	1.35	1.30
Mean	26.94	18.25	24.86	16.24	15.66	169.30	167.04	164.05	14.58	6.29	9.28	6.83	10.52	1.09	1.13	1.48	1.54
***Axis porcinus*** (N=4)																	
Max	23.14	15.71	23.60	14.61	14.18	120.80	116.88	118.82	11.76	5.68	7.92	5.81	8.82	1.06	1.14	1.59	1.76
Min	21.28	14.43	20.79	13.38	12.63	111.00	109.20	107.80	9.42	4.83	7.45	4.70	7.66	0.97	1.03	1.36	1.50
Mean	22.18	15.11	21.77	13.86	13.39	115.99	113.35	113.52	10.62	5.25	7.68	5.35	8.38	1.02	1.09	1.47	1.57
***Panolia eldii*** (N=4)																	
Max	34.01	20.89	29.98	20.66	18.08	224.80	222.50	221.30	17.58	7.18	11.33	7.82	11.20	1.18	1.06	1.72	1.49
Min	30.61	19.81	28.11	19.64	17.60	219.80	216.20	214.40	15.06	6.60	10.52	7.50	10.87	1.08	0.96	1.47	1.40
Mean	32.22	20.32	28.83	20.04	17.82	222.25	219.48	217.63	16.45	6.91	10.95	7.71	11.09	1.12	1.01	1.59	1.44
***Rusa unicolor*** (N=4)																	
Max	42.02	28.45	41.13	27.81	24.99	234.30	230.40	226.70	22.07	5.77	8.24	5.96	8.84	1.03	1.11	1.48	1.54
Min	36.43	26.66	35.35	24.08	22.19	210.20	205.10	203.50	19.54	5.55	7.83	5.70	8.43	1.02	1.02	1.36	1.46
Mean	39.22	27.58	38.11	25.60	23.42	221.95	217.60	214.95	20.69	5.67	8.04	5.83	8.68	1.03	1.08	1.42	1.49
***Bos sauveli*** (N=2)																	
Max	65.72	36.61	57.43	32.87	36.44	249.10	246.40	241.50	29.43	3.81	6.82	4.35	7.68	1.15	1.13	1.80	1.77
Min	65.44	36.54	57.09	32.45	36.23	248.40	245.30	241.20	29.22	3.78	6.79	4.34	7.56	1.14	1.11	1.79	1.74
Mean	65.58	36.58	57.26	32.66	36.34	248.75	245.85	241.35	29.33	3.79	6.80	4.34	7.62	1.15	1.12	1.79	1.75
***Bos javanicus*** (N=6)																	
Max	66.76	40.44	60.38	39.84	39.86	225.20	214.30	215.10	27.71	3.76	6.46	4.09	6.86	1.11	1.16	1.74	1.69
Min	59.28	34.48	54.45	32.45	34.21	215.30	206.10	205.70	26.03	3.37	5.53	3.73	5.65	1.09	1.01	1.58	1.51
Mean	62.88	37.73	57.30	35.67	37.25	222.10	212.44	211.66	26.84	3.54	5.92	3.88	6.27	1.10	1.06	1.67	1.61
***Bos gaurus*** (N=6)																	
Max	75.52	43.88	69.11	40.81	51.55	251.50	247.20	239.10	33.49	3.78	6.24	4.14	6.61	1.12	1.11	1.76	1.75
Min	66.25	39.22	60.68	37.86	39.04	226.50	218.20	215.10	28.44	3.28	5.73	3.35	5.87	1.02	1.02	1.60	1.59
Mean	70.36	41.52	65.83	39.08	44.16	242.95	235.85	230.37	31.13	3.46	5.85	3.70	6.22	1.07	1.06	1.70	1.68
***Bubalus arnee*** (N=6)																	
Max	68.11	42.38	74.01	38.81	45.12	195.20	186.60	180.60	28.92	2.97	6.08	2.79	5.43	0.97	1.10	2.12	1.98
Min	61.63	31.14	66.52	33.77	30.97	182.80	170.70	172.40	24.32	2.87	4.57	2.61	4.99	0.91	0.85	1.58	1.91
Mean	65.18	33.89	69.73	35.89	36.91	189.60	178.42	176.14	25.74	2.91	5.66	2.72	5.29	0.93	0.94	1.94	1.94

**Appendix 10.** Measurements (in millimeters) of femora of extant ruminants from Southeast Asia.

	Bp	Dp	Bd	Dd	SD	GL	GLC	DC	GL/Bp	GL/Dp	GL/Bd	GL/Dd	Bp/Bd	Dd/Dp	Bp/Dp	Dd/Bd
***Axis axis*** (N=6)																
Max	55.93	27.30	47.01	65.81	18.53	219.70	209.20	24.02	4.24	8.93	4.94	3.52	1.26	2.56	2.15	1.49
Min	49.76	23.63	43.72	59.88	17.31	210.90	198.20	22.35	3.90	8.05	4.63	3.32	1.11	2.35	2.02	1.33
Mean	53.26	25.66	45.37	63.15	18.09	215.97	204.03	23.32	4.06	8.43	4.76	3.42	1.17	2.46	2.08	1.39
***Axis porcinus*** (N=2)																
Max	47.14	23.25	38.75	56.33	16.43	183.60	173.40	19.77	3.89	8.14	4.84	3.26	1.24	2.50	2.10	1.49
Min	46.71	22.26	37.91	55.71	16.04	181.20	172.70	19.36	3.88	7.90	4.68	3.25	1.21	2.42	2.03	1.44
Mean	46.93	22.76	38.33	56.02	16.24	182.40	173.05	19.57	3.89	8.02	4.76	3.26	1.22	2.46	2.06	1.46
***Panolia eldii*** (N=4)																
Max	66.06	30.81	52.98	73.65	22.92	267.70	253.10	26.77	4.05	8.83	5.06	3.63	1.25	2.43	2.18	1.44
Min	60.54	30.30	49.74	71.12	21.50	241.40	225.30	24.54	3.99	7.85	4.85	3.39	1.22	2.31	1.97	1.39
Mean	63.29	30.55	51.36	72.45	22.19	254.63	239.35	25.63	4.02	8.34	4.96	3.51	1.23	2.37	2.07	1.41
***Rusa unicolor*** (N=4)																
Max	91.36	44.39	76.39	102.41	32.56	354.80	336.20	38.43	4.27	8.15	4.75	4.18	1.21	2.35	2.08	1.34
Min	74.13	42.25	66.99	76.12	26.90	316.30	296.70	32.08	3.87	7.45	4.64	3.46	1.09	1.78	1.75	1.13
Mean	82.68	43.23	71.67	88.78	29.58	335.80	316.10	35.29	4.08	7.76	4.69	3.83	1.15	2.05	1.91	1.23
***Bos sauveli*** (N=4)																
Max	131.83	69.02	99.92	132.22	46.31	408.20	389.50	48.12	3.44	5.92	4.09	3.11	1.32	2.04	1.91	1.33
Min	106.60	62.94	96.44	128.62	31.30	366.20	347.10	46.27	3.10	5.79	3.80	2.85	1.11	1.90	1.69	1.31
Mean	118.88	66.04	98.18	130.18	38.61	387.23	368.30	47.09	3.27	5.86	3.94	2.97	1.21	1.97	1.80	1.33
***Bos javanicus*** (N=6)																
Max	134.88	72.44	105.98	135.67	48.37	427.30	395.10	55.28	3.30	6.12	4.09	3.17	1.32	1.94	1.98	1.30
Min	124.52	63.45	94.81	122.95	38.89	387.10	363.70	51.97	3.09	5.76	3.94	3.10	1.22	1.86	1.85	1.25
Mean	128.18	67.43	100.88	129.08	43.23	406.38	381.20	53.46	3.17	6.03	4.03	3.15	1.27	1.92	1.90	1.28
***Bos gaurus*** (N=6)																
Max	151.32	93.76	124.94	156.78	49.63	483.20	458.70	59.27	3.68	6.69	4.19	3.18	1.31	2.26	1.90	1.32
Min	115.19	63.46	108.35	142.42	43.55	423.70	428.70	37.88	3.19	5.15	3.77	2.96	1.04	1.67	1.61	1.25
Mean	135.93	77.41	115.22	147.53	45.77	452.82	440.95	54.42	3.35	5.94	3.93	3.07	1.18	1.94	1.77	1.28
***Bubalus arnee*** (N=6)																
Max	131.65	69.14	105.25	134.59	47.78	386.20	378.10	51.44	2.95	5.73	3.68	3.04	1.25	1.98	1.95	1.29
Min	123.63	67.17	101.98	125.25	39.52	353.70	336.50	49.23	2.86	5.22	3.46	2.81	1.21	1.86	1.82	1.21
Mean	128.81	67.82	104.06	129.11	42.99	374.52	361.50	50.72	2.91	5.52	3.60	2.90	1.24	1.90	1.90	1.24

**Appendix 11.** Measurements (in millimeters) of tibiae of extant ruminants from Southeast Asia.

	**Bp**	**Dp**	**Bd**	**Dd**	**SD**	**GL**	**Ll**	**GL/Bp**	**GL/Dp**	**GL/Bd**	**GL/Dd**	**Bp/Bd**	**Dp/Dd**	**Bp/Dp**	**Bd/Dd**
***Axis axis*** (N=6)															
Max	52.61	49.43	34.89	24.24	19.97	222.20	240.10	4.51	4.97	7.07	9.61	1.65	2.16	1.14	1.56
Min	48.92	42.77	30.03	22.11	17.98	209.60	226.40	4.09	4.44	6.17	9.14	1.45	1.89	0.99	1.33
Mean	50.12	46.99	32.48	23.06	18.62	216.52	232.87	4.32	4.62	6.68	9.39	1.55	2.04	1.07	1.41
***Axis porcinus*** (N=4)															
Max	43.79	43.73	28.22	22.21	17.11	207.90	198.30	4.77	5.18	7.88	9.42	1.66	2.08	1.08	1.35
Min	41.22	40.05	26.36	20.53	16.80	182.70	192.30	4.41	4.18	6.47	8.65	1.46	1.82	0.94	1.19
Mean	42.51	41.84	27.15	21.47	16.98	195.33	195.55	4.59	4.68	7.21	9.09	1.57	1.95	1.02	1.27
***Panolia eldii*** (N=4)															
Max	58.99	59.38	37.56	29.35	21.62	294.80	285.40	5.02	5.14	7.86	10.35	1.68	2.03	1.03	1.32
Min	58.01	57.40	34.96	28.46	20.65	274.30	258.00	4.66	4.62	7.83	9.36	1.56	2.01	0.98	1.19
Mean	58.63	58.29	36.27	28.88	21.09	284.65	271.75	4.85	4.89	7.85	9.86	1.62	2.02	1.01	1.26
***Rusa unicolor*** (N=4)															
Max	84.53	83.90	51.92	43.54	30.12	334.60	354.40	4.00	4.47	6.66	8.22	1.68	1.97	1.12	1.35
Min	73.20	65.43	47.48	35.53	28.83	291.90	316.80	3.96	3.97	6.06	7.68	1.53	1.83	0.99	1.15
Mean	78.67	74.36	49.45	39.37	29.44	313.13	335.33	3.98	4.24	6.33	7.97	1.59	1.88	1.06	1.26
***Bos sauveli*** (N=4)															
Max	102.24	92.12	73.59	49.56	44.92	398.30	364.90	3.90	4.55	5.82	8.20	1.55	1.86	1.17	1.51
Min	98.75	87.54	63.92	48.58	38.54	372.20	311.60	3.76	4.04	5.40	7.51	1.39	1.80	1.07	1.29
Mean	100.51	89.86	68.80	49.12	41.72	384.98	338.15	3.83	4.29	5.61	7.84	1.47	1.83	1.12	1.40
***Bos javanicus*** (N=6)															
Max	106.11	97.35	69.35	56.17	46.06	414.50	364.70	3.91	4.28	6.23	7.63	1.61	1.81	1.10	1.24
Min	100.05	96.02	64.88	53.01	38.60	385.30	354.80	3.83	3.96	5.80	6.86	1.51	1.73	1.03	1.18
Mean	103.61	96.78	66.86	55.09	42.05	401.23	360.73	3.87	4.15	6.00	7.29	1.55	1.76	1.07	1.21
***Bos gaurus*** (N=6)															
Max	130.17	107.70	75.51	60.10	57.06	449.70	396.30	3.66	4.22	5.99	7.81	1.73	1.87	1.21	1.31
Min	113.50	98.40	72.50	57.60	46.00	415.40	351.70	3.45	4.18	5.69	7.10	1.56	1.69	1.15	1.23
Mean	120.97	103.27	74.01	58.39	50.25	432.45	373.75	3.58	4.19	5.84	7.41	1.63	1.77	1.17	1.27
***Bubalus arnee*** (N=6)															
Max	107.80	103.58	71.90	53.37	48.92	367.20	340.50	3.53	4.40	5.30	7.07	1.56	1.99	1.25	1.44
Min	101.53	82.14	67.68	47.10	40.71	324.10	294.70	3.11	3.45	4.77	6.78	1.41	1.58	1.04	1.30
Mean	104.67	93.55	69.57	50.67	45.08	350.18	316.90	3.35	3.77	5.03	6.91	1.51	1.85	1.13	1.38

**Appendix 12.** Measurements (in millimeters) of metatarsi of extant ruminants from Southeast Asia.

	Bp	Dp	Bd	Dd	SD	GL	GLl	Ll	DD	GL/Bp	GL/Dp	GL/Bd	GL/Dd	Bp/Bd	Dp/Dd	Bp/Dp	Bd/Dd
***Axis axis*** (N=6)																	
Max	24.36	26.28	26.56	18.15	17.76	187.80	185.40	182.50	15.25	8.00	7.74	7.53	12.97	0.96	1.80	0.98	1.72
Min	23.38	24.26	24.86	14.43	13.97	180.10	179.60	175.50	13.48	7.50	6.95	6.91	10.06	0.90	1.42	0.90	1.40
Mean	23.81	25.46	25.78	16.72	15.32	184.22	182.05	179.68	14.31	7.74	7.24	7.15	11.12	0.92	1.53	0.94	1.55
***Axis porcinus*** (N=4)																	
Max	23.95	22.96	24.00	15.63	13.45	143.46	139.93	139.08	13.56	6.69	6.74	6.25	9.32	1.02	1.53	1.08	1.55
Min	21.44	21.27	22.94	15.01	12.37	130.90	129.40	128.30	12.56	5.48	5.70	5.52	8.47	0.93	1.37	1.01	1.48
Mean	22.70	21.96	23.32	15.41	13.08	137.47	134.71	133.73	13.15	6.09	6.28	5.90	8.92	0.97	1.43	1.03	1.51
***Panolia eldii*** (N=4)																	
Max	30.76	29.92	29.58	19.81	17.68	237.80	235.30	230.80	18.82	8.39	8.22	8.06	12.18	1.04	1.53	1.04	1.52
Min	27.27	27.84	28.52	19.49	16.88	228.10	224.80	218.80	17.02	7.72	7.95	7.99	11.51	0.96	1.41	0.98	1.44
Mean	29.01	28.80	29.04	19.60	17.28	232.95	229.80	224.73	17.80	8.05	8.09	8.02	11.89	1.00	1.47	1.01	1.48
***Rusa unicolor*** (N=4)																	
Max	40.63	36.93	42.15	29.40	24.01	258.20	254.60	246.60	28.47	6.52	6.99	6.19	8.83	0.96	1.38	1.11	1.44
Min	34.82	35.34	36.63	25.66	23.56	226.70	223.50	220.10	24.56	6.32	6.34	6.08	8.64	0.95	1.25	0.97	1.40
Mean	37.68	36.16	39.38	27.66	23.69	241.93	238.58	233.05	26.40	6.43	6.69	6.15	8.75	0.96	1.31	1.04	1.42
***Bos sauveli*** (N=2)																	
Max	53.65	51.21	52.82	33.45	31.96	282.60	273.50	263.70	32.34	5.28	5.53	5.36	8.47	1.02	1.53	1.05	1.58
Min	53.44	51.07	52.67	33.36	31.85	282.40	273.10	263.40	32.20	5.27	5.51	5.35	8.44	1.01	1.53	1.04	1.57
Mean	53.55	51.14	52.75	33.41	31.91	282.50	273.30	263.55	32.27	5.28	5.52	5.36	8.46	1.02	1.53	1.05	1.58
***Bos javanicus*** (N=6)																	
Max	54.98	51.89	55.74	37.28	35.52	257.80	246.40	246.90	32.40	5.14	5.56	5.12	7.58	1.00	1.49	1.08	1.55
Min	48.30	46.33	50.31	33.94	30.30	243.00	234.00	230.80	29.05	4.59	4.78	4.53	6.77	0.90	1.35	0.95	1.46
Mean	51.13	49.68	53.05	35.21	33.34	250.98	238.20	236.65	30.41	4.92	5.07	4.74	7.14	0.96	1.41	1.03	1.51
***Bos gaurus*** (N=6)																	
Max	60.25	59.22	64.81	41.66	42.06	273.10	265.70	261.20	36.04	4.85	5.33	4.34	7.18	0.94	1.50	1.14	1.66
Min	52.35	50.30	61.89	37.36	35.48	254.00	245.70	239.10	32.84	4.43	4.61	3.92	6.10	0.81	1.21	1.02	1.56
Mean	57.31	53.27	63.77	39.75	38.00	265.57	257.70	251.92	34.65	4.64	5.00	4.17	6.70	0.90	1.34	1.08	1.61
***Bubalus arnee*** (N=6)																	
Max	57.42	49.56	66.93	37.74	37.03	226.40	213.50	206.80	33.13	4.05	4.67	3.41	6.47	0.86	1.41	1.20	1.91
Min	52.56	46.69	62.34	34.87	32.31	212.80	198.20	200.80	30.30	3.91	4.54	3.34	5.92	0.84	1.27	1.13	1.76
Mean	55.45	48.13	65.28	36.01	34.84	221.17	208.05	203.58	31.39	3.99	4.59	3.39	6.15	0.85	1.34	1.15	1.81

**Appendix 13.** Measurements (in millimeters) of mandibles of large bovids from Khok Sung. Numbers within the parentheses refer to the numbers used in von den Driesch’s metrical methods (1976: fig. 21). * indicates measurements of the maximum length of the preservation according to incomplete specimens. “es” refers to an estimated value of the full length due to incomplete specimens.

**Taxon**	***Bos sauveli***		***Bos gaurus***		***Bubalus arnee***				
**Mandible no.**	DMR-KS-05-03-9-1	DMR-KS-05-04-9-1	DMR-KS-05-03-00-1	DMR-KS-05-04-3-1	DMR-KS-05-03-20-10	DMR-KS-05-03-20-20	DMR-KS-05-03-20-2	DMR-KS-05-03-10-3	DMR-KS-05-03-20-1
**Metrical parameters (mm)**					Juvenile	Juvenile	Juvenile		
(1) Length from the angle: Gonion caudale–Infradentale	450*	446.21	–	–	377*	–	–	461.14	464.54
(2) Length from the condyle: aboral border of the condyle process–Infradentale	483.55	478.08	–	–	371.28*	–	–	490.82	493.34
(3) Length: Gonion caudale–aboral border of the alveolus of m3	132.51	122.31	–	137.11	83.57	–	94.92	126.23	127.48
(4) Length of the horizontal ramus: aboral border of the alveolus of m3–Infradentale	313.60	312.1	–	–	278.91*	–	–	324.52	325.67
(5) Length: Gonion caudale–oral border of the alveolus of p2	307.52	289.11	–	305.44	245.18	–	–	301.04	299.77
(6) Length: Gonion caudale–the most aboral indentation of the mental foramen	397.32	356.52	–	–	311.78	–	–	375.44	374.89
(7) Length of the cheek tooth row, measured along the alveoli on the buccal side	169.58	165.66	–	171.58	–	–	–	173.72	174.52
(8) Length of the molar row, measured along the alveoli on the buccal side	107.01	103.86	101.01	109.10	–	–	–	111.57	113.69
(9) Length of the premolar row, measured along the alveoli on the buccal side	65.31	56.54	–	60.26	78.22 (p2–dp4)	–	79.09 (p2–dp4)	60.31	59.02
(11) Length of the diastema: oral border of the alveolus of p2–aboral border of the alveolus of i4	110.41	116.57	–	–	106.53	–	–	116.41	118.35
(12) Aboral height of the vertical ramus: Gonion ventrale–highest point of the condyle process	189.21	175.03	–	186.53	139.48	–	143.18	181.59	183.21
(13) Middle height of the vertical ramus: Gonion ventrale–deepest point of the mandibular notch	168.52	165.07	–	181.08	139.97	–	145.30	177.52	169.18
(14) Oral height of the vertical ramus: Gonion ventrale–Coronion	254.16	249.58	–	251.12	205.47	–	–	236.58	246.55
(15a) Height of the mandible behind m3 from the most aboral point of the alveolus on the buccal side	90.59	90.40	–	89.86	–	–	80.84	94.89	96.63
(15b) Height of the mandible in front of m1	64.88	68.83	70.33	65.79	66.84	64.28	68.61	68.48	69.63
(15c) Height of the mandible in front of p2	54.99	55.04	–	57.77 (es)	41.63	–	42.38	55.57	56.33

**Appendix 14.** Measurements (in millimeters) of crania of *Bubalus arnee* from Khok Sung. Numbers within the parentheses refer to the numbers used in von den Driesch’s metrical methods (1976: fig. 8). * indicates measurements of the maximum length of the preservation according to incomplete specimens. “es” refers to an estimated value of the full length due to incomplete specimens.

Taxon	*Bubalus arnee*			
Cranium no.	DMR-KS-05-03-16-3	DMR-KS-05-03-21-1	DMR-KS-05-03-11-1	DMR-KS-05-03-20-1
**Metrical parameters (mm)**				
(1) Total length: Akrokranion–Prosthion	–	–	–	568.97
(2) Condylobasal length: aboral border of the occipital condyles–Prosthion	–	–	–	565.98*
(3) Basal length: Basion–Prosthion	–	–	–	553.31
(4) Short skull length: Basion–Premolare	–	426.18	–	381.21
(5) Premolar–Prosthion	–	–	–	185.07
(6) Neurocranium length: Basion–Nasion	–	230 (es)	–	246.53
(7) Viscerocranium length: Nasion–Prosthion	–	–	–	344.25
(8) Median frontal length: Akrokranion–Nasion	–	–	–	206.61
(9) Greatest frontal length: Akrokranion–the median point of intersection of the line joining the oral points of the frontals	–	–	–	248.82
(10) Short upper cranium length: Akrokranion–Rhinion	–	–	–	440.31
(11) Akrokranion–Infraorbitale of one side	–	–	–	385.55
(12) Greatest length of the nasals: Nasion–Rhinion	–	–	–	230.87
(13) From the aboral border of one occipital condyle to the Entorbitale of the same side	–	263.18	–	243.75
(14) Lateral facial length: Entorbitale–Prosthion	–	–	–	389.11
(15) From the aboral border of one occipital condyle to the Infraorbitale of the same side	–	–	–	390.16
(16) Infraorbitale–Prosthion	168.66	–	–	181.36
(17) Dental length: Postdentale–Prosthion	–	–	–	341.43
(18) Oral palatal length: Palatinoorale–Prosthion	–	–	–	272.03
(19) Lateral length of the premaxilla: Nasointermaxillare–Prosthion	–	–	–	197.68
(20) Length of the cheek tooth row (measured along the alveoli)	–	–	169.58 (right)	165.56 (left)
(21) Length of the molar row (measured along the alveoli on the buccal side)	–	110 (es) (right)	98.06 (right)	102.63 (left)
(22) Length of the premolar row (measured along the alveoli on the buccal side)	62.56 (right), 62.09 (left)	–	70.34 (right)	65.76 (right), 68.70 (left)
(23) Greatest inner length of the orbit: Ectorbitale–Entorbitale	–	–	–	67.73 (right), 68.26 (left)
(24) Greatest inner height of the orbit	–	–	–	55.51 (right), 61.44 (left)
(25) Greatest mastoid breadth: Otion–Otion	–	188.71	–	–
(26) Greatest breadth of the occipital condyles	–	106.59	–	–
(27) Greatest breadth at the bases of the paraoccipital processes	–	154.23	–	–
(28) Greatest breadth of the foramen magnum	–	47.38	–	–
(29) Height of the foramen magnum: Basion–Opisthion	–	40.96	–	–
(30) Least occipital breasdth: the distance between the most medial points of the aboral borders of the temporal grooves	–	117.35	–	93.06
(31) Least breadth between the bases of the horn cores	–	129.32	–	151.45
(32) Least frontal breadth: breadth of the narrowest part of the frontal aboral of the orbits	–	210 (es)	–	222.23
(33) Greatest breadth across the orbits = Greatest frontal breadth = greatest breadth of skull: Ectorbitale–Ectorbitale	–	–	–	231.78
(34) Least breadth between the orbits: Entorbitale–Entorbitale	–	–	–	165.52
(35) Facial breadth: across the facial tuberosities	–	–	–	167.42
(36) Greatest breadth across the nasals	–	–	–	64.01
(37) Breadth across the premaxillae on the oral protuberances	–	–	–	112.89
(38) Greatest palatal breadth: measured across the outer borders of the alveoli	–	–	–	148.14
(39) Least inner height of the temporal groove, roughly from the middle of one bone edge to the other	–	40.08	–	24.57
(40) Greatest height of the occipital region: Basion–highest point of the intercornual ridge in the median plane	–	139.25	–	–
(41) Least height of the occipital region: Opisthion–highest point of the intercornual ridge in the median plane	–	115.68	–	138.74
(42) Distance between the horn core tips	–	540 (es)	–	607.18
(43) Greatest tangential distance between the outer curves of the horn cores	–	–	–	341.38 (right), 333.17 (left)
(44) Horn core basal circumference	–	199.91 (left)	–	341.38 (right)
(45) Greatest (oro–aboral) diameter of the horn core base	–	72.03 (right), 70.77 (left)	–	132.71 (right), 131.53 (left)
(46) Least (dorso–basal) diameter of the horn core base	–	44.67 (right), 49.49 (left)	–	56.05 (right), 53.45 (left)
(47) Length of the outer curvature of the horn core	–	102.21 (right), 258.17 (left)	–	164* (right), 215* (left)

**Appendix 15.** Fauna lists of large mammalian species from the Middle Pleistocene of Central eastern and South China. Locality abbreviations: YCK, Yenchingkou; KLS ; Koloshan; WM, Wuming; DX, Daxin; HJ, Hejiang; GX, Ganxian; PXDD, Panxian Dadong; WY, Wuyun; MB, Maba; HST, Hoshantung; HG, Hsingan. The fauna lists and ages follow Colbert and Hooijer (1953) for YCK; Kahlke (1961) for KLS, HST, and HG; Rink et al. (2008) for WM and DX; Zhang et al. (2014) for HJ; Wang et al. (2014) for GX; Han and Xu (1985), Bekken et al. (2004), and Schepartz et al. (2005) for PXDD; Chen et al. (2002), Rink et al. (2008), and Wang et al. (2014) for WY; Han and Xu (1985), Wu et al. (2011), and Shen et al. (2014) for MB. The subspecies-level identifications are not taken into account.

Region	Central eastern China		South China								
**Locality**	**YCK**	**KLS**	**WM**	**DX**	**HJ**	**GX**	**PXDD**	**WY**	**MB**	**HST**	**HG**
**Approximate age (ka)**			745 to 481	380 to 308	400 to 320	> 350	300 to 130	279 to 76	> 230		
**Taxa**											
**PRIMATES**											
*Macaca* sp.			+	+	+			+	+	+	
*Macaca assamensis*							cf.				
*Macaca robustus*		+									
*Macaca arctoides*							+				
Colobinae indet.							+				
*Presbytis* sp.								+			
*Rhinopithecus* sp.				+							
*Rhinopithecus roxellana*	+								+		
*Trachypithecus* sp.					+						
*Nomascus* sp.					+						
*Hylobates* sp.				+							
*Bunopithecus sericus*	+										
*Szechuanopithecus yangtsensis*		+									
*Pongo* sp.				+			+		+		
*Pongo pygmaeus*						+		+		+	+
*Gigantopithecus blacki*			+	+	+						
*Homo* sp.							+		+		
**CARNIVORA**											
Canidae indet.		+								+	
*Canis lupus*							+				
*Cuon simplicidens*		+									
*Cuon alpinus* (=*javanicus*)	+			+				cf.	+		
*Vulpes vulpes*							+				
*Ursus* sp.			+		+						
*Ursus angustidens*										+	
*Ursus thibetanus*	+	+		+		+	+	+	+		+
*Helarctos malayanus*											
*Ailuropoda melanoleuca*	+	+	+	+	+	+	+		+	+	+
*Ailuropoda microta*								+			
*Ailurus fulgens*										+	
*Mustela sibirica*		+					+				
*Martes flavigula*	+										
*Martes sinensis*		+									
*Meles* sp.					+						
*Melogale* sp.					+						
*Parameles simplicidens*		+									
*Arctonyx* sp.										+	
*Arctonyx collaris*	+			+		+		+	+		+
*Viverra* sp.							+				
*Viverra zibetha*	+								+		
*Viverricula malaccensis*				+							
*Lutra* sp.			+								
*Felis* sp.	+						+		+		+
*Felis teilhardi*								+			
*Lynx lynx*							+			+	
*Panthera pardus*					+			+		+	
*Panthera tigris*	+	+			+		+	+	cf.	+	+
*Paradoxurus* sp.								+			
*Prionailurus bengalensis*							+				
*Paguma larvata*				+					+		
*Hyaena* sp.			+		+		+				
*Crocuta crocuta*	+								+	+	+
											
**PROBOSCIDEA**											
*Stegodon* sp.			+							+	
*Stegodon orientalis*	+			+	+	+	+	+	+		+
*Elephas maximus*						+		+			
*Palaeoloxodon* sp.											+
*Palaeoloxodon namadicus*	+								+	+	
**PERISSODACTYLA**											
*Tapirus* sp.									+		
*Tapirus indicus*											+
*Tapirus sinensis*					+			+			
*Megatapirus augustus*	+	+		+			+	+	+	+	
Rhinocerotidae indet.											
*Rhinoceros* sp.										+	
*Rhinoceros unicornis*	?										
*Rhinoceros sinensis*	+	+	+	+	+		+		+		+
*Rhinoceros fusuiensis*						+		+			
**ARTIODACTYLA**											
*Sus* sp.		+	+	+			+		+	+	+
*Sus scrofa*	+		+		+	+		+	+		
*Sus xiaozhu*					+	+					
*Sus bijiashanensis*			+	+							
*Dicoryphochoerus ultimus*				+							
Cervidae indet.		+					+			+	+
*Cervus* sp. or *Rusa* sp.				+		+		+			
*Cervus yunnanensis*						+					
*Rusa unicolor*	+				cf.		+		+		
*Elaphodus cephalophus*	+										
*Muntiacus* sp.				+	+	+	+	+		+	+
*Muntiacus muntjak*	+								+		
*Muntiacus szechuanensis*		+									
*Hydropotes* sp.									+		
*Mochus* sp.							+				
*Mochus moschiferus*	+										
Bovidae indet.			+			+		+		+	+
*Bos* sp. or *Bubalus* sp.				+	+		+		+		
*Bos gaurus*	+										
*Bubalus arnee*	+										
*Bubalus brevicornis*		+									
Caprinae indet.		+		+						+	
*Capricornis sumatraensis*	+		cf.				+	+			
*Naemorhedus goral*	+						+				
*Megalovis guangxiensis*				+	+		+				

**Appendix 16.** Fauna lists of large mammalian species from the Middle Pleistocene of Southeast Asia. Locality abbreviations: KS, Khok Sung; TWN ; Thum Wiman Nakin; TPKP, Thum Phra Khai Phet; KPN, Kao Pah Nam; TPD, Thum Phedan; TK, Tham Khuyen; TH, Tham Hai; TO, Tham Om; MG, Mogok Caves; PNL, Phnom Loang; BDB, Boh Dambang; BDC, Badak Cave; TB, Tambun (Kinta Valley); KDBB, Kedung Brubus; TNHK, Trinil Hauptknochenschicht; ND, Ngandong. The fauna lists and ages follow Tougard (2001) and Esposito et al. (1998, 2002) for TWN; Tougard (1998) and Filoux et al. (2015) for TPKP; Pope et al. (1981) for KPN; Yamee and Chaimanee (2005) for TPD; Olsen and Ciochon (1990), Ciochon et al. (1996), and Ciochon (2009) for TK, TH, and TO; Colbert (1938, 1943) and Takai et al. (2006) for MG; Beden and Guérin (1973) for PNL; Demeter et al. (2013) for BDB; Ibrahim et al. (2013) for BDC; Hooijer (1962) and Medway (1972) for TB; van den Bergh et al. (2001) for KDBB; van den Bergh et al. (2001) and Joordens et al. (2015) for TNHK; and Santa Luca (1980), van den Bergh et al. (2001), and Indriati et al. (2011) for ND. The subspecies-level identifications are not taken into account.

Country	Thailand					Vietnam			Myanmar	Cambodia		Malaysia		Indonesia		
Locality	KS	TWN	TPKP	KPN	TPD	TK	TH	TO	MG	PNL	BDB	BDC	TB	KDBB	TNHK	ND
**Approximate age (ka)**	**213 to 120**	**> 169 ± 11**	≈**TWN**	≈**690**		**475 ± 125**	≈**TK**	**250 to 140**		**> TWN**	≈**PNL**	**274 to 208**		**800 to 700**	**540 to 430**	**> 143**
**Taxa**																
**PRIMATES**																
*Macaca* sp.	+		+			+	+				+					
*Macaca assamensis*						cf.										
*Macaca nemestrina*		cf.										+				
*Macaca fascicularis*															+	+
*Trachypithecus* sp.		+														
*Trachypithecus cristatus*															+	
Hominoidea indet.						+	+									
*Nomascus concolor*						cf.										
*Hylobates* sp.								+								
*Pongo* sp.			+	?								+				
*Pongo pygmaeus*		+				+	+			+	+					
*Gigantopithecus blacki*						+	?									
*Homo* sp.		+						+								
*Homo erectus*														+	+	+
**CARNIVORA**																
*Cuon* sp.	+					+		+								
*Cuon alpines* (=*javanicus*)											+				+	
*Ursus thibetanus*		+	+			+	+	+			+	+				
*Helarctos malayanus*						+						+				
*Ailuropoda melanoleuca*		+	+			+		+	+							
*Martes* sp.										+						
*Martes flavigula*		+														
*Arctonyx collaris*		+				+		+								
*Viverra zibetha*								cf.								
*Lutrogale palaeoleptonyx*														+		
*Cynogale* sp.													+			
*Felis* sp.								+								
*Panthera pardus*			cf.				+									
*Panthera tigris*				+			+	+		+				+	+	+
*Paradoxurus hermaphroditus*		+				cf.										
*Prionailurus bengalensis*											+				+	
*Paguma larvata*		+						cf.								
*Hyaena brevirostris*														+		
*Crocuta* sp.				+												
*Crocuta crocuta*	+	+	+		+					+	+					
**PHOLIDOTA**																
*Manis palaeojavanica*														+		
**PROBOSCIDEA**																
*Stegodon orientalis*	cf.					+	+	+	+							
*Stegodon trigonocephalus*														+	+	+
*Elaphas* sp.	+								+							
*Elephas maximus*		cf.														
*Elephas celebensis*														?		
*Elephas hysudrindicus*														+		+
*Palaeoloxodon namadicus*						+		cf.	?				+			
**PERISSODACTYLA**																
*Tapirus* sp.						+	+	+								
*Tapirus indicus*		+								+				+		+
*Megatapirus augustus*						+		+								
Rhinocerotidae indet.												+				
*Rhinoceros* sp.					+			+	+			+				
*Rhinoceros sondaicus*	+	+	+							+			+	+	+	
*Rhinoceros unicornis*	+	+												+		
*Rhinoceros sinensis*						+	+	+								
**ARTIODACTYLA**																
*Sus* sp.						+		+				+	+			
*Sus barbatus*	+	cf.	cf.													
*Sus scrofa*		+	+		+	+	+	+	+		+	+				
*Sus lydekkeri*						cf.										
*Sus brachygnathus*															+	?
*Sus macrognathus*														+		+
Cervidae indet.		+	+		+											+
*Cervus* sp. or *Rusa* sp.				+		+		+	+				+	+		+
*Panolia eldii*	+	+		+												
*Rusa unicolor*	+	+	+			+	cf.	+		+	+	+				
*Rusa leptodus*										cf.						
*Elaphodus* sp.								+								
*Axis porcinus*		?	?								?					
*Axis axis*	+															
*Axis lydekkeri*														+	+	
*Muntiacus* sp.			+					+		+						
*Muntiacus muntjak*		+	+			+		+			+	+		+	+	
Bovidae indet.												+				
*Bos* sp. or *Bubalus* sp.			+		+				+		+		+			
*Bos sauveli*	+	+	+													
*Bos javanicus*		+	+													
*Bos gaurus*	+	+		cf.		+		+								
*Bibos palaeosondaicus*														+	+	+
*Bubalus arnee*	+	+				+		+		cf.	+					
*Bubalus palaeokerabau*														+	+	+
*Duboisia santeng*													+	+	+	
*Epileptobos groeneveldtii*														+		
Caprinae indet.										+						
*Capricornis sumatraensis*	+	+	+		+			+			+	+				
*Naemorhedus* sp.			+													
*Hippopotamus* sp.				+												
*Hexaprotodon* sp.													+			
*Hexaprotodon sivalensis*														+		+

**Appendix 17.** Fauna lists of large mammalian species from the Late Pleistocene of Southeast Asia and South China. Locality abbreviations: CMBFS, the Cave of the Monk (Ban Fa Suai); LPC, Lower Pubu Cave; LN, Luna Cave; NL, Nam Lot; THS, Tam Hang South; HH, Hang Hum; LT, Lang Trang; DUO, Duoi U’Oi; MUO, Ma U’Oi; KL, Keo Leng; GGR, Gua Gunung Runtuh; GC, Gua Cha; BTC, Batu Caves; **N**, Niah Caves; PN, Punung; GD, Gunung Dawung; WJ, Wajak; LA, Lida Ajer; SBB, Sibrambang. The fauna lists and ages follow Zeitoun et al. (2005, 2010) for CMBFS; Wang et al. (2007, 2014) for LPC and LN; Bacon et al. (2008a, 2011, 2012, 2015) for NL and THS; Kha (1976), Cuong (1985), and Olsen and Ciochon (1990) for HH and KL; de Vos and Long (1993) and Long et al. (1996) for LT; Bacon et al. (2008b) for DUO; Bacon et al. (2004, 2006) for MUO; Davidson (1994) and Bulbeck (2014) for GGR; Groves (1985) and Bulbeck (2003) for GC; Ibrahim et al. (2013) for BTC; Medway (1972), Harrison (1996), and Barker et al. (2007) for N; Badoux (1959), Storm and de Vos (2006), and Westaway et al. (2007) for PN; Storm et al. (2005) for GD; van den Brink (1982) and Storm et al. (2013) for WJ; and de Vos (1983) for LA and SBB. The subspecies-level identifications are not taken into account.

Country	Thailand	South		Laos		Vietnam					Malaysia				Indonesia				
															Java			Sumatra	
Locality	CMBFS	LPC	LN	NL	THS	HH	LT	DUO	MUO	KL	BTC	GGR	GC	N	PN	GD	WJ	LA	SBB
**Approximate age (ka)**	**> 32–19**		≈**70**	**86 to 72**	**94 to 60**	**140 to 80**	**100 to 80**	**66 ± 3**	**> 49**	**30 to 20**	**66 to 33**	**13.6 to 2.6**	**6.3 to 0.8**	**< 45**	**128 to 118**	≈**PN**	**37 to 29**	≈**PN**	≈**PN**
**Taxa**																			
**PRIMATES**																			
*Nycticebus coucang*														+					
*Macaca* sp.	+	+	+	+	+	+	+	+	+	+					+			+	+
*Macaca assamensis*	cf.									cf.									
*Macaca nemestrina*	cf.										+		+	+					
*Macaca anderssoni*	cf.																		
*Macaca mulatta*										cf.									
*Macaca fascicularis*											+		+	+					
Colobinae indet.								+											
*Presbytis* sp.				?	?													+	+
*Presbytis melalophos*													+						
*Presbytis rubicunda*														+					
*Trachypithecus* sp.				?	?		+						+			+			
*Trachypithecus cristatus*														+			+	+	+
*Pygatrix nemaeus*	cf.																		
*Hylobates* sp.	+		+		+	+	+	+		+									+
*Hylobates lar*													+						
*Hylobates muelleri*														+					
*Hylobates moloch*															cf.				
*Symphalangus syndactylus*															+	+		+	
*Semnopithecus* sp.										+									
*Pongo* sp.											+								
*Pongo pygmaeus*	cf.			+	+	+	+	+		+				+	+	+		+	+
*Homo* sp.			+	+		+		+	+										
*Homo sapiens*										+		+			+		+	+	
**CARNIVORA**																			
*Nyctereutes* sp.						+													
*Cuon* sp.	+					+			+										+
*Cuon alpinus*	cf.			+	+	+	+	+				+							
Canidae indet.	+										+								
Ursidae indet.	+																		
*Ursus thibetanus*	cf.	+		+	+	+	+	+		+	+								
*Helarctos malayanus*	cf.				+		+	+			+	+	+	+	+	+		+	+
*Ailuropoda melanoleuca*	+	+	+	+			+			+									
*Martes flavigula*				+	? or cf.														
*Arctonyx* sp.																			+
*Arctonyx collaris*	+	+			+	+	+	+		+									
*Meles meles*				+	+														
*Mustela nupides*														+					
*Melogale personata*					+														
*Melogale everetti*														+					
Viverridae indet.								+											
*Viverra zibetha*				+	+			+		cf.									
*Viverra megaspila*								cf.											
*Viverra tangalunga*											+			+					
*Lutra perspicillata*							+												
*Lutrogale sumatrana*														+					
*Herpestes* sp.														+					
*Catopuma temminckii*				cf.			+												+
*Neofelis nebulosa*						+		+						+					+
*Panthera pardus*						+	+	+											+
*Panthera tigris*	cf.				+	+	+	+			+	?		+	+		+		+
*Paradoxurus* sp.						+													
*Paradoxurus hermaphroditus*					+	cf.	+			cf.									
*Prionailurus bengalensis*					cf.														
*Paguma* sp.																			+
*Paguma larvata*						+													
*Arctictis binturong*													+	+					
*Hemigalus derbyanus*														+					
Hyaenidae indet.	+																		
*Crocuta crocuta*				+															
**PHOLIDOTA**																			
*Manis javanica*														+					
*Manis palaeojavanica*														+					
																			
**PROBOSCIDEA**																			
*Stegodon* sp.	+																		
*Stegodon orientalis*			+	cf.	+	+	+			+									
*Elaphas* sp.	+			+	+			+	+							+			
*Elephas maximus*		+												+	+			+	+
*Palaeoloxodon namadicus*						+	+		cf.										
**PERISSODACTYLA**																			
*Tapirus* sp.	+			+															
*Tapirus indicus*					+	+	+	+			+	+		+	+	+	+	+	+
*Tapirus sinensis*		+																	
*Megatapirus augustus*					+	+				+									
Rhinocerotidae indet.	+			+	+			+			+								
*Rhinoceros* sp.				+	+			+											
*Rhinoceros sondaicus*	cf.			+	+			+	cf.					+	+	+	+		+
*Rhinoceros unicornis*	cf.			+	+	+		+	cf.	+									
*Rhinoceros sinensis*	cf.	+																	
*Dicerorhinus sumatrensis*	cf.						+	+			+			+	+			+	+
*Equus hemionus*		+																	
**ARTIODACTYLA**																			
*Sus* sp.	+									+	+					+			
*Sus barbatus*	cf.				cf.			+			+		+	+	+			+	+
*Sus scrofa*	cf.	+	+	+	+	+	+	+	+	+	+	+	+		+		+	+	+
*Sus officinalis*						cf.													
*Sus xiaozhu*		+																	
*Sus lydekkeri*						cf.				cf.									
Cervidae indet.	+					+				+									
*Cervus* sp. or *Rusa* sp.	+	+	+												+	?		+	+
*Panolia eldii*	cf.																		
*Rusa unicolor*	cf.			+	+	+	+	+	cf.	+	+	+	+	+					
*Rusa timorensis*																	+		
*Cervus nippon*	cf.																		
*Axis porcinus*	cf.																		
*Muntiacus* sp.	+	+	+							+									
*Muntiacus muntjak*	cf.			+	+		+	+	+	+	+	+	+	+	+	+	+	+	+
*Muntiacus vuquangensis*	cf.																		
*Tragulus javanicus*														+					
*Tragulus napu*												+		+					
Bovidae indet.	+	+	+	+							+					+			
*Bos* sp.	+			+									+		+				
*Bos sauveli*	cf.				cf.														
*Bos javanicus*	cf.													+				+	+
*Bos gaurus*	cf.					+						+							
*Bubalus* sp.						+													
*Bubalus arnee*	cf.			+	+		+	cf.		+					+				+
*Pseudoryx* sp.	+																		
Caprinae indet.						+													
*Capricornis sumatraensis*	cf.			+	+		+	+		+	+		+		+			+	+
*Naemorhedus* sp.	cf.																		
*Naemorhedus caudatus*	cf.																		
*Naemorhedus goral*	cf.																		

**Appendix 18.** Fauna lists of extant large mammalian species from Southeast Asia and South China. The biogeographic affinities of the mammalian species are given and abbreviated as (I) for Indochinese taxa, (S) for Sundaic taxa, (O) for other geographic taxa (e.g., Palearctic and Indian regions), and (W) for widespread taxa. Data are compiled from Lekagul and McNeely (1988), Corbet and Hill (1992), and Nowak (1999). The binomial nomenclature is modified from categories of the IUCN red list of threatened species (2015) and from Groves and Grubb (2011).

Biogeographic province	Indochinese province					Thailand (Kra Isthmus)		Sundaic province			
Country	South China	Myanmar	Laos	Cambodia	Vietnam	North	South	Malaysia	Sumatra	Java	Borneo
**Taxa**											
**PRIMATES**											
*Nycticebus coucang* (I, S)	+	+	+	+	+	+	+	+	+	+	+
*Nycticebus pygmaeus* (I)	+			+	+	+					
*Tarsius bancanus* (S)									+		+
*Macaca arctoides* (I)	+	+	+	+	+	+	+				
*Macaca assamensis* (I, O)	+	+	+		+	+					
*Macaca fascicularis* (S)		+	+	+	+	+	+	+	+	+	+
*Macaca mulatta* (I, O)	+	+	+		+		+				
*Macaca nemestrina* (I, S)		+	+	+	+	+	+	+	+		+
*Macaca thibetana* (I)	+										
*Presbytis comata* (S)										+	
*Presbytis femoralis* (S)		+				+	+	+	+		
*Presbytis frontana* (S)											+
*Presbytis hosei* (S)											+
*Presbytis melalophos* (S)									+		
*Presbytis potenziani* (S)									+		
*Presbytis rubicunda* (S)											+
*Presbytis thomasi* (S)									+		
*Trachypithecus auratus* (S)										+	
*Trachypithecus cristatus* (S)		+	+	+	+	+	+	+	+		+
*Trachypithecus francoisi* (I)	+		+		+						
*Trachypithecus obscurus* (S)		+				+	+	+			
*Trachypithecus phayrei* (I)	+	+	+		+	+					
*Trachypithecus pileatus* (I)		+									
*Pygathrix nemaeus* (I)			+	+	+						
*Rhinopithecus avunculus* (I)					+						
*Rhinopithecus bieti* (I)	+										
*Rhinopithecus brelichi* (I)	+										
*Nasalis larvatus* (S)											+
*Simias concolor* (S)									+		
*Nomascus concolor* (I)	+		+		+						
*Nomascus gabriellae* (I)				+	+						
*Nomascus leucogenys* (I)	+		+		+						
*Hoolock hoolock* (I)	+	+									
*Hylobates agilis* (S)							+		+	+	+
*Hylobates klossii* (S)										+	
*Hylobates lar* (I, S)	+	+				+	+	+	+		
*Hylobates moloch* (S)										+	
*Hylobates muelleri* (S)											+
*Hylobates pileatus* (I)			+	+		+					
*Symphalangus syndactylus* (S)								+	+		
*Pongo pygmaeus* (S)									+		+
**CARNIVORA**											
*Canis aureus* (I, O)		+	+								
*Canis lupus* (W)	+										
*Vulpes vulpes* (I, O)	+				+						
*Nyctereutes procyonoides* (I, O)	+				+						
*Cuon alpinus* (W)	+	+	+	+	+	+	+	+	+	+	
*Ursus thibetanus* (I)	+	+	+		+	+					
*Helarctos malayanus* (I, S)	+	+	+	+	+	+	+	+	+		+
*Ailuropoda melanoleuca*	+										
*Ailurus fulgens* (I)	+	+									
*Mustela kathiah* (I, O)	+	+	+		+						
*Mustela lutreolina* (S)									+	+	
*Mustela nivalis* (I, O)	+				+						
*Mustela nudipes* (S)							+	+	+		+
*Mustela sibirica* (I, O)	+	+	+		+	+					
*Mustela strigidorsa* (I)	+	+	+		+	+					
*Martes flavigula* (I, S, O)	+	+	+	+	+	+	+	+	+	+	+
*Meles meles* (I, O)	+		+		+						
*Arctonyx collaris* (I, O)	+	+	+	+	+	+	+		+		
*Melogale everetti* (S)											+
*Melogale moschata* (I)	+	+			+						
*Melogale orientalis* (S)										+	
*Melogale personata* (I)		+			+	+					
*Mydaus javanensis* (S)									+	+	+
*Lutra lutra* (W)	+	+	+	+	+	+			+		
*Lutra sumatrana* (S)		+		+	+		+	+	+		+
*Lutrogale perspicillata* (W)	+	+	+	+	+	+	+	+	+	+	+
*Amblonyx cinereus* (W)	+	+	+	+	+	+	+	+	+	+	+
*Viverra megaspila* (I)	+	+	+	+	+	+	+	+			
*Viverra tangalunga* (S)								+	+		+
*Viverra zibetha* (I)	+	+	+	+	+	+	+	+			
*Viverricula indica* (W)	+	+	+	+	+	+	+	+	+	+	
*Prionodon linsang* (S)		+				+	+	+	+	+	+
*Prionodon pardicolor* (I)	+	+	+		+	+					
*Paradoxurus hermaphroditus* (W)	+	+	+	+	+	+	+	+	+	+	+
*Paguma larvata* (I, S, O)	+	+	+	+	+	+	+	+	+		+
*Arctictis binturong* (I, S)	+	+	+	+	+	+	+	+	+	+	+
*Arctogalidia trivirgata* (I, S)	+	+	+	+	+	+	+	+	+	+	+
*Hemigalus derbyanus* (S)		+					+	+	+		+
*Chrotogale owstoni* (I)	+		+		+						
*Diplogale hosei* (S)											+
*Cynogale bennettii* (S)								+	+		+
*Herpestes brachyurus* (S)								+	+		+
*Herpestes javanicus* (I, S, O)	+	+	+	+	+	+	+	+		+	
*Herpestes semitorquatus* (S)									+		+
*Herpestes urva* (I)	+	+	+	+	+	+	+	+			
*Felis chaus* (W)	+	+	+	+	+	+					
*Prionailurus bengalensis* (W)	+	+	+	+	+	+	+	+	+	+	+
*Prionailurus planiceps* (S)							+	+	+		+
*Prionailurus viverrinus* (W)	+	+	+	+	+	+			+	+	
*Catopuma badia* (S)											+
*Catopuma temminckii* (I, S)	+	+	+	+	+	+	+	+	+		
*Pardofelis marmorata* (I, S)	+	+			+		+	+	+		+
*Neofelis nebulosa* (I, S)	+	+	+	+	+	+	+	+	+		+
*Panthera pardus* (W)	+	+	+	+	+	+	+	+		+	
*Panthera tigris* (W)	+	+	+	+	+	+	+	+	+	+	
**PHOLIDOTA**											
*Manis javanica* (S)		+					+	+	+	+	+
*Manis pentadactyla* (I)	+	+	+		+	+					
**PROBOSCIDEA**											
*Elephas maximus* (I, O)		+	+	+	+	+	+	+	+		+
**PERISSODACTYLA**											
*Tapirus indicus* (S)		+	+			+	+	+	+		
*Rhinoceros sondaicus* (I, S)	+	+		+	+	+	+	+	+	+	
*Dicerorhinus sumatrensis* (I, S)	+	+	+	+	+	+	+	+	+		+
**ARTIODACTYLA**											
*Sus barbatus* (S)								+	+		+
*Sus scrofa* (W)	+	+	+	+	+	+	+	+	+	+	
*Sus verrucosus* (S)										+	
*Tragulus javanicus* (S)		+	+	+	+	+	+	+	+	+	+
*Tragulus napu* (S)		+	+		+	+	+	+	+		+
*Moschus berezovskii* (I)	+				+						
*Panolia eldii* (I)		+	+	+	+						
*Cervus nippon* (I)	+										
*Rusa unicolor* (W)	+	+	+	+	+	+	+	+	+		+
*Rusa timorensis* (S, O)										+	
*Axis porcinus* (I, O)	+	+	+	+	+	+					
*Axis kuhlii* (S)										+	
*Muntiacus atherodes* (S)											+
*Muntiacus feae* (I)		+				+					
*Muntiacus gongshanensis* (I)	+	+									
*Muntiacus muntjak* (W)	+	+	+	+	+	+	+	+	+	+	+
*Muntiacus reevesi* (I)	+					+					
*Muntiacus rooseveltorum* (I)			+								
*Elaphodus cephalophus* (I)	+	+									
*Hydropotes inermis* (I)	+										
*Bos sauveli* (I)			+	+	+	+					
*Bos javanicus* (I, S)		+	+	+	+	+	+			+	+
*Bos gaurus* (I)	+	+	+	+	+	+	+	+			
*Bubalus arnee* (I, O)				+	+	+					
*Budorcas taxicolor* (I)	+	+									
*Capricornis sumatraensis* (I, S, O)	+	+	+	+	+	+	+	+	+		
*Naemorhedus baileyi* (I)	+	+									
*Naemorhedus caudatus* (I)	+	+									
